# CLOCK/BMAL1 interactome uncovers homeodomain factors as tissue regulators

**DOI:** 10.1038/s41556-026-02041-4

**Published:** 2026-08-06

**Authors:** Fatih Aygenli, Lukas A. Huschet, Tanja Popp, Andrea Ribeiro, Darina Barkhatova, Céline Jouffe, Riccardo Trozzo, Jerome S. Menet, Roland Rad, Kenneth A. Dyar, Maciej Lech, Tobias Straub, Alicia K. Michael, Maria S. Robles

**Affiliations:** 1 Institute of Medical Psychology, LMU Medizin, Ludwig-Maximilians-Universität München, Munich, Germany; 2 Chair of Molecular Biology, Biomedical Center, LMU Medizin, Ludwig-Maximilians-Universität München, Munich, Germany; 3 School of Medicine, Klinikum Rechts Der Isar, Department of Nephrology, Technical University of Munich, Munich, Germany; 4 Institute of Science and Technology Austria, Klosterneuburg, Austria; 5 Metabolic Physiology, Institute for Diabetes and Cancer, Helmholtz Diabetes Center, Helmholtz Munich, Neuherberg, Germany; 6 German Center for Diabetes Research (DZD), Neuherberg, Germany; 7 Institute of Molecular Oncology and Functional Genomics, School of Medicine, Technische Universität München, Munich, Germany; 8 Center for Translational Cancer Research (TranslaTUM), School of Medicine, Technische Universität München, Munich, Germany; 9 German Cancer Consortium (DKTK), Heidelberg, Germany; 10 Department of Medicine II, Klinikum Rechts der Isar, School of Medicine, Technische Universität München, Munich, Germany; 11 Department of Biology, Center for Biological Clock Research, Texas A&M University, College Station, TX USA; 12 Department of Medicine IV, LMU University Hospital, LMU Medizin, Ludwig-Maximilians-Universität München, Munich, Germany; 13 Bioinformatic Unit, Biomedical Center, LMU Medizin, Ludwig-Maximilians-Universität München, Munich, Germany

**Keywords:** Circadian rhythms, Proteomics

## Abstract

Circadian clocks underlie daily rhythms in physiology by coordinating temporal patterns of gene expression and protein function throughout the body. At the core of this system in mammals is CLOCK/BMAL1, a ubiquitously expressed heterodimeric transcription factor complex that orchestrates tissue-specific circadian gene expression. The basis for this specificity remains unclear, but tissue-specific interactions at chromatin could provide one. Here we used chromatin immunoprecipitation coupled to mass spectrometry to map CLOCK/BMAL1-associated protein complexes on chromatin in mouse liver, kidney and lung. We detected 1,510 associated proteins, most of which were tissue-specific and not explained by protein abundance. Among these, we identified the homeodomain transcription factors PROX1, HNF1B and HOXA5 as tissue-enriched interactors that bind BMAL1, co-occupy most BMAL1 genomic sites and establish organ-restricted circadian transcription. Our findings demonstrate that tissue-specific transcription factors confer cellular identity on the core clock, thereby contributing to organ-specific patterns of rhythmic gene expression.

## Abstract (web summary)

Aygenli et al. map chromatin-bound CLOCK/BMAL1 protein complexes across three mouse tissues and uncover PROX1, HNF1B and HOXA5 as homeodomain cofactors that directly bind and co-occupy BMAL1 sites to establish tissue-specific circadian transcription.

## Main

Transcription factors (TFs) are central regulators of gene expression, directing essential cellular and physiological programmes. Their activities are shaped by context-dependent recruitment to DNA, chromatin state and cooperation with tissue-restricted coregulators, allowing ubiquitous TFs to drive context-specific transcriptional outputs^1^. This is exemplified by the mammalian circadian clock heterodimer, CLOCK/BMAL1, two basic helix–loop–helix (bHLH) TFs which, despite broad expression, produce highly divergent tissue-specific outputs critical for organismal circadian function^2^. Through interlocked transcriptional–translational feedback loops, CLOCK/BMAL1 produces synchronized waves of gene expression, protein abundance and post-translational modifications (PTMs) to maintain homeostasis and synchronize physiology with environmental cues such as light–dark cycles and feeding^3–9^. Although CLOCK/BMAL1 is ubiquitously expressed, circadian transcriptional programmes vary substantially between organs, frequently displaying organ-specific timing. Strikingly, rhythmic CLOCK/BMAL1 binding to genomic sites does not consistently correlate with in-phase or rhythmic transcription, suggesting that DNA occupancy alone is insufficient for rhythmic gene expression^3,10–12^. Structural and computational studies indicate that CLOCK/BMAL1 can function as a pioneer TF, opening chromatin and facilitating access for other regulators^13–15^. Accordingly, CLOCK/BMAL1 probably cooperates with tissue- and time-specific nuclear or chromatin-associated proteins to modulate transcription. Although interaction proteomics has identified several CLOCK/BMAL1 interactors that modulate rhythmic transcription^16–19^, a comprehensive in vivo interaction network for this heterodimer remains unresolved. Defining such complexes will aid in understanding how the core clock heterodimer integrates temporal and tissue-specific cues to drive circadian transcription. Here, we applied an in vivo chromatin immunoprecipitation (IP) combined with mass spectrometry-based quantitative proteomics (ChIP–MS)^20–22^ approach to map spatiotemporal CLOCK/BMAL1 interactions. This analysis uncovered a dynamic, time-resolved, multitissue chromatin interactome, identifying known and previously unrecognized interactors in mouse liver, kidney and lung at three timepoints of the transcriptionally active phase. Among these, we identified three members of the homeodomain TF family, PROX1, HNF1B and HOXA5, that directly bind BMAL1 via its transactivation domain (TAD), co-occupy most BMAL1 genomic sites and modulate CLOCK/BMAL1 activity in a cell lineage-dependent manner.

Together, this study provides a comprehensive chromatin-resolved, in vivo map of CLOCK/BMAL1 complexes and identifies homeodomain TFs as tissue-specific transcriptional modulators, thereby conferring tissue identity to the circadian machinery. Overall, our work illustrates a mechanism by which broadly expressed TFs collaborate with tissue-restricted partners to sculpt transcription, providing a resource and framework for spatiotemporal gene regulation in vivo.

## Results

### Temporal liver CLOCK/BMAL1 chromatin map

**Fig. 1:**
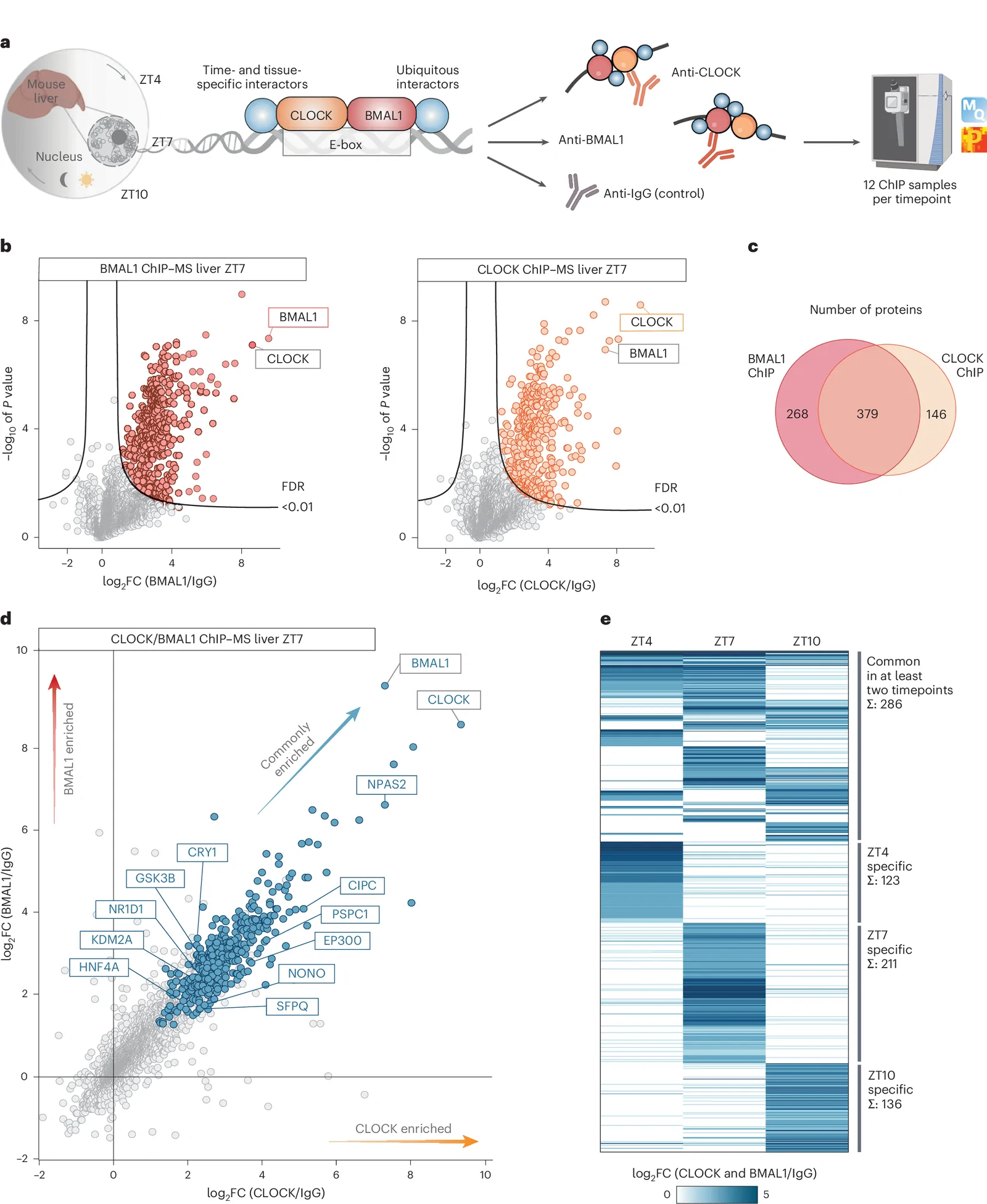
Characterization of the CLOCK/BMAL1 protein interaction landscape at the chromatin site along the circadian transcriptionally active phase in mouse liver. **a**, Graphical representation of the ChIP–MS work­flow used to characterize chromatin-bound CLOCK/BMAL1 protein complexes across the transcriptionally active phase in mouse liver. **b**, Volcano plots representing the results of a two-sided Student’s two-sample *t*-test with permutation-based FDR correction using 250 permutations after comparing the intensities of proteins immunoprecipitated with BMAL1 (left) and CLOCK (right) to IgG (control) antibodies from mouse liver at ZT7. Coloured symbols denote statistically significant proteins defined by our FDR cutoffs (*S*_0_ = 0.5 and FDR <0.01), as indicated by the curved lines. **c**, Venn diagram representing the number of statistically significant proteins found in BMAL1 and CLOCK ChIP–MS immunoprecipitates and their overlap between the two baits. **d**, Scatter plot representing the Student’s *t*-test FC differences in protein intensities in both BMAL1 and CLOCK immunoprecipitates compared with IgG control ones, for common interactors. Labelled proteins correspond to those annotated as ‘rhythmic process’ (GOBP). **e**, Heat map showing the mean value of the log_2_-transformed FCs (Student’s *t*-test) between BMAL1 and CLOCK versus IgG, from the statistically significant immunoprecipitated proteins in mouse liver at ZT4, ZT7 and ZT10. Clustering is based on the statistical significance in the different ZTs. Schematic in **a** created in BioRender; Aygenli, B. https://biorender.com/5eqkwst (2026). Source data

**Extended Data Fig. 1:**
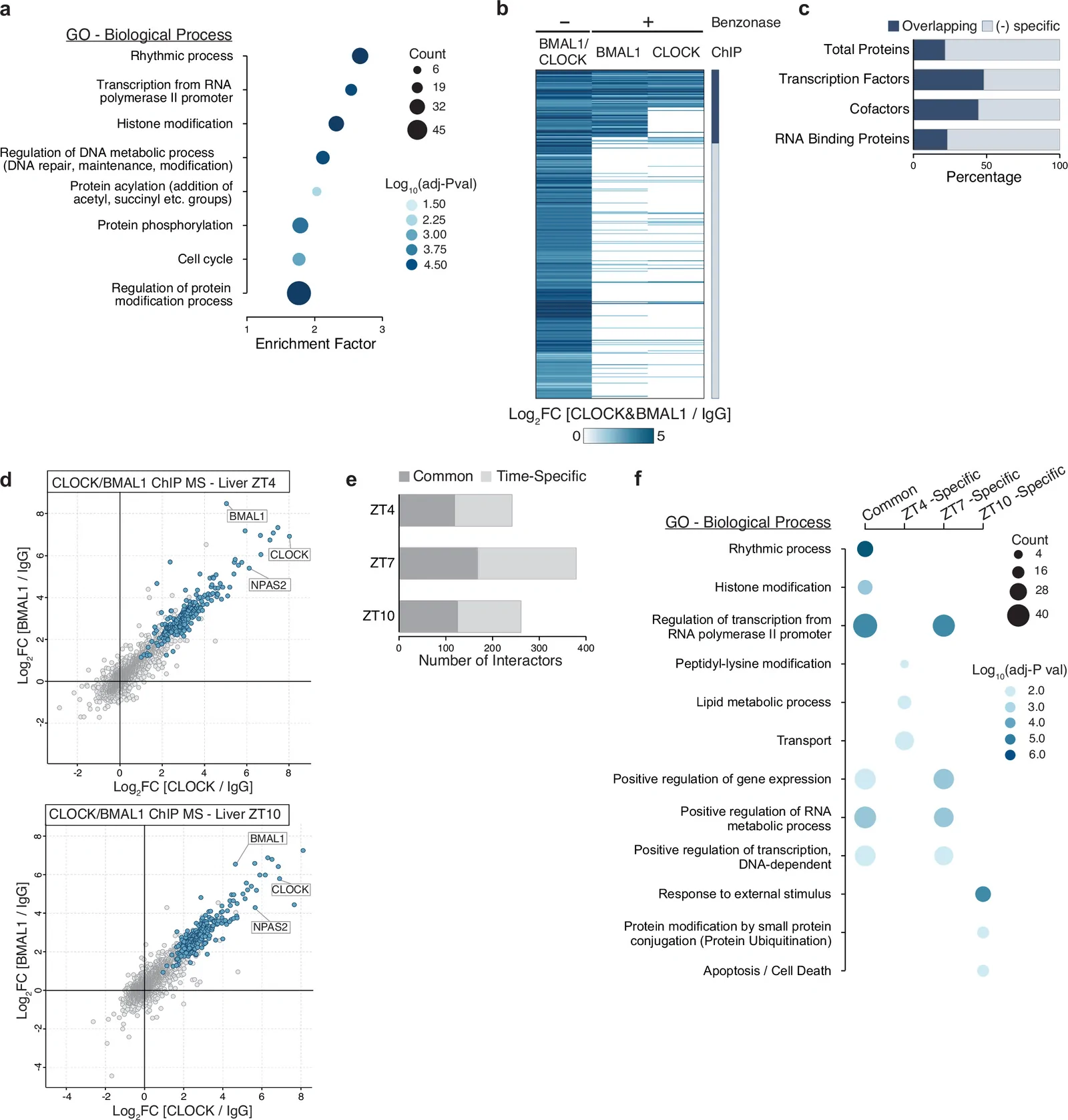
Temporal CLOCK/BMAL1 chromatin interactome at the circadian transcriptionally active phase in the mouse liver. **a**, Gene Ontology Biological Process (GOBP) terms significantly enriched (one-sided Fisher’s exact test against an in-silico mouse gene list background, no adjustment for multiple comparisons, P < 0.05, enrichment factor > 1, intersection size > 5) among those proteins commonly interacting with BMAL1 and CLOCK at chromatin in mouse liver at ZT7. **b**, Heatmap showing differences in protein abundance, assessed by Student’s t-test, between BMAL1/CLOCK and IgG immunoprecipitates in mouse liver at ZT7, with or without benzonase treatment. The left column shows proteins significantly enriched in both BMAL1 and CLOCK immunoprecipitates compared with IgG, whereas the two right columns show proteins significantly enriched in either BMAL1 or CLOCK immunoprecipitates compared with IgG. Proteins are clustered based on their statistical significance in benzonase-treated and untreated samples. **c**, Percentage of proteins significantly enriched in both benzonase-treated and non-treated samples (overlapping) and those exclusively identified in non-treated samples. **d**, Scatter plots showing Student’s t-test differences in protein intensities for common interactors identified in both BMAL1 and CLOCK immunoprecipitates compared with IgG controls in liver at ZT4 (top) and ZT10 (bottom). Colored symbols indicate statistically significant proteins based on the applied cut-offs: FDR < 0.01 and S0 = 0.5. **e**, Barplots representing the number of statistically significant proteins interacting with both BMAL1 and CLOCK at each ZT in mouse liver. Colors denote the number of interactors found in two or three ZTs (common) or exclusively in one ZT (time-specific). **f**, Gene Ontology Biological Process (GOBP) terms significantly enriched (one-sided Fisher’s exact test, *P* < 0.05, Enrichment Factor > 1.75, intersection size > 5) among proteins commonly interacting with BMAL1 and CLOCK in each defined group (from Fig. 1e) based on their temporal interaction pattern. The three most enriched terms are represented for each group. Source data

To investigate the chromatin-associated protein interaction landscape of CLOCK/BMAL1 in mouse tissues, we adapted a method combining ChIP–MS^20^. We first benchmarked our method using mouse livers collected at Zeitgeber time (ZT) 7, corresponding to the peak of CLOCK/BMAL1 rhythmic binding to the chromatin^10^. Nuclear extracts from formaldehyde-crosslinked and sonicated livers were used for IP with commercial antibodies recognizing endogenous BMAL1 and CLOCK, as well as a non-specific IgG serving as a negative control (Fig. 1a; Methods). IP for each antibody was performed in biological quadruplicates and subsequently measured in a label-free, single-shot manner by liquid chromatography–tandem mass spectrometry (LC–MS/MS). Significant interactors were defined by Student’s *t*-test, comparing protein intensities from bait pulldowns against control IgG (Fig. 1b). Both BMAL1 and CLOCK showed the most enrichment in their respective IPs, followed by the heterodimer partner, indicating the specificity of our method. Using a statistical cutoff (*S*_0_ = 0.05, false discovery rate (FDR) <0.01), we defined a confident set of interactors (Fig. 1b). Comparison of the CLOCK and BMAL1 datasets revealed a total of 379 proteins that were significantly immunoprecipitated with both baits, thereby constituting the CLOCK/BMAL1 interactome in mouse liver at ZT7 (Fig. 1c,d and Supplementary Table 1). Among these, we identified known core clock components as well as other proteins previously reported to bind and modulate CLOCK/BMAL1 activity^10,23–25^ (Fig. 1d). In line with this, Gene Ontology enrichment analysis of CLOCK/BMAL1 interactors revealed a statistically significant overrepresentation of proteins involved in rhythmic processes and multiple layers of gene expression regulation, including transcription from RNA polymerase II (RNAPII) promoters, histone modification, DNA metabolic processes and post-transcriptional protein regulation (Extended Data Fig. 1a). These mechanisms are known to exhibit temporal dynamics and have been linked with CLOCK/BMAL1 function on chromatin at the peak of circadian transcriptional activity. To experimentally assess whether CLOCK/BMAL1 interactions occur at the chromatin site, where BMAL1 and CLOCK are maximally binding at this timepoint, we repeated the ChIP–MS, including a benzonase treatment to degrade nucleic acids. Under this condition, we observed a dramatic reduction in the number of co-immunoprecipitated proteins with both CLOCK and BMAL1, indicating that most interactions are either chromatin associated and/or are mediated by nucleic acids (Extended Data Fig. 1b,c and Supplementary Table 1). Notably, benzonase treatment resulted in the loss of a large fraction of RNA-binding proteins while only approximately half of the TFs and cofactors were affected.

To capture dynamic interactions of proteins with CLOCK/BMAL1 at the chromatin site during the transcriptionally active phase, we next applied our approach to mouse livers collected at ZT4 and ZT10, corresponding to the early (derepression) and late circadian transcriptionally active phase, respectively^10^. Using the same statistical cutoff as in ZT7, 242 and 261 interactors were defined with both baits at ZT4 and ZT10, respectively, with BMAL1 and CLOCK being among the most significantly enriched proteins (Extended Data Fig. 1d). Comparative analyses of all three timepoints revealed a temporal-resolved CLOCK/BMAL1 interactome of 756 proteins during the transcriptionally active phase, of which 286 (38%) were identified in at least two circadian timepoints and 470 (62%) were unique to a single circadian timepoint (Fig. 1e and Supplementary Table 2). This indicates that the chromatin-associated CLOCK/BMAL1 interactome is temporally regulated, consistent with previous reports describing rhythmic CLOCK/BMAL1 DNA occupancy^10,26^, time-dependent interaction of EP300 and CRY1 with BMAL1^24^ and dynamic chromatin accessibility at circadian enhancers^27^. ZT7 contained the largest number of interactions (Fig. 1e and Extended Data Fig. 1e), coinciding with the extensive CLOCK and BMAL1 genomic occupancy in mouse liver at this circadian timepoint^12^. Interactors present at all timepoints and ZT7-specific interactors were significantly enriched for rhythmic processes associated with circadian transcription, including RNA metabolism and transcriptional regulation by RNAPII (Extended Data Fig. 1f and Supplementary Table 2). By contrast, ZT4-specific interactors were enriched for nuclear transport components such as nuclear pore proteins and peptidyl-lysine modifiers (for example, HDAC2, EHMT2 and SUMO1), pointing to a potential regulation of CLOCK/BMAL1 nuclear import and post-transcriptional modification in the early transcriptionally active phase. Conversely, E3 ubiquitin ligases were predominantly enriched at ZT10, suggesting a regulation of protein stability and degradation at the late transcriptionally active phase (Extended Data Fig. 1f). Together, these findings demonstrate the robustness and utility of our ChIP–MS approach for efficiently capturing in vivo DNA-bound CLOCK/BMAL1 protein interactions and uncovering their dynamic composition across the transcriptionally active phase in mouse liver.

### Spatiotemporal CLOCK/BMAL1 interactome

**Fig. 2:**
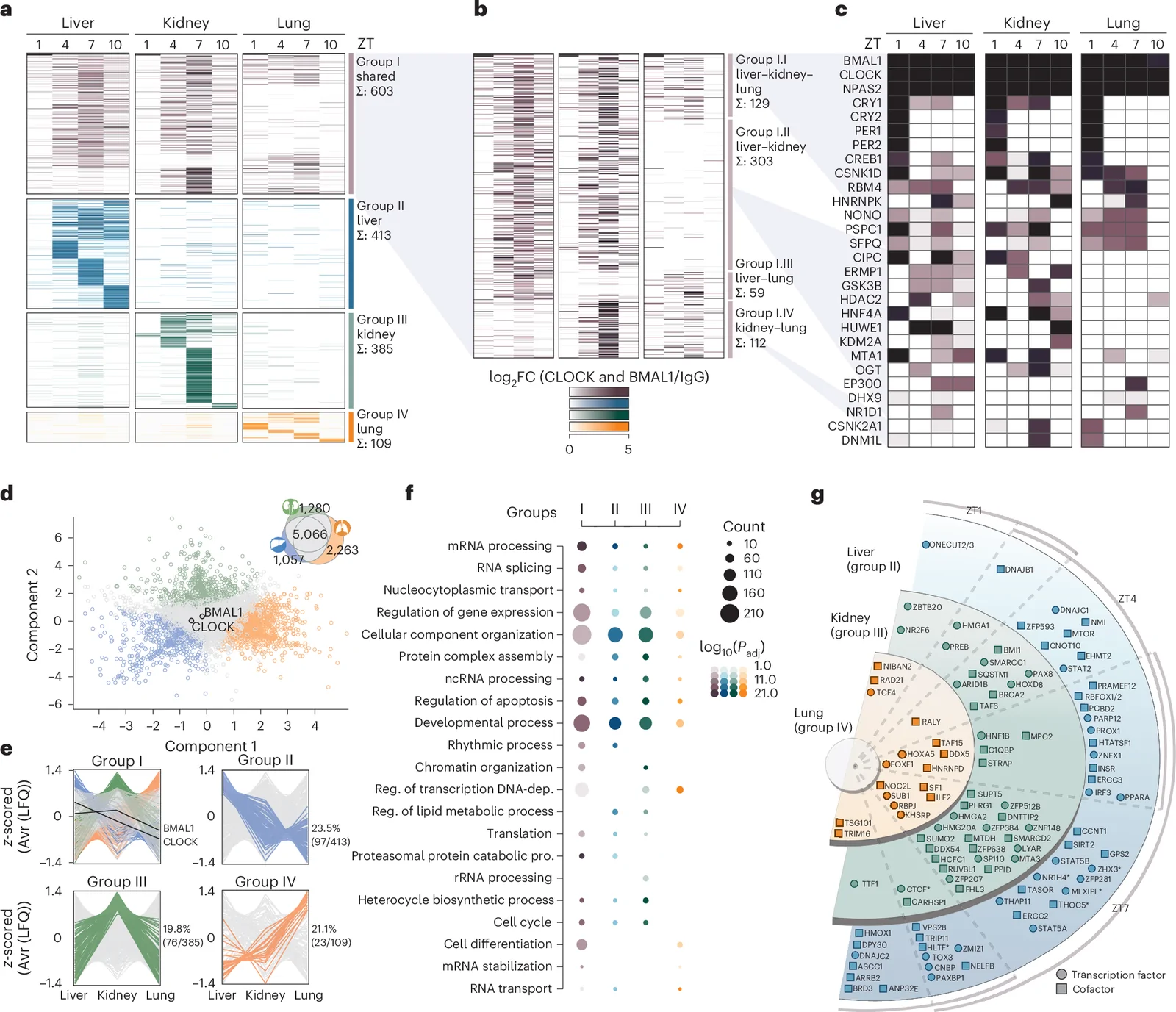
Spatiotemporal protein interaction landscape of CLOCK/BMAL1 on chromatin in mouse tissues along the circadian transcriptionally active phase. **a**, Heat map showing the mean protein abundance differences resulting from Student’s *t*-test comparing BMAL1 and CLOCK with IgG ChIP–MS for statistically significant immunoprecipitated proteins in mouse liver, kidney and lung at ZT1, ZT4, ZT7 and ZT10. Proteins are grouped and colour coded on the basis of statistical significance across tissues: shared interactors in at least two tissues (group I) or tissue-specific interactors (group II liver, group III kidney and group IV lung). **b**, Zoom-in of group I from **a**. Proteins are subgrouped on the basis of statistical significance in the different tissue combinatorial manner. **c**, Heat map with a selection of proteins contained in **b** based on their annotation as ‘rhythmic process’ (GOBP). **d**, PCA of protein intensities quantified in nuclear input samples for each tissue. Proteins significantly enriched in a tissue-specific manner (ANOVA multiple sample test, *S*_0_ = 0, FDR <0.001) are colour coded. BMAL1 and CLOCK are labelled. Top right: Venn diagram representing the number of tissue-enriched and common proteins across the three tissues on the basis of the ANOVA test results (post hoc Tukey’s HSD test, FDR <0.05). **e**, Line plots showing protein intensities (*z-*scored average (Avr) LFQ intensities) in each tissue nuclear input for those proteins in the groups defined in **a**. Proteins with statistically significant differences across tissue nuclear proteomes (from **d**) are colour coded as in **d**. **f**, GOBP enriched terms among proteins in each defined group from **a**. **g**, Graphical representation of TFs and cofactors found to interact with CLOCK/BMAL1 on chromatin at the different ZTs in the three tissues. Proteins identified in two consecutive ZTs are shown in the overlapping areas denoted by dashed lines. *Proteins identified in three timepoints. Reg., regulation; dep., dependent; pro., process. Source data

**Extended Data Fig. 2:**
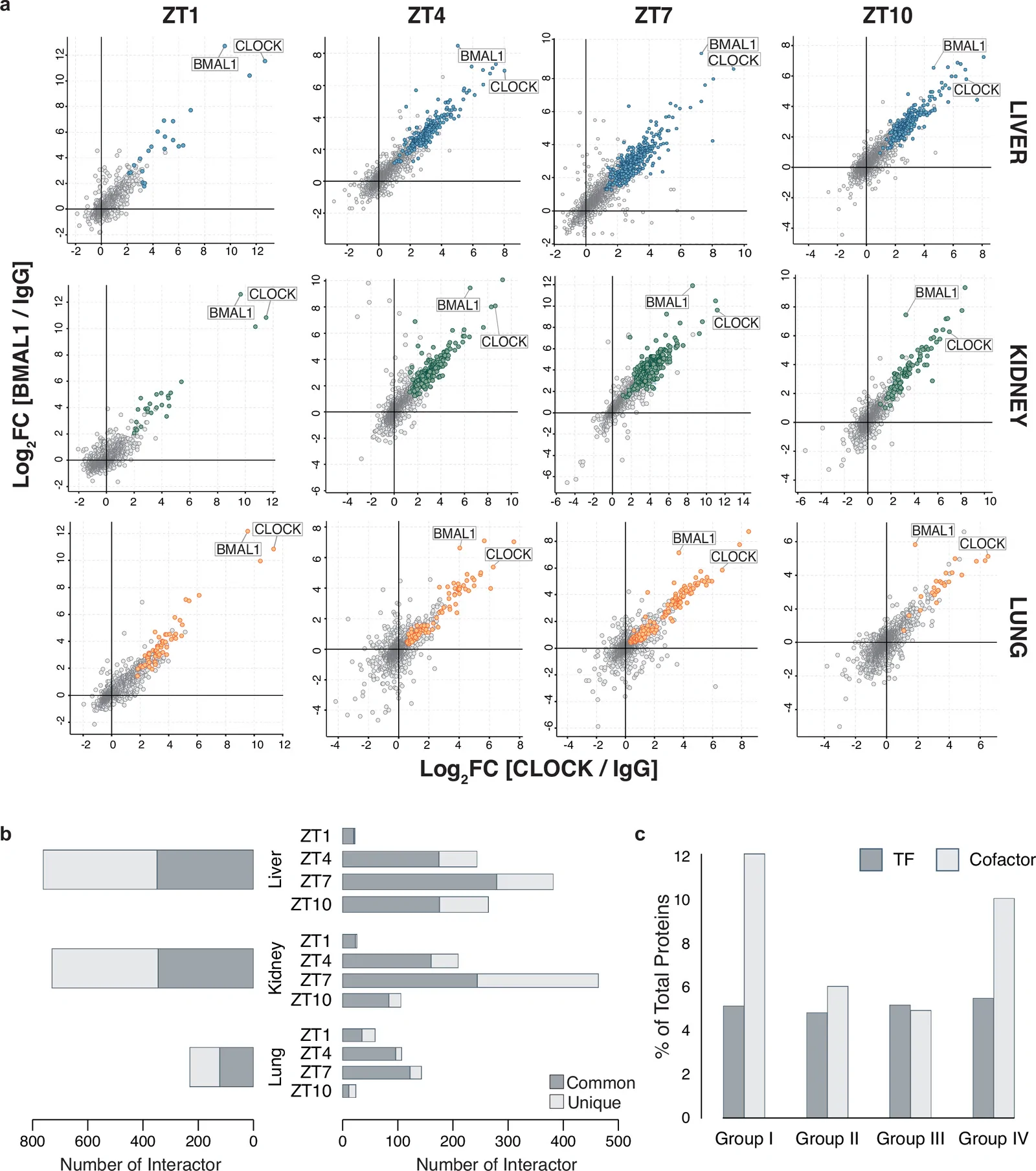
Distinct chromatin interaction profiles of CLOCK/BMAL1 across tissues. **a**, Scatter plots representing Student’s t-test differences in protein intensities for both BMAL1 and CLOCK compared to IgG control immunoprecipitates, for common interactors in liver, kidney and lung at each ZT. Colors represent the two FDR-based confidence significant cut-offs. **b**, Bar plots represent the number of statistically significant proteins interacting with both BMAL1 and CLOCK in each tissue and at each ZT. Colors in each bar denote the number of interactors found in two or all three tissues (common) or exclusively detected in one tissue (unique). **c**, Bar plots show the percentage of the total CLOCK/BMAL1 interactors represented by the transcription factors and cofactors (a total of 79 and 128, respectively) across tissue-defined groups (I, II, III and IV). Source data

Although all tissues share the same circadian mechanism, CLOCK/BMAL1 cistromes and circadian transcriptional outputs exhibit remarkable tissue specificity. Recent studies indicate that circadian gene expression is shaped by the crosstalk between the CLOCK/BMAL1 complex and both ubiquitous and tissue-specific transcriptional regulators, including TFs, cofactors and chromatin modifiers^2,3,10–12,15^. To experimentally investigate this, we next extended our ChIP–MS workflow to mouse kidney and lung with the aim of identifying tissue-specific CLOCK/BMAL1 interactors. These tissues were selected on the basis of two criteria: (1) after the liver, they display the largest number of oscillating transcripts^2^ and (2) the circadian clock is essential for their physiology^28–30^. Together, we performed ChIP–MS on mouse liver, kidneys and lungs collected at ZT1, ZT4, ZT7 and ZT10 using anti-CLOCK, anti-BMAL1 and non-specific IgG antibodies. Proteins interacting with both CLOCK and BMAL1 were defined using the same statistical criteria as in the liver. Consistently and as shown in the liver, our approach robustly immunoprecipitated both baits and reciprocal partners, which ranked among the most statistically significant and enriched interactors (Extended Data Fig. 2a). Collective analysis of all ChIP–MS (*n* = 144) experiments across tissues and timepoints identified 1,510 proteins significantly immunoprecipitated with both baits. Two-thirds of interactors displayed tissue-specificity, while approximately one-third displayed preferential or overlapping presence in any of the three tissues at one or more circadian timepoints (Fig. 2a,b, Extended Data Fig. 2b and Supplementary Table 3), with ZT7 as the time with the largest number of interactions in the three tissues (Fig. 2a and Extended Data Fig. 2b). This corresponds to the peak of CLOCK/BMAL1 chromatin binding in the liver^10^ and our data suggest a parallel—so far uncharacterized—temporal pattern in the kidney and lung. Consistent with the conserved core clock machinery, proteins with core circadian function were among the interactors shared across tissues (Fig. 2c). Among the tissue-specific interactors, the liver exhibited the largest number, followed by kidney and lung (Fig. 2a and Extended Data Fig. 2b), a distribution that directly correlates with the number of circadian rhythmic transcripts and CLOCK/BMAL1 cistromes^2,11,26^. To determine whether tissue-specific interactors could be explained by differences in protein abundance, we performed label-free quantitative proteomics on nuclear input samples for each tissue. Principal component analysis (PCA) showed that nuclear proteomes were clustered primarily by tissue, with over 10% of proteins showing statistically significant differences between tissues, while CLOCK and BMAL1 levels were consistent across organs (Fig. 2d and Supplementary Table 4). Among all CLOCK/BMAL1 tissue-specific interactors, strikingly, only around 20% showed differential nuclear abundance in the tissue where the interaction with the CLOCK/BMAL1 complex was detected. This suggests that organ-specific interactions are not limited to nuclear proteins with exclusive or preferential expression (Fig. 2e).

To better understand the functional relevance of shared and tissue-specific interactors, we performed enrichment analysis on each interaction group (Fig. 2a). As expected, proteins interacting in all tissues were associated with canonical CLOCK/BMAL1 functions, including rhythmic processes, regulation of gene expression and mRNA processing (Fig. 2f and Supplementary Table 3). Interestingly, these terms were likewise enriched in tissue-specific clusters, indicating that CLOCK/BMAL1 complexes cooperate with organ-specific transcription regulators to shape temporal and tissue-specific transcription. Consistently, across tissues, 5–10% of interactors represent TFs or cofactors, many of them displaying tissue specificity (Fig. 2g and Extended Data Fig. 2c). Collectively, our spatiotemporal analysis demonstrates that the chromatin-associated protein interaction landscape of CLOCK/BMAL1 is shaped predominantly by tissue context rather than circadian time, at least within the timepoints examined here, and cannot be explained solely by tissue-restricted protein nuclear abundance. Both shared and tissue-specific interactors comprise known and novel identified transcription regulators that may modulate CLOCK/BMAL1 function and thereby contribute to the generation of tissue-specific circadian transcriptional outputs.

### Organ-specific interactions of homeodomain TFs

**Fig. 3:**
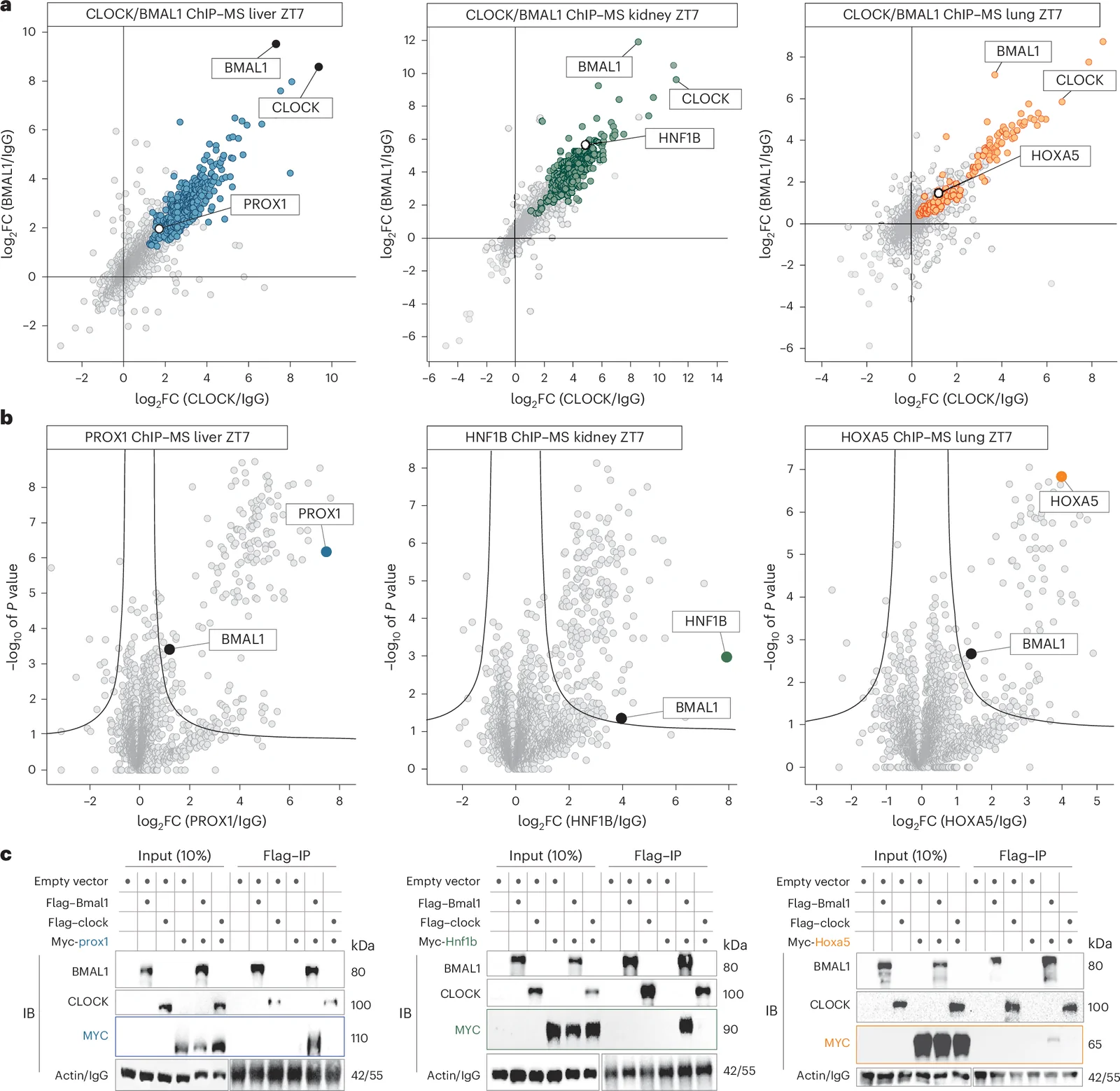
The homeodomain TFs, PROX1, HNF1B and HOXA5, as tissue-specific CLOCK/BMAL1 interactors on chromatin. **a**, Scatter plots, as shown in Extended Data Fig. 2a, highlight the bait proteins CLOCK and BMAL1 as well as the homeodomain TFs PROX1, HNF1B and HOXA5 in the liver, kidney and lung, respectively. **b**, Volcano plots representing two-sided Student’s two-sample *t*-test results comparing protein intensities from ChIP–MS with PROX1 in the liver (left), HNF1B in the kidney (middle) and HOXA5 in the lung (right) at ZT7 compared with IgG controls. Curve lines are determined by FDR <0.01 and *S*_0_ = 0.5. **c**, Immunoblots showing co-IP results performed after transiently co-expressing Flag-tagged CLOCK and BMAL1 proteins with MYC-tagged PROX1, HNF1B and HOXA5 proteins in HEK293T cells. Actin and IgG were used as loading controls for input or IP samples, respectively, in the same gel. Source data

**Extended Data Fig. 3:**
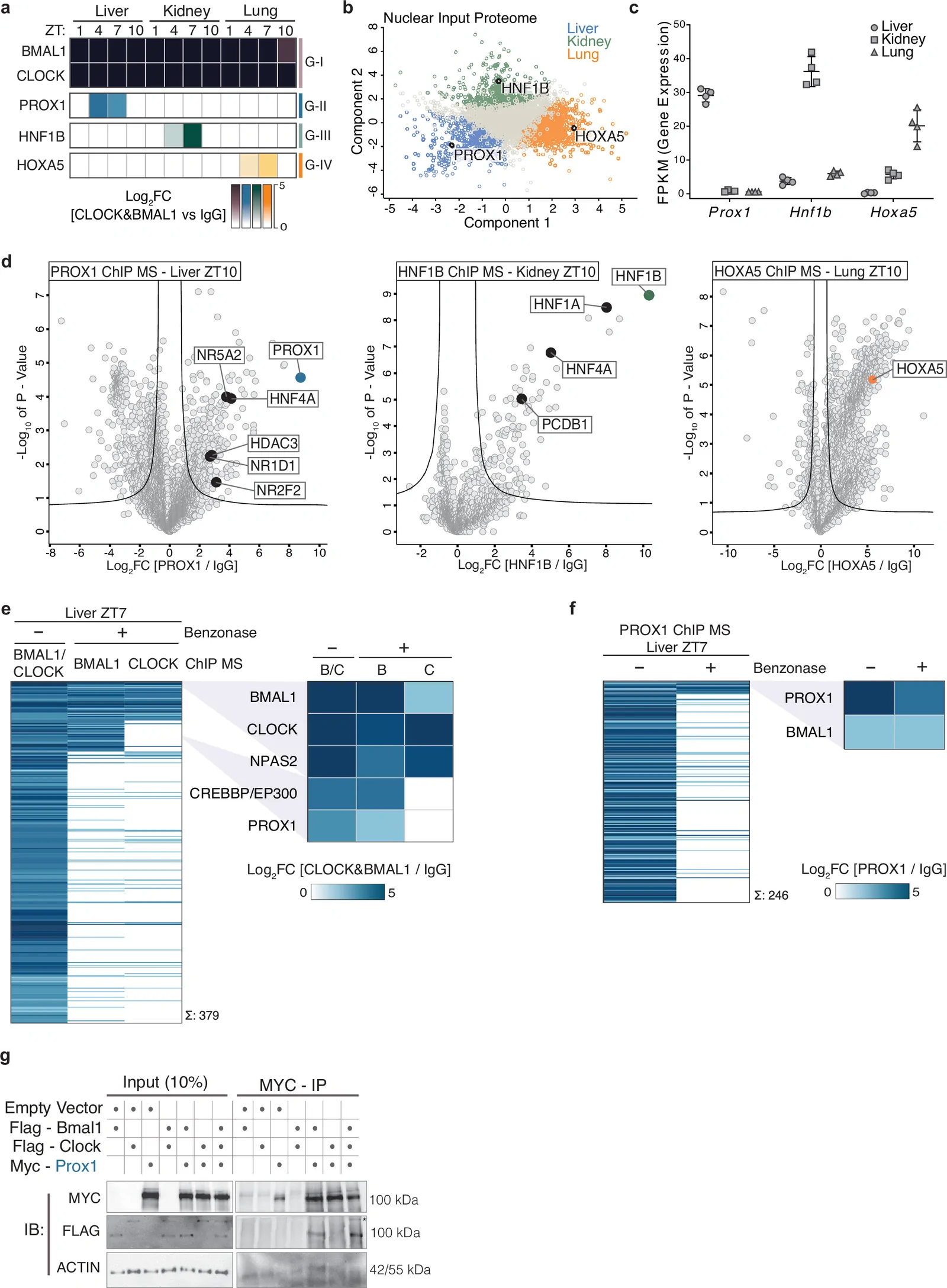
BMAL1 interacts with PROX1, HNF1B and HOXA5 independently of chromatin. **a**, Heatmap showing the mean abundance differences of BMAL1, CLOCK, PROX1, HNF1B and HOXA5 resulting from the Student’s t-test comparing BMAL1 and CLOCK versus IgG ChIP-MS in mouse liver, kidney and lung at ZT1, ZT4, ZT7 and ZT10. White indicates no statistical significance or no detection. **b**, Scatter plot of the Principal Component Analysis as in Fig. 2d highlighting the position of the homeobox transcription factors, PROX1, HNF1B and HOXA5. **c**, Normalized expression levels of Prox1, Hnf1b and Hoxa5 in liver, kidney and lung from^34^. Horizontal lines indicate the mean and error bars the ± s.d.; n = 4 biologically independent samples per tissue. **d**, Volcano plots representing two-sided Student’s t-test results with permutation-based FDR correction using 250 permutations comparing protein intensities from ChIP-MS with PROX1 in liver (left), HNF1B in kidney (middle) and HOXA5 in lung (right) at ZT10 compared to IgG controls. Curved lines are determined by FDR < 0.01 and S0 = 0.5. **e**, Heatmap showing the Student’s t-test protein abundance difference calculated as a mean for BMAL1 and CLOCK versus IgG (left column) or BMAL1 or CLOCK versus IgG (right two columns) ChIP-MS for statistically significant immunoprecipitated proteins in mouse liver at ZT7 in the presence or absence of benzonase. Right panel displays the values for selected proteins. **f**, As in **e**, but with the Student’s t-test results comparing PROX1 with IgG ChIP-MS of liver at ZT7 in the presence or absence of benzonase. The right panel displays the values for the bait itself, PROX1 and BMAL1. **g**, Immunoblots showing co-immunoprecipitation results performed after transiently co-expressing FLAG-tagged CLOCK and BMAL1 proteins with MYC-tagged PROX1 proteins in HEK293T cells. ACTIN and IgG were used as loading controls for input samples. Source data

To assess the functional relevance of tissue-specific CLOCK/BMAL1 interactors, we focused on three homeodomain TFs: prospero-related homeobox 1 (PROX1) in the liver, the hepatocyte nuclear factor-1 beta (HNF1B) in the kidney and homeobox protein Hox-A5 (HOXA5) in the lung. These factors were identified within a defined temporal window, between ZT1 and ZT10, coinciding with the peak of CLOCK/BMAL1 transcriptional activity (Figs. 2g and 3a and Extended Data Fig. 3a). In addition to their shared dynamic interaction, these factors have well-established roles in developmental processes and cell identity maintenance in the organs in which they were identified^31–33^, making them strong candidates for mediating tissue-specific transcriptional programmes.

Although transcripts^34^ and proteins from these factors are found in the three tissues, their levels were highly enriched in the tissue in which the interaction was identified (Extended Data Fig. 3b,c). PROX1, HNF1B and HOXA5 can act as canonical DNA-binding factors via their homeodomain and as transcriptional coregulators through direct protein–protein interaction in diverse contexts^31,35–38^. Notably, PROX1 has been linked to circadian regulation in the liver via nuclear receptor binding, but no direct interaction with CLOCK/BMAL1 has been reported^35,39^. This led us to further investigate the interaction between these homeodomain factors and the CLOCK/BMAL1 heterodimer complex. Using reciprocal ChIP–MS at ZT7, we confirmed that all three proteins co-immunoprecipitated BMAL1 in the respective tissues (Fig. 3b and Supplementary Table 5). However, CLOCK was not detected, suggesting a preferential interaction with BMAL1. Benzonase treatment did not disrupt the BMAL1–PROX1 interaction, as observed for other direct BMAL1 interactors such as CLOCK, NPAS2 and EP300, indicating that this binding is not mediated by nucleic acids. By contrast, PROX1 was not detected in CLOCK ChIP–MS after benzonase treatment, such as EP300, which directly binds BMAL1 but not CLOCK^24^ (Extended Data Fig. 3e,f and Supplementary Tables 1 and 5). This BMAL1-dependent interaction was further supported by co-IP assays: PROX1 efficiently co-immunoprecipitated both BMAL1 and CLOCK only when all three proteins were co-expressed. By contrast, in the absence of BMAL1, PROX1 failed to co-immunoprecipitate CLOCK. Moreover, BMAL1, but not CLOCK alone, consistently co-immunoprecipitated PROX1, HNF1B and HOXA5 (Fig. 3c and Extended Data Fig. 3g). To further examine the temporal specificity of PROX1, HNF1B and HOXA5 interactions in vivo, we performed reciprocal IP experiments at ZT10 in the respective tissues. Consistent with our CLOCK/BMAL1 ChIP–MS data at this timepoint, none of the homeodomain factors co-immunoprecipitated BMAL1 or CLOCK, whereas previously reported partners were detected^35,40^, corroborating the results from the experiments described above (Extended Data Fig. 3d and Supplementary Table 5).

Collectively, these findings demonstrate that the homeodomain factors PROX1, HNF1B and HOXA5 interact with the positive arm of the circadian clock in a tissue-specific manner within a defined time window corresponding to the peak transcriptional phase of CLOCK/BMAL1 activity, with this interaction preferentially mediated by BMAL1.

### Genomic overlap of BMAL1 and homeodomain TFs

**Fig. 4:**
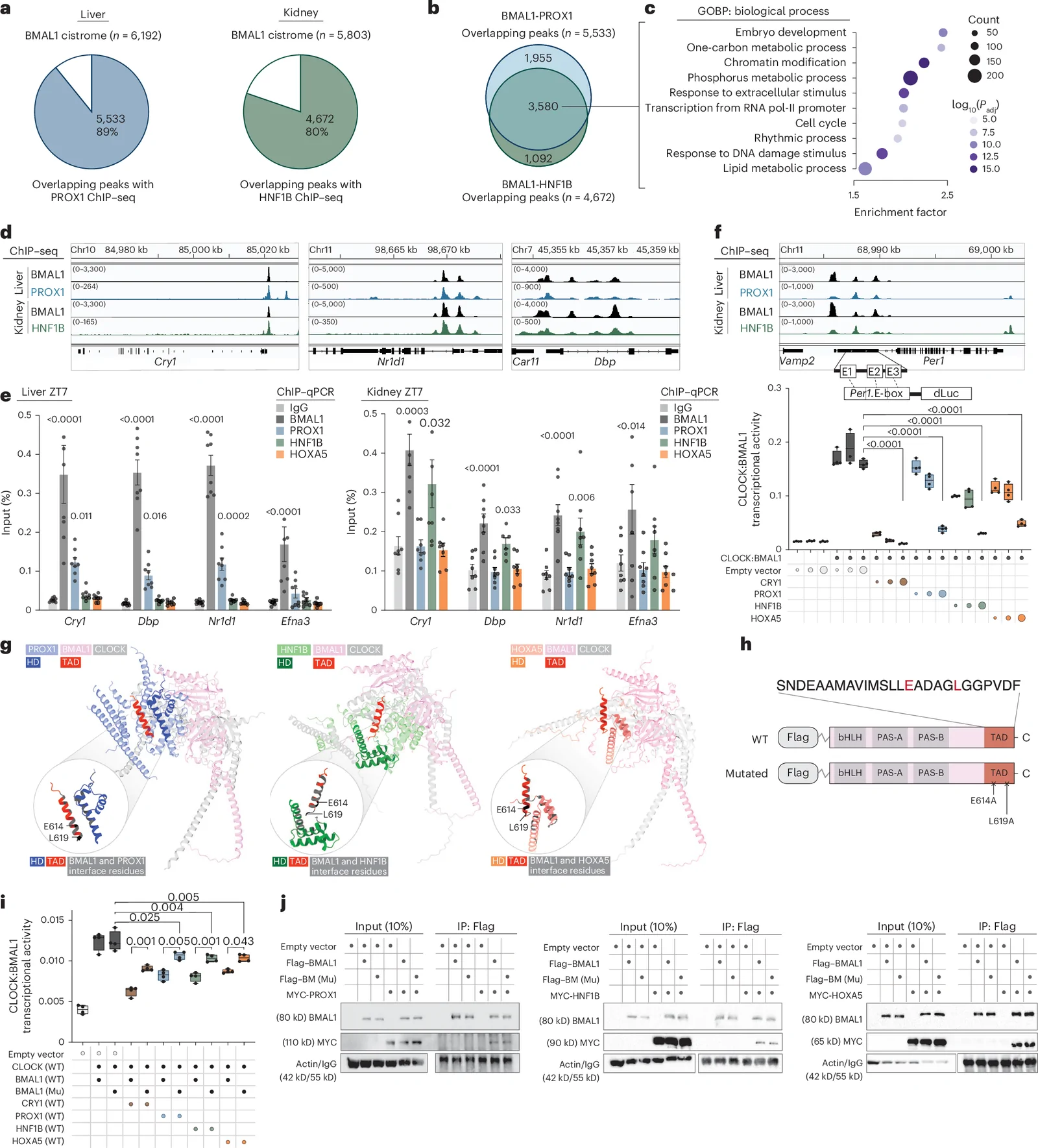
PROX1, HNF1B and HOXA5 colocalize with BMAL1 at genomic sites and repress CLOCK/BMAL1 transcriptional activity. **a**, The overlap between BMAL1 and PROX1 (left) and BMAL1 and HNF1B (right) ChIP–seq peaks in the mouse liver and kidney, respectively. **b**, The overall overlap between co-occupied genomic sites of BMAL1-PROX1 in the liver and those co-occupied by BMAL1-HNF1B in the kidney. **c**, GOBP enriched terms from the genes nearest to the co-occupied genomic sites from **b**. Enrichment was assessed using Fisher’s exact test, and no adjustment for multiple comparisons was made. Terms with *P* < 0.01 and an enrichment factor >1 were considered. **d**, IGV views of BMAL1 (black), PROX1 (blue) and HNF1B (green) occupancies at the *Cry1*, *Nr1d1* and *Dbp* loci in the indicated tissue. **e**, BMAL1, PROX1, HNF1B, HOXA5 and IgG control signals (percentage input) at *Cry1*, *Dbp*, *Nr1d1* and *Efna3* genes in the mouse liver (left) and kidney (right) at ZT7 analysed by ChIP–qPCR. Values are shown as mean ± s.e.m. of *n* = 7, 7, 8, 8 and 8 biologically independent samples for the 5 conditions shown from left to right. Statistical significance was assessed by one-way ANOVA; numbers denote *P*_adj_ values. **f**, Top: IGV views of BMAL1 (black), PROX1 (blue) and HNF1B (green) occupancies at the *Per1* genomic locus in the indicated tissue. Bottom: dose-dependent effect of PROX1, HNF1B and HOXA5 on CLOCK/BMAL1 transactivation of a luciferase gene driven by *Per1* E-boxes. Box plots show the median, 25th and 75th percentiles, and whiskers denote minimum to maximum values. Individual data points for *n* = 4 (*n* = 3 for the fifth line) biologically independent samples are displayed. Statistical significance was assessed by one-way ANOVA and the resulting *P*_adj_ are shown. **g**, AlphaFold 3 predictions of CLOCK/BMAL1 with the factors (left to right): PROX1 (blue) iPTM, 0.39; HNF1B (green) iPTM, 0.41; and HOXA5 (orange) iPTM, 0.49. The BMAL1-TAD is denoted in red and the homeodomains (HD) are denoted in darker colours. Bottom magnifications display BMAL1 residues in the TAD domain predicted to interact with the respective factor. iPTM scores were defined. Disordered regions (low-confidence prediction regions, B-factor <25) are hidden. **h**, Schematic illustration of the predicted amino acid residue interphases (in red) between PROX1, HNF1B and HOXA5 and the BMAL1 TAD domain. The sequence highlights two BMAL1 TAD residues (E614 and L619) predicted to mediate the interaction with these HD TFs and the tested mutation version. **i**, Effect of the BMAL1 E614A and L619A point mutations on the PROX1, HNF1B, HOXA5 and CRY1 repression effect of CLOCK/BMAL1 transactivation; as in **f**, box plots show the median, 25th and 75th percentiles, and the whiskers show the minimum to maximum values. Individual data points are displayed. *P*_adj_ values resulting from a one-way ANOVA are indicated in the plot. **j**, Immunoblots showing co-IP of transiently expressed Flag-tagged WT, E614A and L619A BMAL1 together with CLOCK and MYC-tagged PROX1, HNF1B and HOXA5 proteins in HEK293T cells. Actin and IgG were used as loading control in input and co-IP, respectively, in the same gel. Source data

**Extended Data Fig. 4:**
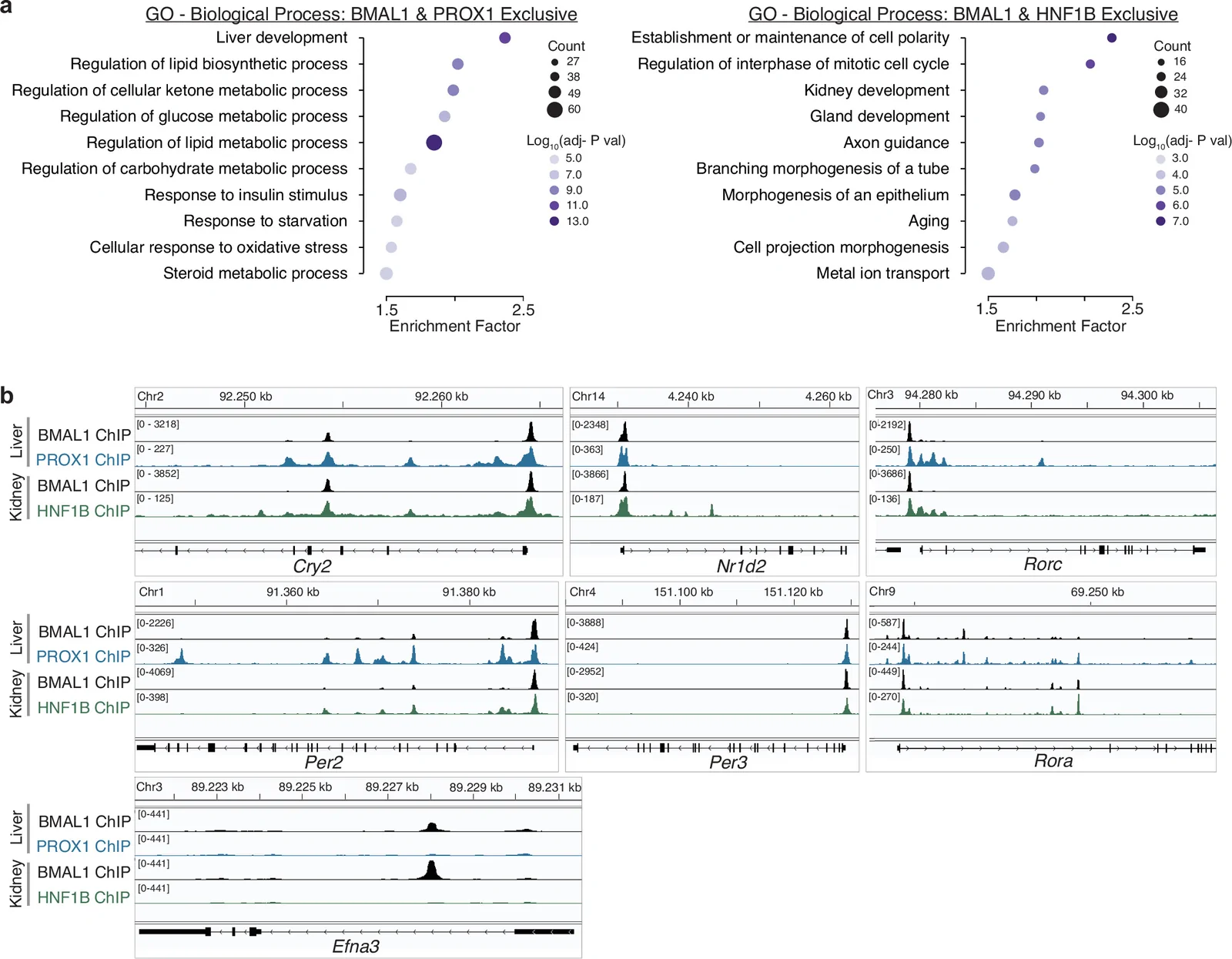
BMAL1 binding sites at genomic loci of circadian core clock genes are co-occupied by PROX1 and HNF1B. **a**, GOBP terms enriched for genes nearest exclusively co–occupied genomic sites of BMAL1 and PROX1 in the liver (left) and BMAL1 and HNF1B in kidney (right) from Fig. 4b. One-sided Fisher’s exact test against an in silico mouse gene list was applied, no adjustment for multiple comparisons, P < 0.05, enrichment factor > 1. **b**, IGV genome browser view of BMAL1 (black), PROX1 (blue) and HNF1B (green) occupancies at the loci of *Cry2, Nr1d2, Per2, Per3, Rora, Rorc* and *Efna3* genes in the indicated tissues. Source data

Having shown that PROX1, HNF1B and HOXA5 interact with BMAL1, we next examined whether these homeodomain factors colocalize with BMAL1 at genomic sites in a tissue-specific manner. To this end, we performed chromatin IP followed by sequencing (ChIP–seq) for BMAL1 and either PROX1 or HNF1B in mouse liver or kidney, respectively. Analysis of BMAL1 cistromes in both tissues revealed extensive co-occupancy: 89% of BMAL1-bound genomic sites in the liver at ZT7 and 80% in the kidney at ZT8 were also occupied by PROX1 and HNF1B, respectively (Fig. 4a and Supplementary Table 6). These co-occupied sites overlapped substantially between tissues, with PROX1 and HNF1B colocalizing with BMAL1 at largely shared genomic loci but in a tissue-exclusive manner (Fig. 4b). The overlapping peaks were enriched for genes involved in circadian processes, such as rhythmic function, lipid metabolism, transcriptional regulation and DNA damage response (Fig. 4c). By contrast, tissue-exclusive co-occupied sites overrepresented organ-specific functions: lipid and glucose metabolism genes in the liver and genes regulating kidney morphogenesis and development in the kidney (Extended Data Fig. 4a). These tissue processes have been previously attributed to PROX1 and HNF1B functions in the respective tissue^35,41,42^. Furthermore, genome browser inspection also revealed a striking, organ-specific colocalization of BMAL1 with PROX1 or HNF1B at core clock genes such as *Cry1*, *Nr1d1* (*Rev-erbα*) and *Dbp* (Fig. 4d and Extended Data Fig. 4b). Tissue-specific colocalization on selected genes was further confirmed by ChIP–quantitative PCR (qPCR), corroborating exclusive co-occupancy of BMAL1 with PROX1—but not HNF1B or HOXA5—in liver sites, and BMAL1 and HNF1B—but not PROX1 or HOXA5—in kidney sites (Fig. 4e). Together, our data demonstrate that homeodomain TFs co-occupy a substantial fraction of the BMAL1 cistrome, including core clock genes, in a tissue-specific manner, pointing to a role in modulating tissue-specific circadian transcription.

### Homeodomain TFs repress CLOCK/BMAL1 activity

To test whether PROX1, HNF1B and HOXA5 influence CLOCK/BMAL1 transcriptional activity, we performed transactivation assays using a luciferase reporter construct containing three tandem E-boxes from the *Per1* promoter sites, that we found co-occupied in vivo by BMAL1 and PROX1 or HNF1B (Fig. 4f). Co-expression of PROX1, HNF1B and HOXA5 with CLOCK/BMAL1 significantly reduced reporter activity in a dose-dependent manner, comparable to the canonical repressor CRY1 (Fig. 4f). These results demonstrate that PROX1, HNF1B and HOXA5 act as transcriptional repressors of CLOCK/BMAL1 on *Per1* promoter, in line with previously reported transcriptional repression function of PROX1 in other contexts^35,36^. This repressive function is not mediated by binding of those factors to alternative motifs at the DNA but rather to E-box-bound CLOCK/BMAL1, consistent with our data showing direct interactions between PROX1, HNF1B and HOXA5 with BMAL1 in the absence of chromatin (Fig. 3c and Extended Data Fig. 3f,g).

**Extended Data Fig. 5:**
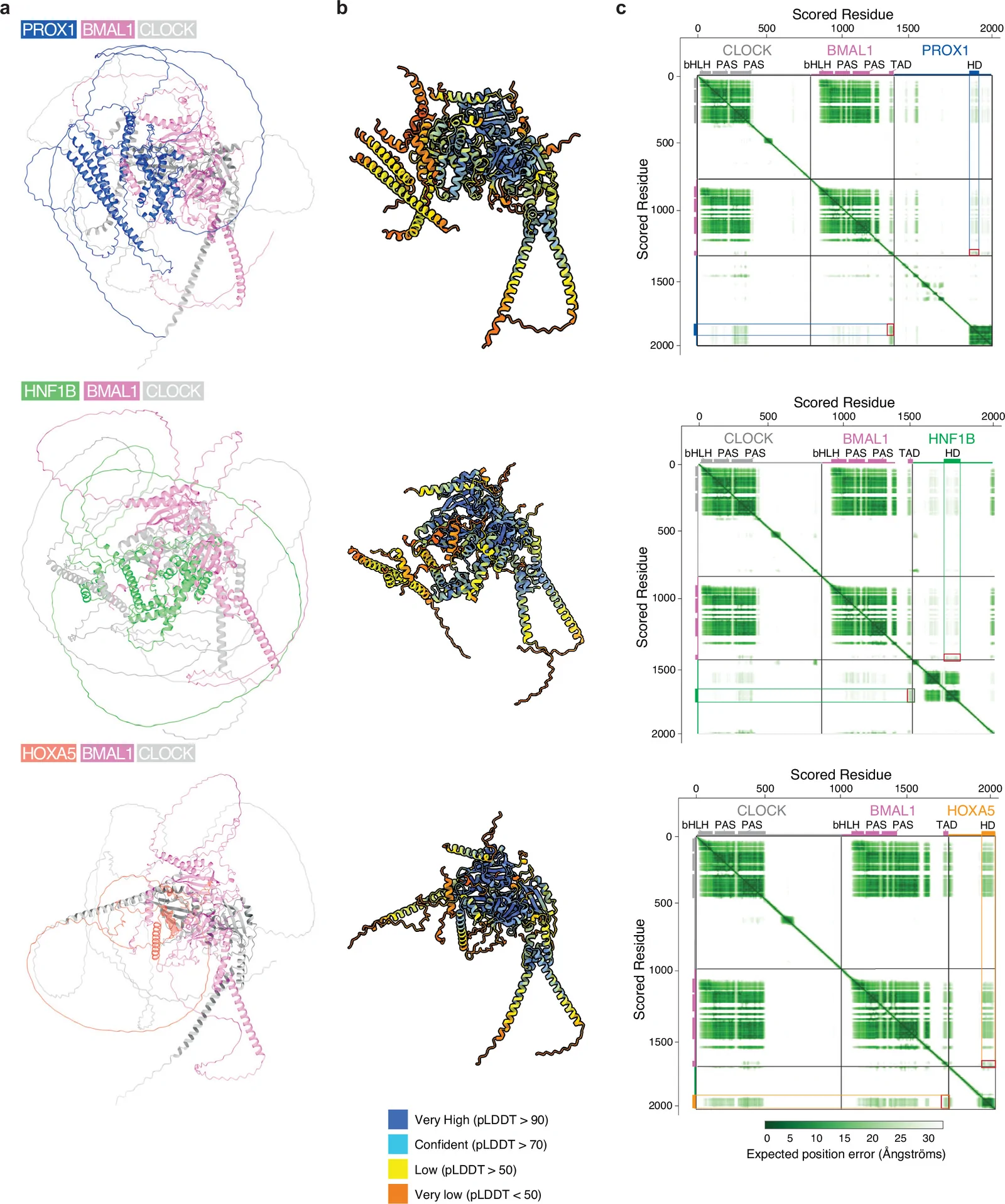
Alphafold3 prediction of the interaction between CLOCK/BMAL1 complex and homeobox transcription factors; PROX1, HNF1B and HOXA5. **a**, AlphaFold3 structural predictions for CLOCK/BMAL1 complexes with, top to bottom, PROX1 (blue), HNF1B (green) and HOXA5 (orange). **b**, AlphaFold3 structural predictions for CLOCK/BMAL1 complexes with, top to bottom, PROX1, HNF1B and HOXA5 colored according to the pLDDT score (per-atom confidence estimate on a 0-100 scale with higher value indicating higher confidence). **c**, Predicted Aligned Error (PAE) matrices of predictions (darker is more confident), with protein coloring on the side bars. The black lines indicate the chain boundaries. The homeodomain-BMAL1 interaction regions are marked with boxes. The homeodomain-TAD interactions are in red boxes. Source data

**Extended Data Fig. 6:**
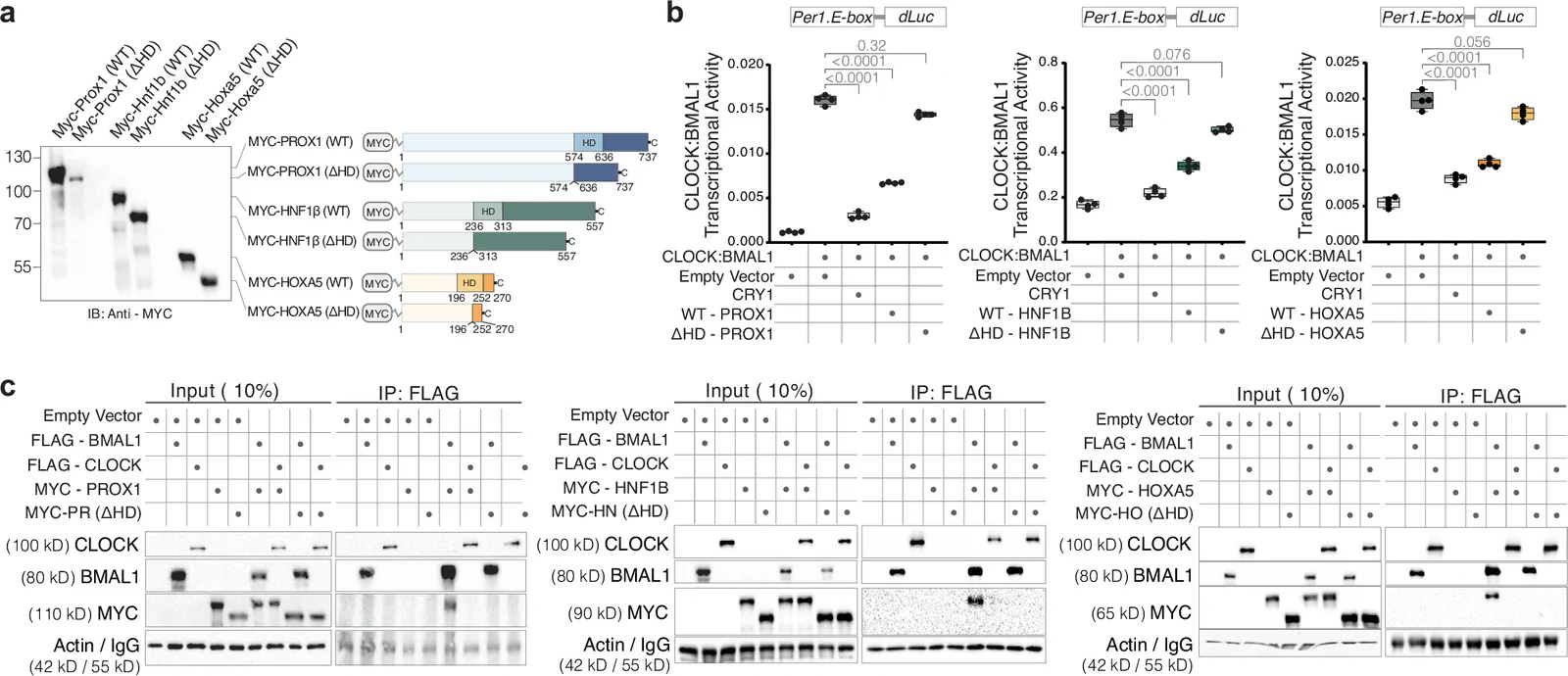
PROX1, HNF1B, HOXA5 homeodomain is essential for the binding to BMAL1 and CLOCK/BMAL1 transcriptional repression. **a**, Immunoblot of MYC-tagged PROX1, HNF1B and HOXA5 wild-type and homeodomain deleted mutants (ΔHD) transiently transfected into HEK293T cells. On the right, a schematic representation of wild-type and ΔHD PROX1, HNF1B, and HOXA5 proteins. **b**, Effect of the wild-type and ΔHD PROX1, HNF1B, HOXA5 proteins on CLOCK/BMAL1 transactivation. Box plots show the median, 25th and 75th percentiles (box), and minimum to maximum values (whiskers). Individual data points (n = 4 biologically independent samples) of mean firefly luciferase activity normalized to Renilla luciferase are displayed with *Padj* values from a one-way ANOVA. The CRY1 experimental condition was used as a positive control for repression. **c**, Immunoblots showing co-immunoprecipitation of transiently expressed FLAG-tagged BMAL1 and CLOCK with MYC-tagged PROX1, HNF1B, HOXA5 wild-type and ΔHD mutants in HEK293T cells. ACTIN and IgG were used as loading control for input and IP, respectively, in the same blot. Source data

We then used AlphaFold 3 to gain structural insight into the interactions of PROX1, HNF1B and HOXA5 with CLOCK/BMAL1^43^. AlphaFold 3 predictions yielded remarkable analogous structures for the complex with each homeodomain TFs. Notably, the structure prediction placed the homeodomain of each factor at the interface with the PAS domain core of the CLOCK/BMAL1 heterodimer while sandwiching the C-terminal TAD of BMAL1 (Fig. 4g and Extended Data Fig. 5a–c). To experimentally validate the prediction, we tested the effect of mutating the common BMAL1 TAD residues (E614A/L619A) placed at the interface for all predicted structures, on PROX1, HNF1B and HOXA5 transcriptional repression (Fig. 4g,h). While the double mutation (E614A/L619A) did not affect baseline CLOCK/BMAL1 transcriptional activity, it significantly attenuated the repression exerted by PROX1, HNF1B, HOXA5 and the canonical repressor CRY1 (Fig. 4i). This was consistent with previous studies showing that the TAD is essential for CRY1-mediated repression^24^, suggesting a similar mechanism for the homeodomain TFs. Co-IP revealed that BMAL1 mutations (E614A/L619A) did not affect its interaction with the homeodomain TFs (Fig. 4j), indicating that, while the TAD residues are required for repression, they are not essential for physical interaction. By contrast, deletion of the homeodomain from PROX1, HNF1B or HOXA5 abolished BMAL1 interaction and, consequently, their repressive effect (Extended Data Fig. 6a–c). Collectively, our data support a model in which the homeodomains of PROX1, HNF1B and HOXA5 interact with the CLOCK/BMAL1 complex via the PAS core and the TAD of BMAL1, enabling tissue-specific repression of gene expression as a potential mechanism of circadian transcription regulation.

### Homeodomain TFs tune organ circadian outputs

**Fig. 5:**
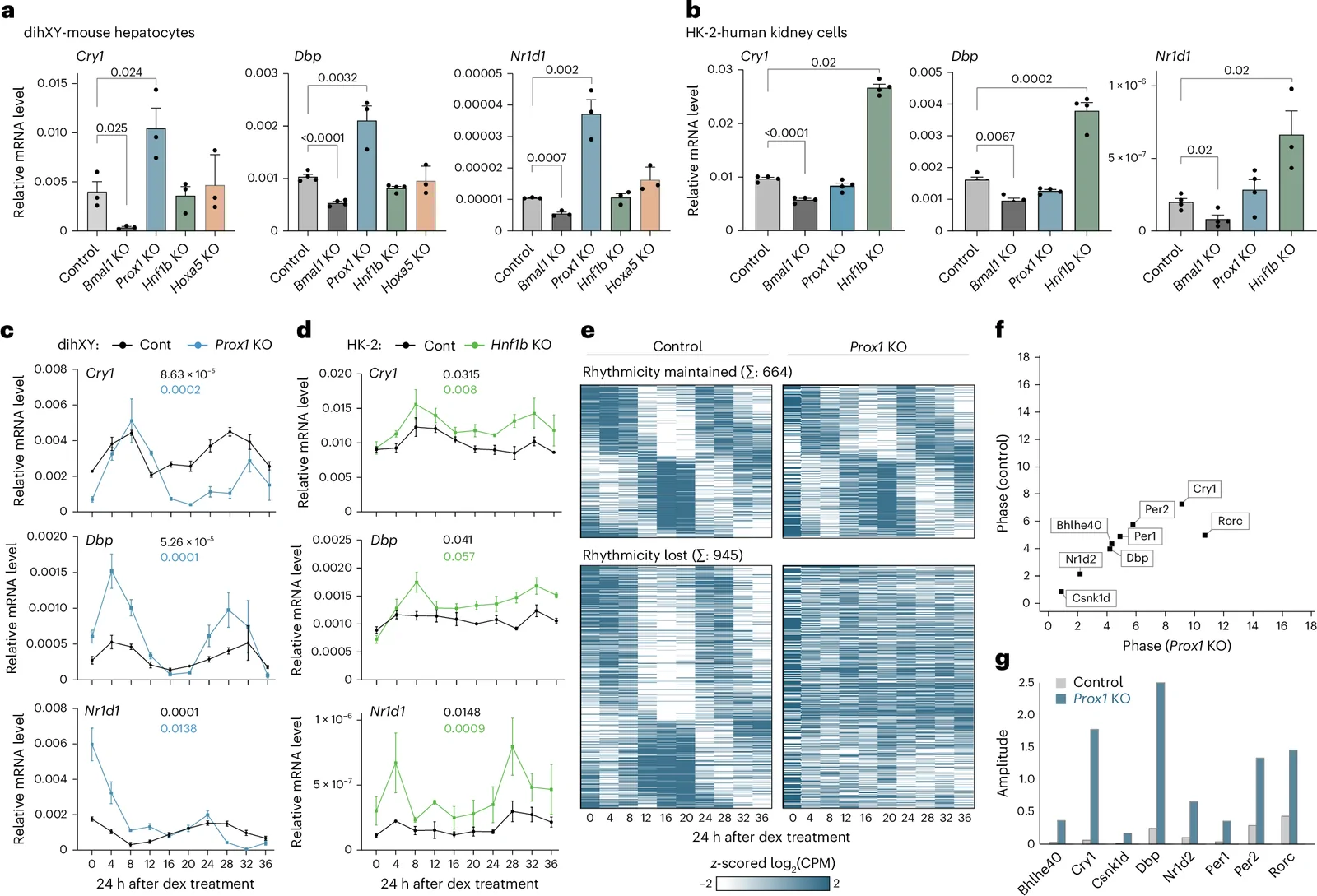
PROX1 and HNF1B are tissue-specific regulators of circadian gene expression. **a**, Steady-state RT–qPCR mRNA levels in the control and CRISPR–Cas9-mediated *Bmal1*, *Prox1*, *Hnf1b* and *Hoxa5*-KO mouse hepatocytes dihXY. Bars display mean values (± s.d., *n* = 4 biologically independent samples) normalized to control *Rplp0* mRNA levels. Two-tailed *t*-test; numbers denote *P*_adj_ values. **b**, As in **a** but for KO of *BMAL1*, *PROX1*, *HNF1b* and *HOXA5* in human kidney cells HK-2. Bars display mean values (± s.d., *n* = 4 biologically independent samples) normalized to control *Rplp0*. **c**, Temporal abundance of the indicated RT–qPCR mRNA levels in control (Cont) and *Prox1*-KO dihXY cells following dexamethasone (dex) synchronization. Data represent mean ± s.e.m. (*n* = 3 biologically independent samples) normalized to non-oscillating *Rplp0* transcript levels. **d**, As in **c**, but in *HNF1B*-KO and control HK-2 cells. *q*-values from Perseus periodicity analysis are indicated for control (black), *Prox1* KO (blue) and *HNF1B* KO (green). **e**, Heat map of rhythmic (DryR, *P* < 0.05) transcripts in control dihXY cells (left) and their corresponding values in *Prox1*-KO dihXY cells (right) across 36 h following Dex synchronization. Colours indicate RNA-seq *z*-scored log_2_(CPM) mean-timepoint replicate values (*n* = 3 biologically independent samples). Top and bottom boxes contain transcripts that maintained or lost rhythmicity (on the basis of DryR analysis), respectively, in *Prox1*-KO dihXY cells. **f**, Scatter plot showing the DryR-calculated oscillation phase for clock genes in control (*y* axis) and *Prox1*-KO (*x* axis) dihXY cells. **g**, Bar plots represent the DryR-calculated amplitude values of the rhythmic core clock genes in control and *Prox1*-KO dihXY cells. Source data

**Extended Data Fig. 7:**
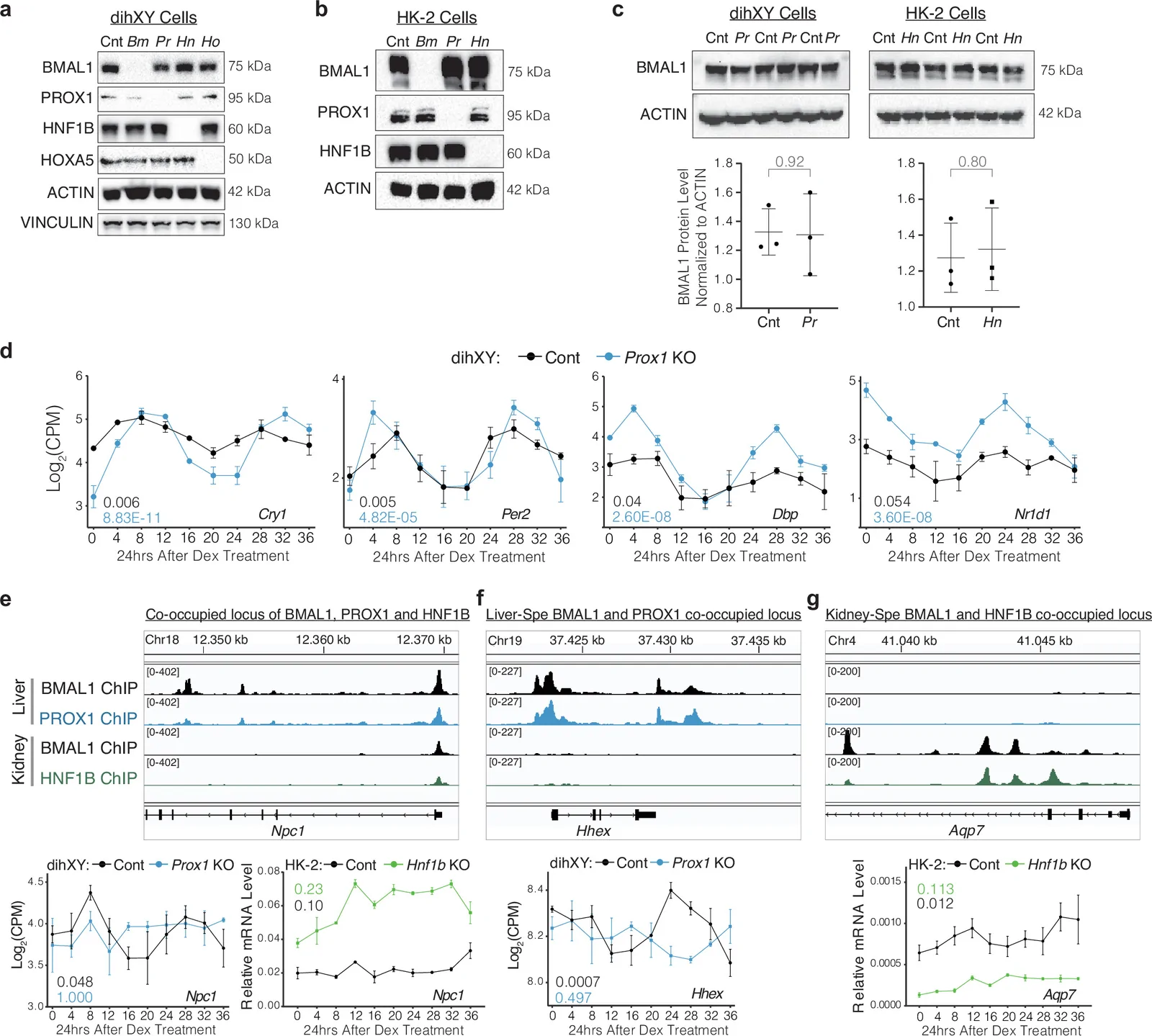
PROX1 and HNF1B modulate common and tissue - specific BMAL1 target genes in liver and kidney. **a**, **b**, Immunoblots showing endogenous protein levels of BMAL1, PROX1, HNF1B and HOXA5 in Control (Cnt) and CRISPR/Cas9-mediated individual *Bmal1* (Bm), *Prox1* (Pr), *Hnf1b* (Hn) and *Hoxa5* (Ho) knockout mouse hepatocytes, dihXY (left); Control (Cnt) and CRISPR/Cas9-mediated individual *BMAL1* (Bm), *PROX1* (Pr) and *HNF1B* (Hn) knockout human HK-2 kidney cells (right). ACTIN and VINCULIN were used as a loading control in the same blot. **c**, Immunoblots showing BMAL1 protein levels in control and *Prox1* KO dihXY cells (left) and control and *Hnf1b* KO HK-2 cells (right), with ACTIN shown as a loading control in the same blot. Bar plots show quantification of BMAL1 levels normalized to ACTIN, presented as mean ± s.d. (n = 3), with two-tailed Student’s t-test, ns: no significant. **d**, *Cry1, Per2, Dbp* and *Nr1d1* transcript levels in Control (Cont) and *Prox1* KO dihXY cells across 36hrs following dexamethasone synchronization, analyzed by RNA-seq. Values are mean ± s.d. (n = 3) of log_2_ (CPM). **e**, (Upper) IGV genome browser view of BMAL1 (black), PROX1 (blue) and HNF1B (green) occupancy at the *Npc1* locus, a common co-occupied genomic locus by BMAL1 and PROX1 in liver as well as BMAL1 and HNF1B in kidney. (Lower) *Npc1* transcript levels across 36hrs in dexamethasone-synchronized Control (Cont) and *Prox1* KO dihXY cells assessed by RNAseq, values are mean ± s.d. (n = 3) of log_2_ (CPM); and in Control (Cont) and *HNF1B* KO HK-2 cells assessed by RT-PCR, data as mean ± s.d. (n = 3) normalized to non-oscillating *Rplp0* expression levels. **f**, As in **e** but for the liver specific BMAL1 and PROX1 co-occupied *Hhex* gene. **g**, As in **e** but for the kidney specific BMAL1 and HNF1B co-occupied *Aqp7* gene. q-values from JTK analysis (dihXY) and Perseus periodicity analysis (HK-2) are indicated for control (black), *Prox1* KO (blue) and *Hnf1b* KO (green) dihXY cells (**d-g**). Source data

**Extended Data Fig. 8:**
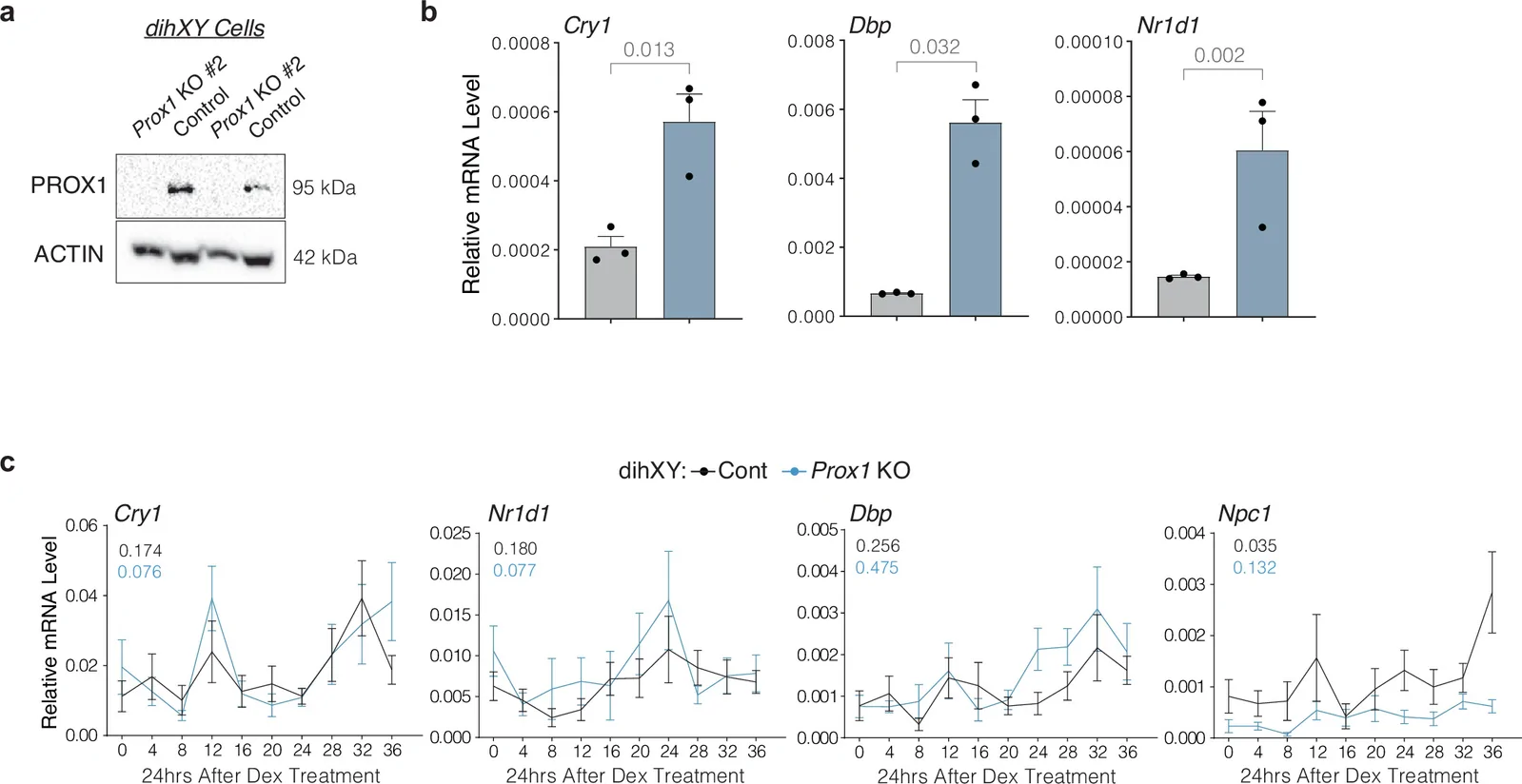
Effect reproducibility in second *Prox1* knockout dihXY clones. **a**, Immunoblots of endogenous PROX1 protein levels in a second clone for Control and CRISPR/Cas9-mediated *Prox1* knockout (KO) dihXY cell line generated with a different guide RNA (gRNA#2). ACTIN was used as a loading control in the same blot. **b**, Steady-state mRNA levels in Control (Cont) and *Prox1* knockout (KO) #2 dihXY cells analyzed by RT-qPCR. Data is represented as mean values ± s.d (n = 4 biologically independent samples) normalized to *Rplp0* expression levels as non-oscillating control mRNA and corresponding *Padj* values from a two-tailed t-test. **c**, Temporal abundance profiles (36 h after dexamethasone synchronization) for the indicated mRNAs in Control (Cont) and *Prox1* knockout (KO) dihXY #2 cells analyzed by RT-qPCR. Values are displayed as mean ± s.e.m (n = 3 biologically independent samples) normalized to non-oscillating *Rplp0* expression levels. Source data

**Extended Data Fig. 9:**
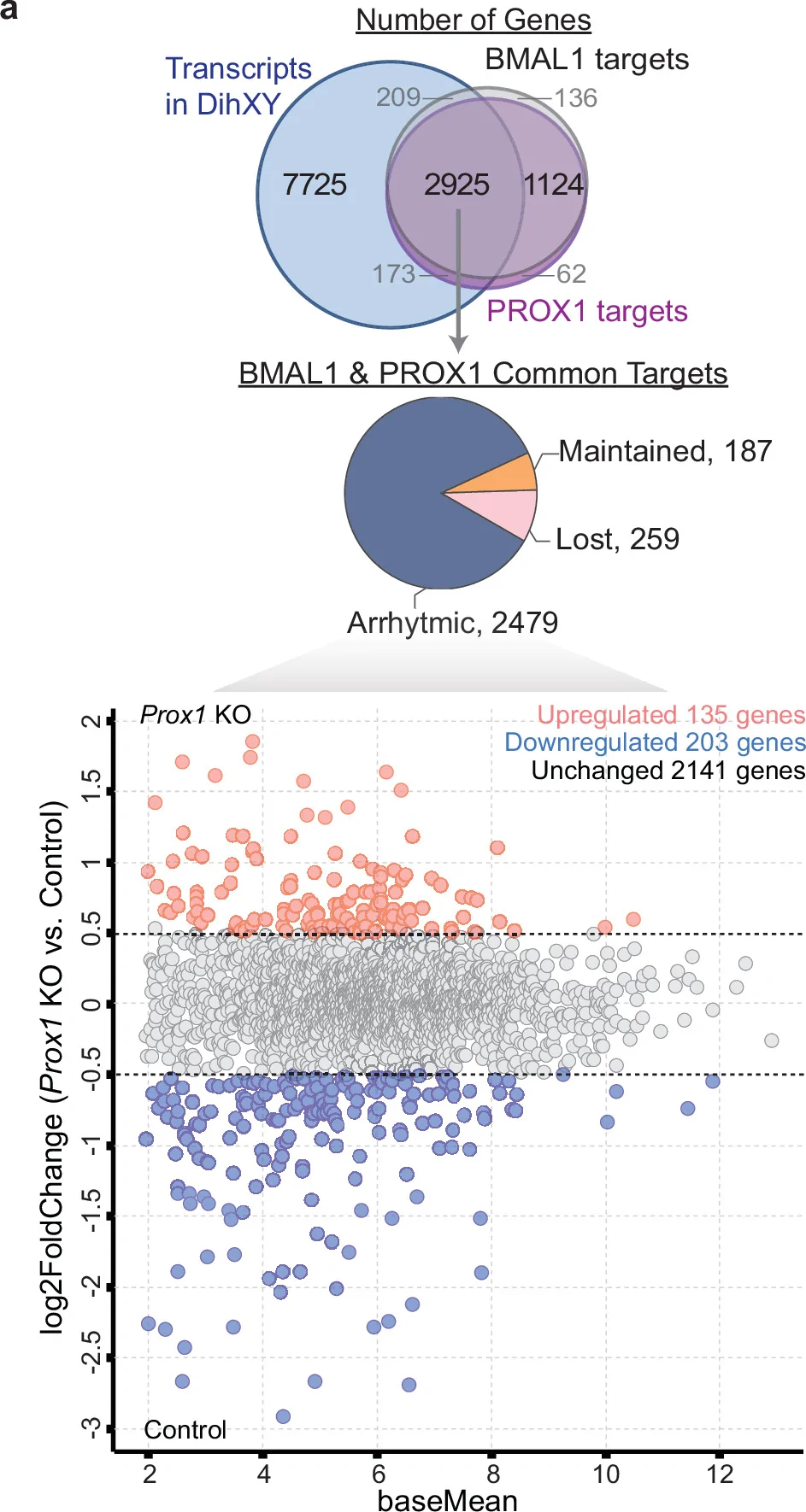
*Prox1* deletion predominantly affects the expression of rhythmic BMAL1/PROX1-associated genes. **a**, Venn diagram representing the overlap between BMAL1 and PROX1 genomic sites in mouse liver at ZT7 and all transcripts from our RNA-seq time series in dihXY cells (upper panel). Pie chart showing the DryR rhythmicity classification of common genes when comparing wildtype and *Prox1* KO RNA-seq time series (middle panel). MA plot displaying log2 fold changes between wildtype and *Prox1* KO dihXY cells as a function of base mean expression for genes that are arrhythmic in wildtype dihXY cells. Differential expression analysis was performed with DESeq2; P values were adjusted using the Benjamini–Hochberg method and genes with log2 fold > 0.05 were defined as upregulated and log2 fold < -0.05 as downregulated (lower panel). Source data

To investigate whether transcriptional coregulation by the homeodomain TFs contributes to tissue-specific circadian gene expression, we generated *Prox1*, *Hnf1b* and *Hoxa5* knockouts (KO) in a mouse hepatocyte cell line (dihXY)^44^ (Extended Data Fig. 7a). Given the interaction of PROX1 with CLOCK/BMAL1 in the liver, we hypothesized that *Prox1* deletion, but not *Hnf1b* or *Hoxa5*, affects circadian gene expression in hepatocytes. In fact, steady-state mRNA levels of core clock genes *Cry1*, *Dbp* and *Nr1d1*, whose genomic sites were co-occupied by BMAL1 and the homeodomain factors in vivo (Fig. 4d), were significantly elevated in *Prox1*-KO cells but unchanged in *Hnf1b*- and *Hoxa5*-KO counterparts. Deletion of *Bmal1* led to a dramatic reduction in those mRNAs, as expected (Fig. 5a). Applying the same rationale to the kidney, we evaluated the impact of *HNF1B* and *PROX1* deletion on the expression of core clock genes in a human kidney epithelial cell line (HK-2) (Extended Data Fig. 7b). In line with our hypothesis, we observed that steady-state transcript levels of *Cry1*, *Dbp* and *Nr1d1* were significantly increased in *HNF1B*-KO HK-2 cells but unaffected in *PROX1*-KO counterparts (Fig. 5b). These effects were not due to alterations in BMAL1 protein levels caused by the deletion of *PROX1* or *HNF1B* (Extended Data Fig. 7c). Together, these results support our hypothesis of tissue-specific modulation of CLOCK/BMAL1-driven transcription by homeodomain TFs via transcriptional repression and further show that this mechanism is not restricted to mouse tissues or cells but it is instead conserved across species. Next, we assessed temporal changes in core clock gene expression by performing reverse transcription (RT)–qPCR on dexamethasone-synchronized *Prox1*-KO dihXY and *HNF1B*-KO HK-2 cells. Although *Prox1*-KO dihXY and *HNF1B*-KO HK-2 cells displayed elevated *Cry1*, *Dbp* and *Nr1d1* mRNA levels compared with wild-type (WT) control, the oscillatory patterns and expression phase remained unchanged (Fig. 5c,d). A confirmation of these results was done in an independent *Prox1*-KO dihXY clone generated with a different *Prox1*-targeting guide RNA (Extended Data Fig. 8a–c). Together, these findings indicate that PROX1 and HNF1B modulate the amplitude—but not the timing—of core clock genes in a tissue-specific manner. As BMAL1 and the homeodomain TFs co-occupy a broad set of genomic sites beyond core clock genes, we next investigated the impact of *Prox1* deletion on global circadian transcription in hepatocytes. To this end, we performed RNA-sequencing (RNA-seq) in dexamethasone-synchronized control and *Prox1*-deleted dihXY cells. Notably, around 60% of oscillating transcripts in control cells lost their rhythmicity^45^ in the absence of *Prox1*, indicating a widespread role for PROX1 in maintaining circadian transcriptional outputs in hepatocytes (Fig. 5e) Consistent with this broad effect, among all genes expressed in diHXY cells, 2,925 overlapped with BMAL1/PROX1 ChIP–seq peaks identified in the mouse liver at ZT7. DryR analysis classified 2,479 of these genes as arrhythmic and 446 genes (~15%) as rhythmic in WT cells. This proportion is in good agreement with previous reports in the mouse liver, estimating that only ~24% of BMAL1-bound genes are rhythmic^12^, particularly given that our analysis compares liver ChIP–seq data with RNA-seq from a hepatocyte cell line. Importantly, among the rhythmic BMAL1/PROX1-associated genes, a substantial fraction, 259 genes (~58%), lost oscillation after *Prox1* deletion, a proportion comparable to that observed when all expressed genes were considered. By contrast, *Prox1* deletion had only a modest effect on non-rhythmic BMAL1/PROX1-associated genes: 2,141 of the 2,479 arrhythmic genes (~86%) showed no change in expression, whereas only small fractions were upregulated (~5%) or downregulated (~8%) (Extended Data Fig. 9). Notably, the small fraction of transcripts that retained rhythmicity included core clock genes, which exhibited increased amplitude with unaffected phases (Fig. 5e–g, Extended Data Fig. 7d and Supplementary Table 7), as observed by RT–qPCR. Loss of rhythmicity after *Prox1* deletion in dihXY cells was particularly evident for genes whose genomic loci are co-occupied by BMAL1 and PROX1 in the liver, including sites BMAL1-PROX1 exclusive and shared with HNF1B in the kidney. As expected, rhythmic expression of genes with shared HNF1B sites in the kidney was also lost in *HNF1B-*KO HK-2 cells. A similar loss of rhythmic expression in those cells was observed for genes uniquely co-occupied by BMAL1 and HNF1B in the kidney (Extended Data Fig. 7e–g). Collectively, these results demonstrate that PROX1 and HNF1B modulate BMAL1/CLOCK-driven gene expression in a tissue-specific manner.

## Discussion

Transcriptional specificity emerges not only from TFs binding to genomic sites but also from dynamic, chromatin-associated interactions with coregulators. Here, we systematically mapped the protein interaction landscape of the core circadian CLOCK/BMAL1 heterodimer across the transcriptionally active phase in three mouse tissues using an in vivo, chromatin-resolved proteomics approach. Our atlas substantially extends earlier interaction proteomics studies performed in cells and tissues^16–18^ and uncovers numerous previously unrecognized, time- and tissue-specific interactors, revealing that CLOCK/BMAL1 complex composition is extensively remodelled across circadian time and tissue context.

Temporally, the largest number of interactors was detected at ZT7, coinciding with the reported peak of CLOCK/BMAL1 DNA occupancy in the liver^10,26^ and consistent with oscillations in chromatin accessibility and epigenetic marks at circadian enhancers^3,10^. Notably, a similar pattern was observed in the kidney and lung, where ZT7 also showed the largest number of interactions, supporting the idea that this timepoint represents a conserved phase of maximal CLOCK/BMAL1 chromatin engagement across peripheral tissues. More broadly, our data suggest a progressive temporal remodelling of CLOCK/BMAL1-associated complexes: from a relatively small complex at ZT1 enriched in canonical CRY/PER repressor constituents, to a much larger complex at ZT7 containing numerous TFs and cofactors concurring with the of peak transcriptional activity and finally to a ZT10 complex enriched for proteins involved in PTMs, including ubiquitin-dependent enzymes linked to protein stability. This temporal progression provides a plausible mechanistic framework for the modulation of CLOCK/BMAL1 activity across the transcriptional active phase. For example, the rhythmic and potentially competitive in vivo recruitment of canonical cofactors such as EP300 and CRY1^24^, together with ubiquitin-related pathways at ZT10 that may help explain the previously described role of proteasome-mediated modulation of CLOCK/BMAL1 transcriptional activity^46^.

While time modulates the interactome, tissue specificity emerges as the dominant determinant of CLOCK/BMAL1 complex composition, even though CLOCK and BMAL1 protein nuclear levels were comparable across tissues. Approximately two-thirds of all interactors were unique to a single tissue from which only around 20% were more abundant in the nuclear compartment of that tissue, indicating that differences in protein abundance alone do not predict interaction specificity, as recently reported in other contexts^1^. How CLOCK/BMAL1 interacts with distinct nuclear proteins, commonly present in all tissues, remains unknown, but PTMs, such as phosphorylation, which exhibit robust oscillations in liver nuclear proteins, probably contribute to it^8^. The fact that the proportion of organ-specific interactors correlates with the extension of the circadian transcription output supports a role for tissue-specific chromatin environments that shape CLOCK/BMAL1 DNA occupancy and thus transcriptional programmes^2,3,10,11,27^. Alternatively, distinct, perhaps locus-specific, interactors could modulate CLOCK/BMAL1 transcription without affecting its DNA binding, explaining the weak correlation between CLOCK/BMAL1 DNA binding intensity and target gene expression^12^. Benzonase treatment substantially reduced the number of interactors, confirming that a large part of the CLOCK/BMAL1 interactions were dependent on nucleic acids, predominantly associated with chromatin. However, approximately half of the transcriptional factors and cofactors resisted benzonase digestion, suggesting direct protein–protein contacts with CLOCK/BMAL1 that are not dependent on DNA.

Collectively, TFs represented 5–10% of the total common and tissue-specific interactors. Among the latter, we found three members of the homeodomain-containing TF class known for their role in the development of the tissue with which they are associated: PROX1 (liver), HNF1B (kidney) and HOXA5 (lung). PROX1 has been implicated in the regulation of glucose, lipid and bile acid metabolism in hepatocytes and the liver through interaction with nuclear receptors^35,36,39,40,47^. Given that these physiological processes are under circadian control, PROX1 function in the liver could also be mediated by its interaction with CLOCK/BMAL1, whereas there is no prior evidence of HNF1B and HOXA5 interacting with the positive arm of the circadian clock. Supporting our in vivo interaction data, AlphaFold-based predictions yielded highly similar complex structure for each homeodomain TF bound to CLOCK/BMAL1. In all cases, the predictions place an analogous interface between the homeodomain and the PAS core of the heterodimer as well as the C-terminal TAD of BMAL1. Our functional validation assays confirmed these computational predictions, showing that PROX1, HNF1B and HOXA5 homeodomains are essential for the direct interaction with CLOCK/BMAL1. Furthermore, mutation of two residues at the predicted interphase within the BMAL1 TAD (E614A/L619A) strongly impaired the repression by all three factors. This effect mirrors the abrogation of CRY1-mediated repression and EP300 co-activation observed with other BMAL1 TAD mutants and truncations^24^. Our findings thus support a shared repression mechanism in which homeodomain factors and canonical clock repressors/activators converge on the BMAL1 TAD to modulate CLOCK/BMAL1 activity, with tissue specificity conferred by co-occupancy at organ-specific loci. This is consistent with our observation that PROX1 and HNF1B co-occupy 80–90% of BMAL1 binding sites in their respective tissue, including core clock gene loci. Notably, other homeodomain TFs have been reported to occupy similar BMAL1-bound genomic regions in tissues where they play key developmental roles, although direct interactions with CLOCK/BMAL1 have not been demonstrated. Deletion of those factors in adult mice leads to aberrant circadian transcriptional outputs with little effect on core clock genes^48–51^. These findings align with our transcriptomics data showing that deletion of *Prox1* in hepatocytes and *HNF1B* in human kidney cells disrupted rhythmic lineage-specific transcription without affecting core clock gene oscillations.

Taken together, our findings support a model in which homeodomain factors, through direct interaction with CLOCK/BMAL1: (1) shape tissue-specific circadian transcription and (2) fine-tune core-clock transcriptional amplitude. In this context, it is tempting to speculate that if CLOCK/BMAL1 acts as kamikaze TFs, as previously suggested^46^, transcription repression by homeodomain TFs may represent a mechanism to buffer CLOCK/BMAL1 high transcriptional bursts. In this framework, repression via the BMAL1 TAD could offer an efficient and simple mechanism for amplitude regulation and environmental entrainment.

Overall, our interaction atlas shows that the conserved positive arm of the circadian clock crosstalk with temporally and spatially gated chromatin-associated interactors, including TFs and cofactors, which shape both core and tissue-specific circadian transcription. Among them, homeodomain TFs, associated with organ development, emerge as key modulators of tissue-specific circadian output, potentially coupling the clock to organ identity. More broadly, our findings demonstrate that tissue-specific protein interactions provide a general mechanism by which ubiquitous TFs achieve context-dependent activity. This spatiotemporal interactome atlas offers both a resource for circadian biology and a conceptual framework for understanding how combinatorial protein networks orchestrate in vivo tissue-specific gene regulation.

## Methods

### Animals and tissue collection

Male and female C57BL/6N mice (8–10 weeks of age) were maintained in individually ventilated cages under a 12-h light–dark cycle and provided with sterile food and water under specific-pathogen-free conditions. Four mice were collected at each timepoint; no statistical methods were used to predetermine sample sizes. Organs were collected from WT animals originating from the institutional breeding colony. Animal housing, breeding and use were conducted in accordance with applicable European and national animal welfare regulations. The animal facility is registered for the breeding and use of animals for scientific purposes (KVR-I/221-TA166/22_03-06). No procedures requiring separate animal experimentation approval were performed for this study.

### Antibodies

For immunoblotting, co-IP, ChIP–MS, ChIP–qPCR and ChIP–seq experiments, the following antibodies were used: anti-Bmal1 (CellSignaling, D2L7G, #14020), anti-Clock (CellSignaling, D45B10 - #5157), anti-IgG (CellSignaling, #2729), anti-Prox1 (ProteinTech, #11067-2-AP), anti-Hnf1b (NovusBio, #NBP1-89680), anti-Hoxa5 (Abcam, EPR2825(2), #ab140636), anti-Myc (CellSignaling, 9B11, #2276), anti-Actin (SantaCruz, #sc-47778) and anti-Vinculin (Sigma-Aldrich, #V9264). For immunoblotting, anti-rabbit IgG, horseradish peroxidase (HRP)-linked antibody (CellSignaling, #7074) and anti-mouse IgG, HRP-linked antibody (CellSignaling, #7076) were used as a secondary antibody.

### Plasmids

pcDNA3.Flag-Bmal1, pcDNA3.Flag-Clock and pcDNA3-Cry1 expression vectors were a gift from Charles Weitz^18^. pCMV-SPORT6-Prox1 (Horizon Discovery, #MMM1013-202770532), FR_HNF1B (Addgene, #31101) and pINDUCER-21-HOXA5 (Addgene, #97040) encoding the full length of PROX1, Myc-HNF1B and HOXA5, respectively, were purchased.

E614A and L619A mutations on pcDNA3.Flag-Bmal1 was generated using the Q5 Site-Directed Mutagenesis Kit (NEB, #E0554) using BMAL1_Mut_For and BMAL1_Mut_Rev primers.

pCMV-SPORT6-Myc-Prox1 was created by cloning the PCR-amplified ‘5x*Myc*’ sequence from FR_HNF1B using Myc_Kpni_For and Myc_Sali_Rev primers using Q5 Hot Start High-Fidelity 2X Master Mix (Q5 Mix, NEB, M0494) and inserting it into the *Kpni* and *Sali* sites of the pCMV-SPORT6-Prox1 plasmid (N terminal of *Prox1*). For the deletion of the homeodomain of *Prox1*, pCMV-SPORT6-Myc-Prox1 plasmid was amplified with Prox1_Del1_For and Prox1_Del1_Rev primers using Q5 Mix. After the phosphorylation and ligation of the PCR product, pCMV-SPORT6-Myc-Prox1.HD plasmid encoding homeodomain-deleted (576aa–635aa) Myc-tagged PROX1 was obtained.

Homeodomain deletion of HNF1B was generated as follows: FR_HNF1B plasmid was amplified with Hnf1b_HDdel_For and Hnf1b_HDdel_Rev primers using Q5 Mix. FR_HNF1B.HD plasmid encoding homeodomain-deleted (236aa–313aa) Myc-tagged HNF1B was created.

pCMV-SPORT6-Myc-Hoxa5 plasmid was generated as follows: *Hoxa5* gene was amplified from pINDUCER-21-HOXA5 plasmid with Hoxa5_KpnI_For and Hoxa5_NotI_Rev primers using Q5 Mix and inserted into the *Kpni* and *Noti* sites of pCMV-SPORT6-Myc-Prox1 plasmid after removing *Prox1* by cutting the plasmid with *Kpni* and *Noti* enzymes. Homeodomain-deleted Hoxa5 was generated from pCMV-SPORT6-Myc-Hoxa5 by amplifying the entire plasmid with Hoxa5_HomeoDel_For and Hoxa5_HomeoDel_Rev primers using Q5 Mix. After the phosphorylation and ligation of the PCR product, pCMV-SPORT6-Myc-Hoxa5.HD plasmid encoding homeodomain-deleted (196aa–252aa) Myc-tagged HOXA5 was obtained. The homeodomain positions of *Prox1, Hnf1b* and *Hoxa5* genes were defined according to Uniprot.

BMAL1_Mut_For CGGGGGCCGGTGGCCCCGTT

BMAL1_Mut_RevCATCTGCGGCCAAGAGGCTCATG

Myc_Kpni_For CAAggtaccGGATCGATTTAAAGCTATGG

Myc_Sali_Rev AAAgtcgacGTCGCCCAAGCTCTCCATTTC

Prox1_Del1_For AAGTATGCGCGTCAAGCCAT

Prox1_Del1_Rev TTCCTGCATTGCGCTTCCTG

Hnf1b_HDdel_For CTTCTTGTTGGTGGGCTCAG

Hnf1b_HDdel_Rev AAGCTGGCCATGGACGCCTA

Hoxa5_KpnI_For CAAgtcgacGGCTCCACCATGAGCTCTTA

Hoxa5_NotI_Rev AAAgcggccgcTGGGTCCTAGGGACGGAAGG

Hoxa5_HomeoDel_For CTGAAAAGCATGAGCATGGC

Hoxa5_HomeoDel_Rev GCCGCCTATGTTGTCATGACT

### Cell culture

DihXY, a mouse hepatocellular carcinoma cell line, was a gift from Steve A. Kay from the Keck School of Medicine, University of Southern California, Los Angeles. Human proximal tubular epithelial cell line (HK-2) and Human embryonic kidney cells (HEK) 293T were a gift from Dr. Maciej Lech Medizinische Klinik und Poliklinik IV, LMU Munich. dihXY, HK-2 and HEK293T cells were maintained in Dulbecco’s Modified Eagle Medium (4.5 g L^−1^ glucose, Gibco) supplemented with 10% fetal bovine serum (FBS, Gibco) and antibiotics (penicillin–streptomycin, Gibco). Cells were grown and incubated in a humidified incubator containing 5% CO_2_ at 37 °C.

Time-course experiments were conducted by treating confluent cells with dexamethasone (Dex, 1 µM) for 1 h. Afterwards, the medium was changed to a fresh, complete medium without Dex. Cells were collected after 24 h of incubation.

### Luciferase reporter assay (transactivation assay)

HEK293T cells were cotransfected using various plasmids using Lipofectamine 2000 (Thermo Fisher Scientific, #11668027) according to the manufacturer’s instructions. A total of 22 ng pGL3-Per1E54bp plasmid (Promega) per well was used to see CLOCK/BMAL1 transactivation function using its firefly luciferase gene expression driven by *Per1.E-Boxes*. A total of 15 ng of pRL-CMV plasmid (Promega) with Renilla luciferase expression driven by CMV promoter was used as an internal control to normalize transfection efficiency. The total amount of plasmid DNA was equalized with the empty pcDNA3 plasmid (Invitrogen) across all conditions. Dual luciferase activity was measured with a microplate luminometer (GloMax Navigator) using a Dual-Luciferase Reporter Assay System (Promega, #E1910). Firefly luciferase signal was divided by Renilla luciferase signal for the analysis.

### Co-IP

HEK293T cells were seeded into six-well plates, and plasmids overexpressing Flag-tagged BMAL1, mutant BMAL1(E614A/L619A), CLOCK, full-length or homeodomain-deleted versions of Myc-tagged PROX1, HNF1B or HOXA5 were separately transfected into different wells of the plate using Lipofectamine 2000 (Thermo Fisher Scientific, 11668027). Following 36 h of incubation, cells were washed and collected into IP lysis buffer (50 mM Tris–HCl (pH 8.0), 150 mM NaCl, 1% Triton X-100, 1 mM dithiothreitol (DTT) and protease inhibitors (Roche, #04693116001)). After the 30-min incubation on ice, samples were centrifuged at 21,000*g* for 10 min at 4 °C and the supernatant was collected. An equal volume from each sample was mixed and incubated at 30 °C for 2 h with agitation (1,350 rpm). Flag-tagged BMAL1 and Flag-tagged CLOCK proteins were collected from mixtures by incubating 20 µl Anti-FLAG M2 Affinity Gel (Sigma-Aldrich, #A2220) for 2 h at 4 °C on a roller shaker. Beads were then washed three times with cold TBS (pH 7.4) and resuspended in 1× Laemmli buffer to run in SDS–polyacrylamide gel electrophoresis (PAGE).

MYC–PROX1 co-IP was performed following the procedure explained above using 1 μl anti-MYC antibody and 10 μl Magna ChIP Protein A+G Magnetic Beads (Sigma-Aldrich, #16-663) per sample.

### Generation of KO cells

Genetic deletion of *Bmal1, Prox1, Hnf1b* and *Hoxa5* genes was achieved by CRISPR–Cas9 editing^52^. Paired single guide RNAs (sgRNAs) were designed for each gene^53^. Annealed and phosphorylated sgRNAs were cloned into the px459 (Addgene plasmid ID 62988). Newly generated plasmids were transfected into dihXY and HK-2 cells using Lipofectamine 3000 (Thermo Fisher Scientific). Following antibiotic selection using puromycin (Gibco, #A1113803), single-cell-derived clones were collected and screened by western blotting for the loss of the target genes.

For dihXY, the gRNA sequences are:

*Bmal1*: forward CATCAATGAGTCGCTCCC

Reverse CGGGAGCGACTCATTGATG

*Prox1*_gRNA1: forward CGGGTTGAGAATATCATTC

Reverse GAATGATATTCTCAACCCG

*Prox1*_gRNA2: forward ACTTCTCGGACGTGAAGGTA

Reverse TACCTTCACGTCCGAGAAGT

*Hnf1b*: forward ACAAGAAGATGCGCCGCAA

Reverse TTGCGGCGCATCTTCTTGT

*Hoxa5*: forward CGGCTACGGCTACAATGGC

Reverse TGCCATTGTAGCCGTAGCCG

For HK-2 cells, the gRNA sequences are:

*Bmal1*: forward TTATCACACTACGGAGTCGA

Reverse TCGACTCCGTAGTGTGATAA

*Prox1*: forward CGTCATCATTCCGAACCCCC

Reverse GGGGGTTCGGAATGATGACG

*Hnf1b*: forward CCGCAACCGGTTCAAATGG

Reverse CCATTTGAACCGGTTGCGG

### Quantitative real-time PCR

Total RNA from collected cells was isolated using RNeasy Kit for RNA Purification (Qiagen, 74106). An equal amount of isolated RNAs from each sample was reverse transcribed into cDNA using RevertAid First Strand cDNA Synthesis Kit (Thermo Fisher Scientific, #K1622). qPCR from transcripts was performed with SYBR Green (Biorad, #1708880) using Roche Light Cycler 480. For the quantification of mRNA expression, ΔCt was calculated by subtracting Ct of the control gene (*Rplp0*) from Ct of the target gene and the expression level was assessed by 2^ΔCt^. These results were further used for the statistical analysis to determine the significant difference in the mRNA expression level of genes between experimental groups. Perseus (versions 1.6.15.0) periodicity analysis^54^ was performed with the time course RT–qPCR experiments using 2^ΔCt^ values for each dataset.

The primers for human (h) and mouse (m) sequences are:

*h/mRplp0*: forward TCTCGCCAGGCGTCCTC

Reverse CGCATCATGGTGTTCTTGCC

*mCry1*: forward CACCATCCGCTGCGTCTAT

Reverse AAATCGCCACCTGTTGATGC

*mNr1d1*: forward GCAAGGGCACAAGCAACATT

Reverse GCACTCCATAGTGGAAGCCT

*mDbp*: forward ACAGCAAGCCCAAAGAACCG

Reverse CCCACCGCCACTAACGG

*hCry1*: forward CCTCCAATGTGGGCATCAAC

Reverse TCTGCTGGTTGTCCACGAAT

*hNr1d1*: forward TGAATGGCATGGTGTTACTG

Reverse CAGGCGTGCACTCCATAGT

*hDbp*: forward AGGCAAGAAAAATCCAGGTG

Reverse GCCTCGTTGTTCTTGTACCG

### Protein extraction and western blotting

Cells were collected and lysed in SDC lysis buffer (2% SDC (v/v), 100 mM Tris–HCl, pH 8.5) for western blotting. Protein concentrations of samples were determined by using the Bicinchoninic Acid Protein Assay (BCA Protein Assay Kits, Thermo Scientific, #23225). A total of 30 µg protein from each sample was loaded into an acrylamide gel. Separated proteins were transferred onto the nitrocellulose membranes (0.45 µm, Amersham Protran) using the Biorad transfer system. Nitrocellulose membranes were blocked with 5% milk in TBS-T before incubation with 1:2,000-diluted primary and 1:5,000-diluted secondary antibodies. After overnight incubation, membranes were developed with ECL Substrates (Thermo Fisher Scientific, SuperSignal West Pico PLUS) for the appropriate time. Images were captured using the ChemidocTM XRS+ system and visualized using Image Lab Software 3.0.

### ChIP–MS

Liver, kidney and lung tissues from four individual WT mice collected at different timepoints (ZT1, ZT4, ZT7 and ZT10) were separately homogenized and simultaneously crosslinked over 10 min as follows: each 100 mg of tissue was minced using a scalpel in the presence of 1 ml crosslinking solution at room temperature (RT, 1% formaldehyde (Sigma-Aldrich, #F8775) in PBS) and homogenized by using a 7-ml glass dounce with tight pistil (B) until the solution became smooth. Homogenized tissues were rotated on the roller shaker until the 10-min incubation period was up. The crosslinking was quenched by adding 1.25 M glycine solution to a final concentration of 125 mM. After 5 min of incubation on a roller shaker at RT, the homogenized tissue was pelleted with centrifugation at 1,000*g* for 3 min at 4 °C. After washing the sample twice with ice-cold PBS, the pellet was resuspended and incubated with 1 ml cold Fast IP Buffer (150 mM NaCl, 5 mM EDTA (pH 7.5), 5 mM EDTA (pH 7.5), 1% Triton X-100, 0.5% iGepal and protease inhibitors) for 10 min on ice and then homogenized with 22-G and 25-G needles, respectively. Afterwards, samples were centrifuged at 8,000*g* for 1 min at 4 °C, supernatants were discarded and pellets were resuspended again in 1 ml Fast IP Buffer and homogenized with needles before filtering with the cell strainer (70 µm) attached to a 50-ml Falcon tube. After centrifugation, nuclear pellets were lysed with 1 ml ice-cold shearing buffer (1% SDS, 10 μM EDTA (pH 8.0), 50 mM Tris–HCl (pH 8.0) and protease inhibitors). After 10 min of incubation on ice, nuclear lysates were transferred into a polystyrene Falcon tube and sonicated (sheared) on maximum output setting, 30 cycles (on/off, 30/30 s) in Bioruptor (Diagenode) at 4 °C until chromatin was sheared to an average DNA fragment length of ±500 base pairs (bp). Sheared chromatin was then centrifuged at 21,000*g* for 10 min, and supernatants were collected. Protein concentration was determined with the BCA assay following the manufacturer’s protocol. From this, 100 µg protein was kept for the input proteome (see the ‘Sample Preparation of Input Proteome for LC–MS/MS’ section).

For each antibody-bait protein, quadruplicates of ChIP were prepared. Each ChIP was prepared as follows: 1 mg protein from fragmented DNA samples were diluted tenfold with dilution buffer (0.01% SDS, 1.1% Triton X-100, 1.2 mM EDTA (pH 8.0), 16.7 μM Tris–HCl (pH 8.0), 167 mM NaCl, protease inhibitors) and the appropriate amount of antibody was added on it. After overnight incubation of the antibody with the sample on a roller shaker at 4 °C, 20 μl Magna ChIP Protein A+G Magnetic Beads (Sigma-Aldrich, #16-663) were added and incubated on a roller shaker for 2 h at 4 °C. Beads were then washed three times with cold low-salt buffer (50 mM HEPES (pH 7.5), 140 mM NaCl, 1% Triton X-100), once with cold high-salt buffer (50 mM HEPES (pH 7.5), 500 mM NaCl, 1% Triton X-100) and once with cold TBS (50 mM Tris–HCl (pH 7.5), 150 mM NaCl), respectively, by placing tubes in a magnetic rack. After the aspiration of TBS, beads were resuspended by adding 100 μl elution buffer (2 M urea (always prepared fresh), 50 mM Tris–HCl (pH 7.5), 2 mM DTT, 10 mM chloroacetamide (CAA) and 20 μg ml^−1^ trypsin) and trypsinized overnight at 25 °C with agitation (1,350 rpm). Digested peptides were then purified and desalted with SDP-RPS StageTips.

The same ChIP–MS protocol was applied for the benzonase treatment, but the shearing and dilution buffers did not contain EDTA. Following the shearing and BCA measurement, the dilution buffer (containing an extra 1 mM MgCl), containing benzonase (Sigma-Aldrich, #E1014-25KU), was added to the fragmented DNA. Samples were incubated with benzonase for 30 min at RT on a roller shaker. Thereafter, 1.2 mM EDTA was added to the samples and the addition of antibody and dilution buffer was continued as explained above.

### Sample preparation of input proteome for LC–MS/MS

The proteome of nuclear-enriched input materials of ChIP–MS samples was prepared for each tissue (liver, kidney and lung) and timepoints ZT4, ZT7 and ZT10. A total of 100 µg protein from fragmented DNA from each sample was mixed with 100% acetone to a final concentration of 80%, to then be precipitated using acetone. After overnight incubation, proteins were collected by centrifugation at 21,000*g* for 10 min at 4 °C. After washing twice with 80% acetone, protein pellets were dried and dissolved in 200 µl SDC lysis buffer (2% (w/v) SDC, 100 mM Tris–HCl (pH 8.5)). Samples were then boiled at 95 °C for 5 min and homogenized by sonication at 4 °C (ten cycles (30/30 s) maximum output), after being cooled down. Following the measurement of protein concentrations with BCA, 10 µg protein was alkylated and reduced by adding 1:10 volume of the reduction–alkylation solution (100 mM TCEP, 400 mM 2-CAA, pH ~7.0). After 5 min of incubation at 45 °C with agitation (1,350 rpm), samples were digested with trypsin and LysC enzymes at an enzyme/protein ratio of 1:100 at 37 °C overnight with agitation (1,350 rpm).

Digested peptides were then purified and desalted in a three-layer in-house-made SDB-RPS StageTips as follows. First, StageTips^55^ were equilibrated with 100 µl solution 1 (100% acetonitrile (ACN)), solution 2 (30% methanol with 0.2% trifluoroacetic acid (TFA)) and solution 3 (0.2% TFA), respectively, with in-between centrifugation at 600*g* at RT until all liquid had passed through. For input proteome samples, digested peptides were acidified with the addition of 1:1 (v/v) volume of 100% isopropanol containing 2% TFA to a final concentration of 1% TFA and loaded into equilibrated StageTips. For ChIP–MS, digested peptides were acidified with TFA to a final concentration of 1% and loaded into equilibrated StageTips. After centrifugation at 600*g*, tips were washed twice with 100 µl isopropanol and 100 µl 0.2% TFA, respectively. Peptides were eluted by adding 60 µl elution buffer (0.625% ammonium hydroxide (NH_4_OH) in 80% ACN) and collected into clean tubes via centrifugation at 600*g* at RT. After drying eluted peptides using a SpeedVac centrifuge at 45 °C, samples were dissolved in A* Buffer (2% ACN, 0.1% TFA) and stored at −20 °C until MS measurement.

### LC–MS/MS measurement and processing

For both ChIP and proteome samples, 200 ng peptides were loaded into 50-cm, in-house packed reversed-phase columns (75-μm inner diameter, ReproSil-Pur C18-AQ 1.9-μm resin (Dr. Maisch GmbH)) kept on an oven (Sonation) at 60 °C and mounted to the EASY-nLC 1200 system (Thermo Fisher Scientific).

#### ChIP–MS data

Peptides were separated in Buffer B (80% ACN with 0.1% formic acid (FA)) over a gradient of 90 min in length (5% to 30% Buffer B for 70 min followed by 60% for 6 min, 95% for 8 min and 5% for 3 min) with a flow rate of 300 nl min^−1^. Eluted peptides were electrosprayed, via a nano-electrospray source, into an Orbitrap Exploris 480 Mass Spectrometer (Thermo Fisher Scientific) using data-dependent (DDA) mode, acquired with scanning of the top 20 method encompassing an MS1 survey scan recorded with 300–1,650 *m*/*z* range and 60,000 resolution. Raw files were processed with the MaxQuant Software (version 2.1.1.0) searching against the UniprotKB mouse database (release 2022) using default settings and with label-free quantification (LFQ) enabled (minimum ratio count of 1 and match between runs)^56^.

#### Nuclear input proteome samples

Peptides were separated in Buffer B (80% ACN with 0.1% FA) over a gradient of 120 min in length (5% to 30% Buffer B for 95 min followed by 60% for 5 min, 95% for 10 min and 5% for 5 min) with a flow rate of 300 nl min^−1^. Eluted peptides were electrosprayed, via a nano-electrospray source, into an Orbitrap Exploris 480 Mass Spectrometer (Thermo Fisher Scientific) using data-independent (DIA) mode, acquired with the following parameters: full scan range of 350–1,000 *m*/*z* at a resolution of 120,000. The AGC was set to 300% at a maximum injection time of 45 ms. Higher-energy collisional dissociation (HCN) (normalized collision energy (NCE) 30%) was used for precursor fragmentation and resulting fragment ions were recorded in 84 DIA windows at a resolution of 15,000. Resulting raw files were processed with DIA-NN 1.8.2 beta 22 using an in silico spectral mouse library enabling N-terminal methionine excision, cysteine carbamidomethylation and one missed cleavage. The peptide length range was set to 7–35 amino acids, and the precursor *m*/*z* range was 350–1,000. For the rest, default parameters were used and the match between runs was not activated.

#### BMAL1 and CLOCK ChIP–MS at ZT1 and PROX1, HNF1B and HOXA5 ChIP–MS at ZT10 samples

Peptides were separated in Buffer B (80% ACN with 0.1% FA) over a gradient of 100 min in length (5% to 30% Buffer B for 75 min followed by 60% for 5 min, 95% for 10 min and 5% for 5 min) with a flow rate of 400 nl min^−1^. Eluted peptides were electrosprayed, via a nano-electrospray source, into an Orbitrap Exploris 480 Mass Spectrometer (Thermo Fisher Scientific) using DIA mode, acquired with the following parameters: full scan range of 350–1,000 *m*/*z* at a resolution of 120,000. The AGC was set to 300% at a maximum injection time of 45 ms. HCN (NCE 30%) was used for precursor fragmentation and resulting fragment ions were recorded in 84 DIA windows at a resolution of 15,000. Resulting raw files were processed with DIA-NN 2.2.0 using an in silico spectral mouse library enabling N-terminal methionine excision, cysteine carbamidomethylation and one missed cleavage. The peptide length range was set to 7–35 amino acids, and the precursor *m*/*z* range was 350–1,000. For the rest, default parameters were used and the match between runs was not activated.

### Statistics and reproducibility

#### ChIP–MS data

Quantified ChIP–MS intensities were imported into Perseus (versions 1.6.15.0 and v1.6.10.50) for downstream analysis. Before analysis, common contaminants, reverse sequences proteins only identified by site and immunoglobulin chain isoforms were removed. LFQ intensities were log_2_-transformed.

For each tissue and ZT, statistical analysis of the bait proteins BMAL1 and CLOCK was individually conducted versus IgG control ChIP–MS data in biological quadruplicates. Proteins with fewer than three valid LFQ values in either the BMAL1, CLOCK, or IgG ChIP–MS replicates were excluded. Missing values in IgG control replicates were imputed from a left-shifted normal distribution at the lower tail of the global intensity distribution (down-shift, 2.5; width, 0.1). For statistical comparison between bait and IgG controls, Student’s two-sample *t*-test with permutation-based FDR correction (250 permutations) was performed as implemented in Perseus. An FDR of 0.01 with an *S*_0_ value of 0.5 was used for liver and kidney datasets, and an FDR of 0.05 with an *S*_0_ of 0.1 for lung datasets. This analysis yielded a list of significantly enriched proteins for each timepoint in BMAL1 ChIP–MS versus IgG (for example, BMAL1_ZT4) and CLOCK ChIP–MS versus IgG (for example, CLOCK_ZT4). Proteins significantly enriched in both BMAL1 and CLOCK were intersected to define a final set of enriched proteins for a tissue at a certain ZT (for example, List of Liver_ZT4). Proteins exclusively enriched only in BMAL1 or only in CLOCK ChIP–MS were not considered for further analysis.

PCA of lung datasets revealed several outlier replicates, which were excluded from downstream analysis: BMAL1_ZT4_rep3, CLOCK_ZT4_rep4, IgG_ZT4_rep3, BMAL1_ZT7_rep4, CLOCK_ZT7_rep2, IgG_ZT7_rep3, BMAL1_ZT10_rep1, CLOCK_ZT10_rep3 and IgG_ZT10_rep4. After removing these outliers, proteins with less than two valid LFQ values across the remaining replicates were filtered out. Finally, significantly enriched proteins from all nine datasets were integrated using their individual log_2_ fold change (FC) values for downstream analyses.

The functional enrichment analysis was performed in Perseus (v1.6.15.0) using Fisher’s exact test (*P* value <0.01) compared with an in silico mouse background gene list. Enriched Gene Ontology Biological Process (GOBP) terms were further filtered to retain entries with an enrichment factor >1 and an intersection size >5. TFs and cofactors were defined and annotated on the basis of AnimalTFDB4.0^57^.

#### Nuclear input proteome samples

Quantified nuclear proteome intensities were imported into Perseus (v1.6.15.0 and v1.6.10.50)^54^. After log_2_ transformation of protein intensities, proteins with at least four valid values were retained in any tissue, independent of timepoints. Remaining missing values were imputed from a left-shifted normal distribution at the lower end of the global intensity range (down-shift, 2.0; width, 0.1). To assess significant differences between tissues (liver, kidney and lung) independent of time, a one-way analysis of variance (ANOVA) multiple sample test with a permutation-based FDR at 0.001 was performed. Post hoc Tukey’s honestly significant difference (HSD) testing identified pairwise differences between groups (FDR of 0.05). Proteins significantly enriched in only one tissue were considered tissue exclusive.

### ChIP–seq

The ChIP–seq protocol was followed with small modifications as reported^58^. In detail, 150 mg tissue was divided into three pieces (50 mg) and placed in 2-ml tubes with 1 ml of lysis buffer (10 mM HEPES-KOH (pH 7.3), 10 mM KCl, 5 mM MgCl2, 0.5 mM DTT and protease inhibitors) and a stainless bead. Homogenized tissue lysates using a TissueLyser (30 Hz) for 2 min were filtered through a cell strainer (70 µm) and pooled into a 50-ml Falcon tube. Following centrifugation at RT, 2,000*g* for 3 min, supernatants were discarded and pellets were crosslinked by incubating with 10 ml 1% formaldehyde (in PBS) on gentle shaking for 15 min at RT. The crosslinking reaction was stopped by adding 1.5 ml 1 M glycine to the sample. Samples were incubated for an additional 5 min at RT with gentle shaking before being centrifuged at 2,000*g* for 10 min. Pellets were washed with cold PBS and resuspended in 1 ml cold Fast IP buffer (150 mM NaCl, 5 mM EDTA (pH 7.5), 5 mM Tris (pH 7.5), 1% Triton X-100, 0.5% NP40 and protease inhibitors). Samples were homogenized again and mechanically lysed by going through a syringe (24 G). After centrifugation at 13,800*g* for 1 min at 4 °C, pellets were resuspended in shearing buffer (1% SDS, 10 μM EDTA (pH 8.0), 50 mM Tris–HCl (pH 8.0) and protease inhibitors) and sheared using a Bioruptor until the average 1-kb chromatin length. After centrifugation at 13,800*g*, 4 °C for 10 min, 10% of the supernatant was saved as an input and the rest was diluted 1:10 in dilution buffer (0.01% SDS, 1.1% Triton X-100, 1.2 mM EDTA (pH 8.0), 16.7 mM Tris (pH 8.0), 0.167 M NaCl and protease inhibitors). Samples were incubated with the specified antibodies (8 μg) overnight at 4 °C in a rotating wheel. Dynabeads (M-280 Sheep Anti-Rabbit IgG, Thermo Fisher Scientific, #11203) were prewashed and blocked with 5% BSA in PBS overnight at 4 °C on a rotation wheel. Next, beads were added to the chromatin-IP solution and incubated for 6 h at 4 °C on a rotating wheel. Beads were collected using a magnetic rack and washed six times with cold Fast IP buffer. After the last wash with Tris-EDTA (TE) buffer, beads were incubated with 100 μl Beads Elution Buffer (105 μl NaHCO3 (1 M), 52.5 μl 20% (w/v) SDS and 892.5 μl H_2_O) in a thermoblock with agitation (1,000 rpm). After collecting the samples, a second elution was performed. Input material and 200 μl of the yielded samples were reverse crosslinked by incubating with 300 mM NaCl overnight at 65 °C. The next day, samples were incubated with RNase A (Thermo Fisher Scientific, #EN0531) for 30 min at 37 °C and then with proteinase K (Thermo Fisher Scientific, #AM2546) for 2 h at 56 °C. Then, chromatin was purified using the PCR purification Kit (Qiagen, #28004).

Library preparation and sequencing were conducted at BGI-Genomics in Hong Kong, China, using the DNBseq platform (project no. F23A430001964). Sequencing reads were aligned to the Mus musculus GRCm39 genome (Ensembl release 111) using Bowtie2 v2.5. The aligned chromatin fragments were converted to BED files using bamToBed (Bedtools v2.31), read into R as GRanges objects, converted to coverage and exported in bigWig format for visualization. Peak calling was performed using HOMER v4.11 with default parameters to identify enriched regions. Only peaks that overlapped with the BMAL1 binding regions defined in ref. ^11^ were considered for downstream analyses of BMAL1, PROX1 and HNF1B ChIP–MS experiments.

The functional enrichment analysis was performed by Perseus (v1.6.15.0) using Fisher’s exact test (*P* value <0.01) and compared with an in silico mouse gene list. Enriched Gene Ontology terms of Biological Process were further filtered (enrichment factor >1, intersection size >50).

### ChIP–qPCR

Chromatin IP was performed as explained until the washes of magnetic beads (see details in the ‘ChIP–MS’ section). We kept 10% of the ChIP starting material as an input. Following the last wash of beads with TBS, antibody-protein-DNA complexes were eluted by the incubation of beads within 200 µ elution buffer (1% SDS, 0.1 M NaHCO_3_ (pH 8.0)) for 15 min at 37 °C with agitation (1,350 rpm). The supernatant was transferred to another tube after the spin down of the beads. This step was repeated two more times with 150 µl elution buffer. The solution was then reverse crosslinked by incubating with 300 mM NaCl overnight at 65 °C with agitation (1,350 rpm). The following day, samples were treated with RNase A for 30 min at 37 °C and Proteinase K for 2 h at 56 °C, respectively. Then, DNA was purified with a PCR purification kit (Promega, #A6752). qPCR of purified DNAs was performed as explained (see the ‘Quantitative real-time PCR’ section). For the analysis, ΔCt was calculated by subtracting Ct (ChIP) from Ct (input), and the percentage input (% input) was assessed by 2^ΔCt^.

Primer sequences for ChIP–qPCR were defined according to ChIP–seq results:

*Cry1*: forward GCTTCTCATTGGGCGGCAT

Reverse CGCATCATGGTGTTCTTGCC

*Dbp*: forward CAAAAACCAGTGCTGCACATTC

Reverse CGCATCACGTGTGGCCTA

*Nr1d1*: forward CACCCCAACGAGGAACCC

Reverse GGGGTTTTCCTTGCGACAGA

*Efna3*: forward TAGGCTACTGCAGGTCTGTCAA

Reverse GGCTCCCACCTCTCATCACG

### AlphaFold 3 protein complex prediction

Protein complex structure predictions were carried out using AlphaFold 3 (ref. ^43^) (https://alphafoldserver.com/). For the model generations, mouse proteins CLOCK (UniProt ID: O08785), BMAL1 (UniProt ID: Q9WTL8), PROX1 (UniProt ID: P48437), HNF1B (UniProt ID: P27889) and HOXA5 (UniProt ID: P09021) were used. The best output model (ranked automatically in the pipeline 0–4 from the best to the worst according to pLDDT as a per-residue measure of local confidence) was visualized and analysed using ChimeraX^59^. The confidence of the predictions was evaluated with Predicted Aligned Error (PAE). PAE is the expected positional error at residue *X*, measured in Ångströms (Å), if the predicted and actual structures were aligned on residue *Y*.

### RNA-seq

DihXY cells (WT and Prox1 KO (*n* = 3 per timepoint)) were treated with dexamethasone and collected 24 h after the synchronization at 4-h intervals over 36 h. Cell pellets were collected and RNA was isolated using the RNeasy Kits for RNA Purification (Qiagen, 74106). RNA sequencing was performed at the Sequencing Core Unit at the TranslaTUM, Technical University Munich (TUM).

Library for bulk-sequencing of poly(A)-RNA was prepared accordingly^60^, and barcoded cDNA of each sample was generated with a Maxima RT polymerase (Thermo Fisher) using oligo-dT primer containing barcodes, unique molecular identifiers (UMIs) and an adaptor. 5′-Ends of the cDNAs were extended by a template switch oligo (TSO), and full-length cDNA was amplified with primers binding to the TSO site and the adaptor. NEB UltraII FS kit was used to fragment cDNA. After end repair and A-tailing, TruSeq adapters were ligated and 3′-end fragments were finally amplified using primers with Illumina P5 and P7 overhangs. The library was sequenced on a NextSeq1000 (Illumina) with 100 cycles for the cDNA in read 1 and 18 cycles for the barcodes and UMIs in read 2. Data were processed using the published Drop-seq pipeline (v1.0) to generate sample- and gene-wise UMI tables^61^. The reference genome (GRCm38) was used for alignment. Transcript and gene definitions were used according to GENCODE M25. Differential expression analysis was performed in R v.4.2.2 with DESeq2 v.1.38.2^62^.

For periodicity analysis and downstream data visualization, the first RNA-seq counts were normalized using counts per million (CPM) normalization to generate a matrix with log_2_-transformed values. Differential rhythmicity analysis was performed using the dryseq() function from the R package dryR^45^ using default parameters and period set to 24 h. No additional filtering was applied to the dryR output models. Statistically significant rhythmicity values were obtained for individual transcript time series data using the R package MetaCycle^63^ (v1.2.0), with cycMethod ‘JTK’, a min(per) of 21, and a max(per) of 27. Rhythmicity cutoff values were set up to *P* < 0.05.

### Reporting summary

Further information on research design is available in the Nature Portfolio Reporting Summary linked to this article.

## Online content

Any methods, additional references, Nature Portfolio reporting summaries, source data, extended data, supplementary information, acknowledgements, peer review information; details of author contributions and competing interests; and statements of data and code availability are available at https://doi.org/10.1038/s41556-026-02041-4.

## Supplementary information

Reporting Summary

Peer Review File

Supplementary Table 1

## Source data

Figs. 1–5 and Extended Data Figs. 1–9 Source data for all quantified data shown in Figs. 1–5 and Extended Data Figs. 1–9. The Excel file contains clearly named tabs corresponding to Figs. 1–5 and Extended Data Figs. 1–9.

Figs. 1–5 and Extended Data Figs. 1–9 Unprocessed immunoblots and/or gel images corresponding to Figs. 1–5 and Extended Data Figs. 1–9. The PDF contains clearly labelled sections for Figs. 1–5 and Extended Data Figs. 1–9.

## Data Availability

Proteomics data have been deposited to the ProteomeXchange Consortium via the PRIDE^64^ partner repository with the dataset identifiers PXD062751 and PXD077567. RNA-seq data are available in the European Nucleotide Archive (ENA) under accession no. PRJEB93884, and ChIP–seq data at the Gene Expression Omnibus (GEO) under accession no. GSE302237. AlphaFold 3 interaction prediction parameters can be provided during the revision process on editorial and/or review request. Source data are provided with this paper.
