# Author Correction: CLOCK/BMAL1 interactome uncovers homeodomain factors as tissue regulators

**DOI:** 10.1038/s41556-026-02092-7

**Published:** 2026-09-23

**Authors:** Fatih Aygenli, Lukas A. Huschet, Tanja Popp, Andrea Ribeiro, Darina Barkhatova, Céline Jouffe, Riccardo Trozzo, Jerome S. Menet, Roland Rad, Kenneth A. Dyar, Maciej Lech, Tobias Straub, Alicia K. Michael, Maria S. Robles

**Affiliations:** 1 Institute of Medical Psychology, LMU Medizin, Ludwig-Maximilians-Universität München, Munich, Germany; 2 Chair of Molecular Biology, Biomedical Center, LMU Medizin, Ludwig-Maximilians-Universität München, Munich, Germany; 3 School of Medicine, Klinikum Rechts Der Isar, Department of Nephrology, Technical University of Munich, Munich, Germany; 4 Institute of Science and Technology Austria, Klosterneuburg, Austria; 5 Metabolic Physiology, Institute for Diabetes and Cancer, Helmholtz Diabetes Center, Helmholtz Munich, Neuherberg, Germany; 6 German Center for Diabetes Research (DZD), Neuherberg, Germany; 7 Institute of Molecular Oncology and Functional Genomics, School of Medicine, Technische Universität München, Munich, Germany; 8 Center for Translational Cancer Research (TranslaTUM), School of Medicine, Technische Universität München, Munich, Germany; 9 German Cancer Consortium (DKTK), Heidelberg, Germany; 10 Department of Medicine II, Klinikum Rechts der Isar, School of Medicine, Technische Universität München, Munich, Germany; 11 Department of Biology, Center for Biological Clock Research, Texas A&M University, College Station, TX USA; 12 Department of Medicine IV, LMU University Hospital, LMU Medizin, Ludwig-Maximilians-Universität München, Munich, Germany; 13 Bioinformatic Unit, Biomedical Center, LMU Medizin, Ludwig-Maximilians-Universität München, Munich, Germany

**Keywords:** Circadian rhythms, Proteomics

Correction to: *Nature Cell Biology* https://doi.org/10.1038/s41556-026-02041-4, published online 6 August 2026.

In the version of the article initially published, Riccardo Trozzo’s name appeared incorrectly (as Ricardo Trozzo) and the affiliation of Maciej Lech was incorrect and should have been the “Department of Medicine IV, LMU University Hospital, LMU Medizin, Ludwig-Maximilians-Universität München, Munich, Germany”. These corrections have been made to the HTML and PDF versions of the article.

