# Supplementary material for: CLOCK/BMAL1 interactome uncovers homeodomain factors as tissue regulators
Source: Nat Cell Biol. 2026 Aug 6;28(9):1890–900. doi: 10.1038/s41556-026-02041-4 (PMC13561871; doi:10.1038/s41556-026-02041-4)
Supplement: Supplementary file 1 [file 41556_2026_2041_MOESM1_ESM.pdf]

nature portfolio

Corresponding author(s): Maria S. Robles

Last updated by author(s): 01.07.2026

Reporting Summary

Nature Portfolio wishes to improve the reproducibility of the work that we publish. This form provides structure for consistency and transparency in reporting. For further information on Nature Portfolio policies, see our Editorial Policies and the Editorial Policy Checklist.

Statistics

For all statistical analyses, confirm that the following items are present in the figure legend, table legend, main text, or Methods section.

n/a	Confirmed	
☐	☒	The exact sample size (*n*) for each experimental group/condition, given as a discrete number and unit of measurement
☐	☒	A statement on whether measurements were taken from distinct samples or whether the same sample was measured repeatedly
☐	☒	The statistical test(s) used AND whether they are one- or two-sided<br>*Only common tests should be described solely by name; describe more complex techniques in the Methods section.*
☐	☒	A description of all covariates tested
☐	☒	A description of any assumptions or corrections, such as tests of normality and adjustment for multiple comparisons
☐	☒	A full description of the statistical parameters including central tendency (e.g. means) or other basic estimates (e.g. regression coefficient) AND variation (e.g. standard deviation) or associated estimates of uncertainty (e.g. confidence intervals)
☐	☒	For null hypothesis testing, the test statistic (e.g. *F*, *t*, *r*) with confidence intervals, effect sizes, degrees of freedom and *P* value noted<br>*Give P values as exact values whenever suitable.*
☒	☐	For Bayesian analysis, information on the choice of priors and Markov chain Monte Carlo settings
☒	☐	For hierarchical and complex designs, identification of the appropriate level for tests and full reporting of outcomes
☒	☐	Estimates of effect sizes (e.g. Cohen's *d*, Pearson's *r*), indicating how they were calculated

Our web collection on statistics for biologists contains articles on many of the points above.

Software and code

Policy information about availability of computer code

Data collection	Descriptions of the software used to collect data relating to the study are included in the Methods section.
Data analysis	Detailed descriptions of the codes and software used to analyze data as part of this study are included in the Methods section. For raw data processing, analysis and representation, the following softwares and packages were used: MaxQuant v2.1.1.0, DIA-NN 1.8.2 beta 22, DIA-NN 2.2.0, Perseus v1.6.15.0 and v1.6.10.50, , GraphPad Prism 8, Adobe Illustrator 2025, Bowtie2 v2.5, Bedtools v2.31, HOMER v4.11, IGV v2.10.0, Drop-seq pipeline v1.0, R v4.2.2, DESeq2 v1.38.2, R package DryR, R package MetaCycle, Bio-Rad ImageLab v6.1.0, AlphaFold 3, ChimeraX 1.9

For manuscripts utilizing custom algorithms or software that are central to the research but not yet described in published literature, software must be made available to editors and reviewers. We strongly encourage code deposition in a community repository (e.g. GitHub). See the Nature Portfolio guidelines for submitting code & software for further information.

Data

Policy information about availability of data

All manuscripts must include a data availability statement. This statement should provide the following information, where applicable:

- Accession codes, unique identifiers, or web links for publicly available datasets
- A description of any restrictions on data availability
- For clinical datasets or third party data, please ensure that the statement adheres to our policy

The mass spectrometry proteomics data have been deposited to the ProteomeXchange Consortium via the PRIDE partner repository with the dataset identifier PXD062751 and PDX077567. During the revision phase reviewers can access the dataset by logging in to the PRIDE website using the following account details: Username: reviewer_pxd062751@ebi.ac.uk and Password: JgU6yi1XloG0 for PXD062751 and Username: reviewer_pxd077567@ebi.ac.uk and Password: ESIPMq1DeeDV for PDX077567. RNA-Seq data is available in the European Nucleotide Archive (ENA) under accession number PRJEB93884, and ChIP-Seq data at the Gene Expression Omnibus (GEO) under accession number GSE302237 (token access during review process mlkxeassfvkbzcf).

Research involving human participants, their data, or biological material

Policy information about studies with human participants or human data. See also policy information about sex, gender (identity/presentation), and sexual orientation and race, ethnicity and racism.

Reporting on sex and gender	n/a
Reporting on race, ethnicity, or other socially relevant groupings	n/a
Population characteristics	n/a
Recruitment	n/a
Ethics oversight	n/a

Note that full information on the approval of the study protocol must also be provided in the manuscript.

Field-specific reporting

Please select the one below that is the best fit for your research. If you are not sure, read the appropriate sections before making your selection.

☒ Life sciences ☐ Behavioural & social sciences ☐ Ecological, evolutionary & environmental sciences

For a reference copy of the document with all sections, see nature.com/documents/nr-reporting-summary-flat.pdf

Life sciences study design

All studies must disclose on these points even when the disclosure is negative.

Sample size	No statistical methods had been applied to determine the sample size. We used at least 3 biological replicates for all experiments except the ChIP-Seq experiment.
Data exclusions	Principal component analysis (PCA) of the lung ChIP-seq datasets identified several outlier replicates, which were excluded from downstream analyses. The excluded samples were: BMAL1_ZT4_rep3, CLOCK_ZT4_rep4, IgG_ZT4_rep3, BMAL1_ZT7_rep4, CLOCK_ZT7_rep2, IgG_ZT7_rep3, BMAL1_ZT10_rep1, CLOCK_ZT10_rep3, and IgG_ZT10_rep4. These exclusions were based on clear separation from the corresponding biological replicate clusters in PCA space and reflected technical rather than biological variability. For these time points, downstream analyses were performed using three biological replicates instead of four. No other data were excluded and no additional exclusion criteria were pre-established.
Replication	All experiments were conducted with independent biological replicates appropriate for each assay. The number of biological replicates are presented in the Figures and Methods.
Randomization	Randomization was not applicable to this study because samples were assigned to experimental groups based on predefined biological conditions (bait protein, tissues or time points) rather than by random allocation. Sample processing and sequencing were performed in balanced batches to minimize technical bias.
Blinding	Blinding was not performed because the experimental conditions (tissues) were inherently distinguishable during sample preparation and analysis. However, data processing were carried out using automated or standardized procedures that minimize investigator bias.

Reporting for specific materials, systems and methods

We require information from authors about some types of materials, experimental systems and methods used in many studies. Here, indicate whether each material, system or method listed is relevant to your study. If you are not sure if a list item applies to your research, read the appropriate section before selecting a response.

Materials & experimental systems

n/a	Involved in the study
☐	☒ Antibodies
☐	☒ Eukaryotic cell lines
☒	☐ Palaeontology and archaeology
☐	☒ Animals and other organisms
☒	☐ Clinical data
☒	☐ Dual use research of concern
☒	☐ Plants

Methods

n/a	Involved in the study
☐	☒ ChIP-seq
☐	☐ Flow cytometry
☐	☐ MRI-based neuroimaging

Antibodies

Antibodies used

BMAL1 (CellSignaling, D2L7G, #14020) - 1:2000
CLOCK (CellSignaling, D45B10 - #5157) - 1:2000
IgG (CellSignaling, #2729) - - 1:5000
PROX1 (ProteinTech, #11067-2-AP) - 1:2000
HNF1B (NovusBio, #NBP1-89680) - 1:2000
HOXA5 (Abcam, EPR2825(2), #ab140636) - 1:2000
ACTIN (SantaCruz, #sc-47778) 1:5000
VINCULIN (Sigma-Aldrich, #V9264) - 1:5000
MYC-Tag (CellSignaling, 9B11, #2276) - 1:5000
anti-rabbit IgG, HRP-linked antibody (CellSignaling, #7074) - 1:5000
anti-mouse IgG, HRP-linked antibody (CellSignaling, #7076) - 1:5000

Validation

Validation data are available for all antibodies from the manufacturer. Please refer to the references and validation information provided in the links supplied by the manufacturer:

BMAL1 (CellSignaling, D2L7G, #14020) - https://www.cellsignal.com/products/primary-antibodies/bmal1-d2l7g-rabbit-monoclonal-antibody/14020

CLOCK (CellSignaling, D45B10 - #5157) - https://www.cellsignal.com/products/primary-antibodies/clock-d45b10-rabbit-monoclonal-antibody/5157

IgG (CellSignaling, #2729) - https://www.cellsignal.com/products/primary-antibodies/normal-rabbit-igg/2729
PROX1 (ProteinTech, #11067-2-AP) - https://www.ptglab.com/products/PROX1-Antibody-11067-2-AP.htm?srsltid=AfmBOoo3UTut3Mt9V9tcDbjeJRIBScwWedn6w9SZx0cY8-CsAAl0YMuX

HNF1B (NovusBio, #NBP1-89680) - https://www.novusbio.com/products/tcf-2-hnf-1-beta-antibody_nbp1-89680?srsltid=AfmBOopGyNQFEi_bjJUj9o-gIR4sAQRRHMJLCSMbipb-ZXFrpJhMEZvS
HOXA5 (Abcam, EPR2825(2), #ab140636) - https://www.abcam.com/en-us/products/primary-antibodies/hoxa5-antibody-epr28252-ab140636

ACTIN (SantaCruz, #sc-47778) - https://www.scbt.com/de/p/beta-actin-antibody-c4?srsltid=AfmBOooZZoXZ4B_UO44p7uCyRHFX1V3bGXxkAkc4HGcFNnWLCzTVQH9w

VINCULIN (Sigma-Aldrich, #V9264) - https://www.sigmaaldrich.com/DE/en/product/sigma/v9264?srsltid=AfmBOorrjrJiQy1-jocj8a_TLUi4YUG0NMixRR1BVUXE_aJ3iaIT0rxX

MYC-Tag (CellSignaling, 9B11, #2276) - https://www.cellsignal.com/products/primary-antibodies/myc-tag-9b11-mouse-monoclonal-antibody/2276

anti-rabbit IgG, HRP-linked antibody (CellSignaling, #7074) - https://www.cellsignal.com/products/secondary-antibodies/anti-rabbit-igg-hrp-linked-antibody/7074?srsltid=AfmBOorBcOvTkLRJEXb2VMznxF611yAzQN03_snFqTymRPi1FXPSQE0B

anti-mouse IgG, HRP-linked antibody (CellSignaling, #7076) - https://www.cellsignal.com/products/secondary-antibodies/anti-mouse-igg-hrp-linked-antibody/7076?srsltid=AfmBOorI40F8ndwM3___33YPLTiVspK7-NkilI2KaS-7TMf_VOUsalmV

Eukaryotic cell lines

Policy information about cell lines and Sex and Gender in Research

Cell line source(s)	DihXY-WT, a mouse hepatocellular carcinoma cell line, was a gift from Steve A. Kay from Keck School of Medicine, University of Southern California, LA (paper:. HK-2, WT, Human proximal tubular epithelial cell line and HEK293T-WT, Human embryonic kidney cells were gift from Dr. Maciej Lech Medizinische Klinik und Poliklinik IV, LMU-Munich<br>DihXY-Bmal1-KO, DihXY-Prox1-gRNA1-KO, DihXY-Prox1-gRNA2-KO, DihXY-Hnf1b-KO, DihXY-Hoxa5-KO, HK-2-Bmal1-KO, HK-2-Prox1-KO, HK-2-Hnf1b-KO were generated in this study.
Authentication	All knockout cells were validated by immunoblotting.
Mycoplasma contamination	Cell lines were negative for mycoplasma
Commonly misidentified lines<br>(See ICLAC register)	No commonly misidentified cell line was used.

Animals and other research organisms

Policy information about studies involving animals; ARRIVE guidelines recommended for reporting animal research, and Sex and Gender in Research

Laboratory animals	8-12 weeks old C57BL/6N mice
Wild animals	This study did not involve wild animals.
Reporting on sex	Both male and female mice were used in a randomized manner.
Field-collected samples	This study did not involve field-collected samples
Ethics oversight	All animal maintenance and experimental procedures were carried out in accordance with the European law regarding protection of animal welfare and with approval by the local government authorities (animal facility registered for breeding and use of animals for scientific purpose KVR-I/221-TA166/22_03_06)

Note that full information on the approval of the study protocol must also be provided in the manuscript.

Plants

Seed stocks	*Report on the source of all seed stocks or other plant material used. If applicable, state the seed stock centre and catalogue number. If plant specimens were collected from the field, describe the collection location, date and sampling procedures.*
Novel plant genotypes	*Describe the methods by which all novel plant genotypes were produced. This includes those generated by transgenic approaches, gene editing, chemical/radiation-based mutagenesis and hybridization. For transgenic lines, describe the transformation method, the number of independent lines analyzed and the generation upon which experiments were performed. For gene-edited lines, describe the editor used, the endogenous sequence targeted for editing, the targeting guide RNA sequence (if applicable) and how the editor was applied.*
Authentication	*Describe any authentication procedures for each seed stock used or novel genotype generated. Describe any experiments used to assess the effect of a mutation and, where applicable, how potential secondary effects (e.g. second site T-DNA insertions, mosiacism, off-target gene editing) were examined.*

ChIP-seq

Data deposition

☒ Confirm that both raw and final processed data have been deposited in a public database such as GEO.

☒ Confirm that you have deposited or provided access to graph files (e.g. BED files) for the called peaks.

Data access links<br>*May remain private before publication.*	*For "Initial submission" or "Revised version" documents, provide reviewer access links. For your "Final submission" document, provide a link to the deposited data.*
Files in database submission	*Provide a list of all files available in the database submission.*
Genome browser session<br>(e.g. UCSC)	*Provide a link to an anonymized genome browser session for "Initial submission" and "Revised version" documents only, to enable peer review. Write "no longer applicable" for "Final submission" documents.*

Methodology

Replicates	*Describe the experimental replicates, specifying number, type and replicate agreement.*

Sequencing depth

Describe the sequencing depth for each experiment, providing the total number of reads, uniquely mapped reads, length of reads and whether they were paired- or single-end.

Antibodies

Describe the antibodies used for the ChIP-seq experiments; as applicable, provide supplier name, catalog number, clone name, and lot number.

Peak calling parameters

Specify the command line program and parameters used for read mapping and peak calling, including the ChIP, control and index files used.

Data quality

Describe the methods used to ensure data quality in full detail, including how many peaks are at FDR 5% and above 5-fold enrichment.

Software

Describe the software used to collect and analyze the ChIP-seq data. For custom code that has been deposited into a community repository, provide accession details.

Flow Cytometry

Plots

Confirm that:

☐ The axis labels state the marker and fluorochrome used (e.g. CD4-FITC).

☐ The axis scales are clearly visible. Include numbers along axes only for bottom left plot of group (a 'group' is an analysis of identical markers).

☐ All plots are contour plots with outliers or pseudocolor plots.

☐ A numerical value for number of cells or percentage (with statistics) is provided.

Methodology

Sample preparation

Describe the sample preparation, detailing the biological source of the cells and any tissue processing steps used.

Instrument

Identify the instrument used for data collection, specifying make and model number.

Software

Describe the software used to collect and analyze the flow cytometry data. For custom code that has been deposited into a community repository, provide accession details.

Cell population abundance

Describe the abundance of the relevant cell populations within post-sort fractions, providing details on the purity of the samples and how it was determined.

Gating strategy

Describe the gating strategy used for all relevant experiments, specifying the preliminary FSC/SSC gates of the starting cell population, indicating where boundaries between "positive" and "negative" staining cell populations are defined.

☐ Tick this box to confirm that a figure exemplifying the gating strategy is provided in the Supplementary Information.

Magnetic resonance imaging

Experimental design

Design type

Indicate task or resting state; event-related or block design.

Design specifications

Specify the number of blocks, trials or experimental units per session and/or subject, and specify the length of each trial or block (if trials are blocked) and interval between trials.

Behavioral performance measures

State number and/or type of variables recorded (e.g. correct button press, response time) and what statistics were used to establish that the subjects were performing the task as expected (e.g. mean, range, and/or standard deviation across subjects).

Acquisition

Imaging type(s)

Specify: functional, structural, diffusion, perfusion.

Field strength

Specify in Tesla

Sequence & imaging parameters

Specify the pulse sequence type (gradient echo, spin echo, etc.), imaging type (EPI, spiral, etc.), field of view, matrix size, slice thickness, orientation and TE/TR/flip angle.

Area of acquisition

State whether a whole brain scan was used OR define the area of acquisition, describing how the region was determined.

Diffusion MRI ☐ Used ☐ Not used

Preprocessing

Preprocessing software | *Provide detail on software version and revision number and on specific parameters (model/functions, brain extraction, segmentation, smoothing kernel size, etc.).*

Normalization | *If data were normalized/standardized, describe the approach(es): specify linear or non-linear and define image types used for transformation OR indicate that data were not normalized and explain rationale for lack of normalization.*

Normalization template | *Describe the template used for normalization/transformation, specifying subject space or group standardized space (e.g. original Talairach, MNI305, ICBM152) OR indicate that the data were not normalized.*

Noise and artifact removal | *Describe your procedure(s) for artifact and structured noise removal, specifying motion parameters, tissue signals and physiological signals (heart rate, respiration).*

Volume censoring | *Define your software and/or method and criteria for volume censoring, and state the extent of such censoring.*

Statistical modeling & inference

Model type and settings | *Specify type (mass univariate, multivariate, RSA, predictive, etc.) and describe essential details of the model at the first and second levels (e.g. fixed, random or mixed effects; drift or auto-correlation).*

Effect(s) tested | *Define precise effect in terms of the task or stimulus conditions instead of psychological concepts and indicate whether ANOVA or factorial designs were used.*

Specify type of analysis: ☐ Whole brain ☐ ROI-based ☐ Both

Statistic type for inference | *Specify voxel-wise or cluster-wise and report all relevant parameters for cluster-wise methods.*

(See Eklund et al. 2016)

Correction | *Describe the type of correction and how it is obtained for multiple comparisons (e.g. FWE, FDR, permutation or Monte Carlo).*

Models & analysis

n/a	Involved in the study
☐	☐ Functional and/or effective connectivity
☐	☐ Graph analysis
☐	☐ Multivariate modeling or predictive analysis

Functional and/or effective connectivity | *Report the measures of dependence used and the model details (e.g. Pearson correlation, partial correlation, mutual information).*

Graph analysis | *Report the dependent variable and connectivity measure, specifying weighted graph or binarized graph, subject- or group-level, and the global and/or node summaries used (e.g. clustering coefficient, efficiency, etc.).*

Multivariate modeling and predictive analysis | *Specify independent variables, features extraction and dimension reduction, model, training and evaluation metrics.*
