# Supplementary material for: CLOCK/BMAL1 interactome uncovers homeodomain factors as tissue regulators
Source: Nat Cell Biol. 2026 Aug 6;28(9):1890–900. doi: 10.1038/s41556-026-02041-4 (PMC13561871; doi:10.1038/s41556-026-02041-4)
Supplement: Supplementary file 2 [file 41556_2026_2041_MOESM2_ESM.pdf]

nature portfolio

Peer Review File

CLOCK/BMAL1 interactome uncovers homeodomain factors as tissue regulators

Corresponding Author: Professor Maria Robles

Version 0:

Decision Letter:

Revise extended OD

*Please delete the link to your author homepage if you wish to forward this email to co-authors.

Dear Professor Robles,

Your manuscript, "CLOCK/BMAL1 interactome uncovers homeodomain factors as tissue regulators", has now been seen by 3 referees, who are experts in chronobiology (referee 1); molecular regulation of biological clocks (referee 2); and epigenomic transcription regulation (referee 3). As you will see from their comments (attached below) they find this work of potential interest, but have raised substantial concerns, which in our view would need to be addressed with considerable revisions before we can consider publication in Nature Cell Biology.

Nature Cell Biology editors discuss the referee reports in detail within the editorial team, including the chief editor, to identify key referee points that should be addressed with priority, and requests that are overruled as being beyond the scope of the current study. To guide the scope of the revisions, I have listed these points below. We are committed to providing a fair and constructive peer-review process, so please feel free to contact me if you would like to discuss any of the referee comments further.

In particular, it would be essential to:

A-Further experimentally test the timing of the interaction/transcription repression by homeodomain factors (Ref#1 pt 1, ref#2 minor pt 1)
B-Address with experiments all the reviewers concerns regarding the timing of the model (ref#1 pt 2, ref#3 pt 1, ref#3 pt 3)
C-Further experimentally test the proposed interaction between BMAL1 and PROX1 (referee#2 minor pt1, referee#3 pt5-6) and the referee points about strengthening the overall model (Ref#3 pts 4,8)

D- All other referee concerns pertaining to strengthening existing data, providing controls, methodological details, clarifications and textual changes, should also be addressed.

E-Finally please pay close attention to our guidelines on statistical and methodological reporting (listed below) as failure to do so may delay the reconsideration of the revised manuscript. In particular please provide:

- a Supplementary Figure including unprocessed images of all gels/blots in the form of a multi-page pdf file. Please ensure that blots/gels are labeled and the sections presented in the figures are clearly indicated.

- a Supplementary Table including all numerical source data in Excel format, with data for different figures provided as different sheets within a single Excel file. The file should include source data giving rise to graphical representations and statistical descriptions in the paper and for all instances where the figures present representative experiments of multiple independent repeats, the source data of all repeats should be provided.

We would be happy to consider a revised manuscript that would satisfactorily address these points, unless a similar paper is published elsewhere, or is accepted for publication in Nature Cell Biology in the meantime.

When revising the manuscript please:

- ensure that it conforms to our format instructions and publication policies (see below and www.nature.com/nature/authors/).

- provide a point-by-point rebuttal to the full referee reports verbatim, as provided at the end of this letter.

- provide the completed Editorial Policy Checklist (found here https://www.nature.com/authors/policies/Policy.pdf), and Reporting Summary (found here https://www.nature.com/authors/policies/ReportingSummary.pdf).

This is essential for reconsideration of the manuscript and these documents will be available to editors and referees in the event of peer review. For more information see http://www.nature.com/authors/policies/availability.html or contact me.

Nature Cell Biology is committed to improving transparency in authorship. As part of our efforts in this direction, we are now requesting that all authors identified as ‘corresponding author’ on published papers create and link their Open Researcher and Contributor Identifier (ORCID) with their account on the Manuscript Tracking System (MTS), prior to acceptance. ORCID helps the scientific community achieve unambiguous attribution of all scholarly contributions. You can create and link your ORCID from the home page of the MTS by clicking on ‘Modify my Springer Nature account’. For more information please visit please visit www.springernature.com/orcid.

Please submit the revised manuscript files and the point-by-point rebuttal to the referee comments using this link:

Link Redacted

*This url links to your confidential home page and associated information about manuscripts you may have submitted or be reviewing for us. If you wish to forward this email to co-authors, please delete the link to your homepage.

We would like to receive a revised submission within six months. We would be happy to consider a revision even after this timeframe, however if the resubmission deadline is missed and the paper is eventually published, the submission date will be the date when the revised manuscript was received.

We hope that you will find our referees' comments, and editorial guidance helpful. Please do not hesitate to contact me if there is anything you would like to discuss.

Best wishes,

Sabrya Carim

Sabrya Carim, PhD
(she/her/hers)
Senior Editor, Nature Cell Biology
Nature Portfolio

Springer Nature
The Campus, 4 Crinan Street, London N1 9XW, UK
sabrya.carim@springernature.com
https://orcid.org/0000-0001-9485-1938

Reviewers' Comments:

Reviewer #1 (Remarks to the Author):

This article constitutes an exciting story, adding new insight about the still not well described regulation of the tissue specific rhythmic transcription by the circadian clock. Using ChIP-MS, authors decipher the tissue specific dynamic of the BMAL1-CLOCK complex around the time of its maximal activity (ZT 4-10). Based on this data, they identified Homeodomain factors interacting with BMAL1 and modulating its activity in a tissue specific manner. This is globally a very clear and interesting article based on high standard quality data. Nevertheless, a few questions need to be answered before publication.
- The main issue is the timing of this interaction/transcription repression by the Homeodomain factors. Authors described an interaction at the time of maximal BMAL1 activity while the interaction with the Homeodomain factors has a repressive effect. This sounds somehow puzzling. What is the dynamic of this interaction? Is there a competition with the binding of the CRY/PER complex, supposed to not interact with BMAL1/CLOCK at that time? What is happening at ZT19, when BMAL1 reaches its minimum of activity? Answering these questions is required to understand the mechanism involved.
- What were the criteria for choosing the 3 Homeodoamain factors PROX1, HNF1B, and HOXA5? There is many transcription factors in the BMAL1 interactomes. What was the reason for choosing these ones?
- Another issue relies on the statistical analysis. Analyzing independently the 2 datasets (WT and KO) with JTK cycle is not a proper analysis of differential rhythmicity (PMID: 29098954). Authors must use one of the specific methods recently developed for this specific purpose. On the same idea, figures 5C, 5D, and ED7 must be supported by statistical analysis.
- Related to these results, what is the overlap between the BMAL1-PROX1 cistrome and the differentially rhythmic genes? Considering the broad distribution of phases, some target genes appear likely indirect, an observation that appears conflicting with the global increase in amplitude of clock genes. What is the impact of Prox1 deletion on non-rhythmic BMAL1 targets?
- Line 217, the sentence “this repressive function does not require DNA-binding” needs to be reformulated. Because there is no activation without DNA binding, there is also likely no repression.

Reviewer #1 (Remarks on Protocol(s)):

No specific comment on the method except for the statistical analysis section.

Reviewer #2 (Remarks to the Author):

Peer Review for CLOCK/BMAL1 interactome uncovers homeodomain factors as tissue regulators

Aygenli et al. use high-throughput mass spectrometry (MS-) and ChIP-based approaches to detect novel protein interactors of the core circadian BMAL1:CLOCK complex. This was extended to various tissues to detect tissue-specific interactions with homeodomain transcription factors namely, PROX1, HNF1B, and HOXA5 in the mouse liver, kidney, and lung, respectively. The high-throughput analysis alone can already serve as a rich resource for further studies on novel BMAL1:CLOCK interactors. Validation of the detected tissue-specific interactors in PROX1, HNF1B, and HOXA5, is comprehensive: protein-protein interaction is confirmed via IP, genome co-occupancy via ChIP, transcriptional repression via transactivation assays. Interestingly, AlphaFold predicts a common interaction interface occurring between the respective homeodomains and the BMAL1 TAD with the key residues validated by IP and transactivation assays. Finally, genetic knockout affecting amplitude and resulting in tissue-specific loss of rhythmicity rounds out the analysis of these novel BMAL1:CLOCK interactors and repressors, confirming their importance in supporting tissue-specific rhythmicity. While homeodomain TFs typically play roles in development, the authors propose that BMAL1:CLOCK repression by these homeodomain TFs may serve as a mechanism to buffer BMAL1:CLOCK activity, and thereby complex abundance during the activation phase. Overall, this work represents a significant conceptual advance and is of interest for a broad readership beyond the circadian and developmetal biology fields.

We do not have any major points requiring revision; the minor points listed below are optional. Additional requests could make the manuscript slightly different, but not necessarily better. We therefore feel that major revision requests would place the editors in a difficult position, as they would either need to overrule the reviewer or convey revision requests to the authors that would unnecessarily consume the time and resources of the laboratories involved.

Minor points
1. The authors may consider performing a CoIP with both BMAL1 and CLOCK and testing for the interaction with PROX1, HNF1B, and HOXA5. Given that the authors also propose that the PAS core domain is important for the interaction with the homeodomain TF, a complete BMAL1:CLOCK complex may result in a more definitive signal showing protein-protein interaction.
2. The authors may consider including Extended Data Figs. 6 A and B to Fig. 4 as Fig 4 I and J as this is an interesting point to consider: while mutation of E614 and L619 to alanine does not affect protein-protein interaction, they do affect the repressive capacities of PROX1, HNF1B, and HOXA5 (and CRY1 as well, the authors might have missed adding those asterisks in the figure).
3. The authors may provide a comment explaining the 10-fold decrease in transactivation between Fig 4F and 4H.
4. In Figures 5 A and B, the authors may normalize the relative mRNA levels to the control to more clearly show the fold-level increase in expression upon PROX1 or HNF1B KO.
5. In line 356, the formatting for citation 42 seems to be incorrect.
6. In general, the citation formatting throughout the text is slightly incorrect as it includes a space between the text and the citation. As an example, in line 5, “outputs 1-3” should be reformatted to “outputs1-3”. All citations should be reformatted appropriately.

Fidel Serrano and Michael Brunner

Reviewer #3 (Remarks to the Author):

The authors map the BMAL1 and CLOCK interactomes across three mouse tissues (liver, kidney, lung) at three time points (ZT4, ZT7, ZT10) and identify 1,463 high-confidence interactors, including tissue-specific interacting ttranscription factors (TFs). Among these were three homeodomain TFs (PROX1, HNF1B, HOXA5), which were shown to directly bind and co-occupy most BMAL1 genomic sites. In addition, AlphaFold-based structural predictions followed by mutational analyses suggested that homeodomain (HD) factors repress BMAL1/CLOCK activity.While the study is conceptually interesting, there are major concerns regarding experimental design, data interpretation, and terminology that are problematic for this study. For example, In terms of the timing, the authors only study three closely spaced time points. Moreover, while BMAL1 DNA-binding activity peaks in liver around ZT7, this is majorly delayed from the protein peak around ZT16-20, and it is not clear whether this unusual delay is also seen in lung and kidney. Moreover, the authors focus on the 3 homeodomain TFs without clear rationale, and suggest that these factors couple the clock to organ identity without experimental evidence.

Major Comments

1, The authors identified 1,436 interactors, and state on line 147 that 5-10% of these are transcriptional regulators. Of these they focused on three homeodomain TFs, which they justify based on the annotation term "developmental processes" in Fig. 2F. However, it is not clear how many other TFs are present in the interactors belonging to this patway, and why these specific TFs were selected.

2. The authors selected ZT7 as the peak of BMAL1/CLOCK chromatin binding, based on the data in reference 12. Other groups have found similar results, however this peak of chromatin binding occurs many hours after the peak of BMAL mRNA and protein expression. This phenomenon is poorly understood, and to this reviewer’s knowledge has not been shown in kidney or lung. These details are highly relevant to the present study, especially sincd tissue-specific phase differences have been reported for peripheral clocks. . The authors should provide supporting data demonstrating that BMAL1/CLOCK DNA binding indeed peaks at ZT7 in kidney and lung.

3. Related to this, in Line 149, the statement that “spatiotemporal analysis demonstrates that the chromatin-associated protein interaction landscape of CLOCK/BMAL1 is shaped predominantly by tissue context, rather than circadian time” is an overstatement. With only three time points sampled within a relatively narrow window, it is difficult to conclude that circadian time plays a minor role. Additional time points or a more cautious interpretation would be warranted.

4. Moreover, multiple BMAL1 mouse liver ChIP-seq datasets across tissues are already available. The authors should compare their own ChIP-seq datasets with these, for example in liver (PMID: 30804225, 22936566), to strengthen confidence in the conclusions.

5. Figure 1C and Table S1 indicate that BMAL1 and CLOCK share 379 interactors in liver, including PROX1. However, in Figure 3B, PROX1 ChIP fails to detect CLOCK, leading the authors to propose that PROX1 binds the BMAL1 transactivation domain (TAD) without interacting with CLOCK. However, since CLOCK is bound to BMAL1, and PROX1 is present in CLOCK ChIP-MS (Table S1), it is why CLOCK is not present in the PROX1 ChIP-MS via the presence of BMAL1.

6. The authors do not measure circadian oscillation of BMAL1 In mouse liver, but prior studies have shown that despite the large amplitude of Bmal1 mRNA, BMAL1 protein circadian oscillation exhibits a moderate amplitude in liver. However, the temporal expression profile of PROX1 is not addressed in liver (or the other tissues). It would be important to determine whether PROX1 protein levels or its interaction with BMAL1 are also rhythmic. This information is critical for understanding whether PROX1-mediated repression is circadian phase-dependent.

7. The authors use AlphaFold to predict the interaction interface and identify the BMAL1 TAD as critical for binding homeodomain factors. While informative, the subsequent functional analyses rely on overexpression-based mutagenesis. Given the central role of this interaction in the proposed model, endogenous mutation (e.g., CRISPR-mediated editing) would provide stronger and more physiologically relevant evidence for the role of the BMAL1 TAD in homeodomain factor–mediated repression.

8. In Figure 5E, Prox1 knockout in diHXY cells retains rhythmicity in 98 genes, including core clock genes, but disrupts rhythmicity in 1,171 genes. It would be informative to assess how BMAL1 expression and/or activity is affected following Prox1 deletion. Additionally, the authors should clarify how loss of rhythmicity in a large gene set occurs despite preserved core clock gene oscillations, and whether this reflects impaired clock output rather than clock function.

Minor Comments

1. In Line 169, the term “reverse ChIP/MS” is misleading, as it could be interpreted as locus-centric chromatin pulldown followed by proteomics. In this context, “reciprocal ChIP-MS” would be a more accurate and standard term.

2. Protein data should be made available to reviewers via a public database such as ProteomeXchange.

GUIDELINES FOR MANUSCRIPT SUBMISSION TO NATURE CELL BIOLOGY

READABILITY OF MANUSCRIPTS – Nature Cell Biology is read by cell biologists from diverse backgrounds, many of whom are not native English speakers. Authors should aim to communicate their findings clearly, explaining technical jargon that might be unfamiliar to non-specialists, and avoiding non-standard abbreviations. Titles and abstracts should concisely communicate the main findings of the study, and the background, rationale, results and conclusions should be clearly explained in the manuscript in a manner accessible to a broad cell biology audience. Nature Cell Biology uses British spelling.

MANUSCRIPT FORMAT – please follow the guidelines listed in our Guide to Authors regarding manuscript formats at Nature Cell Biology.

TITLE – should be no more than 100 characters including spaces, without punctuation and avoiding technical terms, abbreviations, and active verbs..

AUTHOR NAMES – should be given in full.

AUTHOR AFFILIATIONS – should be denoted with numerical superscripts (not symbols) preceding the names. Full addresses should be included, with US states in full and providing zip/post codes. The corresponding author is denoted by: "Correspondence should be addressed to [initials]."

ABSTRACT AND MAIN TEXT – please follow the guidelines that are specific to the format of your manuscript, as listed in our Guide to Authors (http://www.nature.com/ncb/pdf/ncb_gta.pdf) Briefly, Nature Cell Biology Articles, Resources and Technical Reports have 3500 words, including a 150 word abstract, and the main text is subdivided in Introduction, Results, and Discussion sections. Nature Cell Biology Letters have up to 2500 words, including a 180 word introductory paragraph (abstract), and the text is not subdivided in sections.

ACKNOWLEDGEMENTS – should be kept brief. Professional titles and affiliations are unnecessary. Grant numbers can be listed.

AUTHOR CONTRIBUTIONS – must be included after the Acknowledgements, detailing the contributions of each author to the paper (e.g. experimental work, project planning, data analysis etc.). Each author should be listed by his/her initials.

FINANCIAL AND NON-FINANCIAL COMPETING INTERESTS – the authors must include one of three declarations: (1) that they have no financial and non-financial competing interests; (2) that they have financial and non-financial competing interests; or (3) that they decline to respond, after the Author Contributions section. This statement will be published with the article, and in cases where financial and non-financial competing interests are declared, these will be itemized in a web supplement to the article. For further details please see https://www.nature.com/licenceforms/nrg/competing-interests.pdf.

REFERENCES – are limited to a total of 70 for Articles, Resources, Technical Reports; and 40 for Letters. This includes references in the main text and Methods combined. References must be numbered sequentially as they appear in the main text, tables and figure legends and Methods and must follow the precise style of Nature Cell Biology references. References only cited in the Methods should be numbered consecutively following the last reference cited in the main text. References only associated with Supplementary Information (e.g. in supplementary legends) do not count toward the total reference limit and do not need to be cited in numerical continuity with

references in the main text. Only published papers can be cited, and each publication cited should be included in the numbered reference list, which should include the manuscript titles. Footnotes are not permitted.

METHODS – Nature Cell Biology publishes methods online. The methods section should be provided as a separate Word document, which will be copyedited and appended to the manuscript PDF, and incorporated within the HTML format of the paper.

Methods should be written concisely, but should contain all elements necessary to allow interpretation and replication of the results. As a guideline, Methods sections typically do not exceed 3,000 words. The Methods should be divided into subsections listing reagents and techniques. When citing previous methods, accurate references should be provided and any alterations should be noted. Information must be provided about: antibody dilutions, company names, catalogue numbers and clone numbers for monoclonal antibodies; sequences of RNAi and cDNA probes/primers or company names and catalogue numbers if reagents are commercial; cell line names, sources and information on cell line identity and authentication. Animal studies and experiments involving human subjects must be reported in detail, identifying the committees approving the protocols. For studies involving human subjects/samples, a statement must be included confirming that informed consent was obtained. Statistical analyses and information on the reproducibility of experimental results should be provided in a section titled "Statistics and Reproducibility".

All Nature Cell Biology manuscripts submitted on or after March 21 2016 must include a Data availability statement at the end of the Methods section. For Springer Nature policies on data availability see http://www.nature.com/authors/policies/availability.html; for more information on this particular policy see http://www.nature.com/authors/policies/data/data-availability-statements-data-citations.pdf. The Data availability statement should include:

• Accession codes for primary datasets (generated during the study under consideration and designated as "primary accessions") and secondary datasets (published datasets reanalysed during the study under consideration, designated as "referenced accessions"). For primary accessions data should be made public to coincide with publication of the manuscript. A list of data types for which submission to community-endorsed public repositories is mandated (including sequence, structure, microarray, deep sequencing data) can be found here http://www.nature.com/authors/policies/availability.html#data.

• Unique identifiers (accession codes, DOIs or other unique persistent identifier) and hyperlinks for datasets deposited in an approved repository, but for which data deposition is not mandated (see here for details http://www.nature.com/sdata/data-policies/repositories).

• At a minimum, please include a statement confirming that all relevant data are available from the authors, and/or are included with the manuscript (e.g. as source data or supplementary information), listing which data are included (e.g. by figure panels and data types) and mentioning any restrictions on availability.

• If a dataset has a Digital Object Identifier (DOI) as its unique identifier, we strongly encourage including this in the Reference list and citing the dataset in the Methods.

We recommend that you upload the step-by-step protocols used in this manuscript to protocols.io. More details can found at https://www.protocols.io/help/publish-articles.

DISPLAY ITEMS – main display items are limited to 6-8 main figures and/or main tables for Articles, Resources, Technical Reports; and 5 main figures and/or main tables for Letters. For Supplementary Information see below.

FIGURES – Colour figure publication costs $600 for the first, and $300 for each subsequent colour figure. All panels of a multi-panel figure must be logically connected and arranged as they would appear in the final version. Unnecessary figures and figure panels should be avoided (e.g. data presented in small tables could be stated briefly in the text instead).

All imaging data should be accompanied by scale bars, which should be defined in the legend.
Cropped images of gels/blots are acceptable, but need to be accompanied by size markers, and to retain visible background signal within the linear range (i.e. should not be saturated). The boundaries of panels with low background have to be demarked with black lines. Splicing of panels should only be considered if unavoidable, and must be clearly marked on the figure, and noted in the legend with a statement on whether the samples were obtained and processed simultaneously. Quantitative comparisons between samples on different gels/blots are discouraged; if this is unavoidable, it should only be performed for samples derived from the same experiment with gels/blots were processed in parallel, which needs to be stated in the legend.

Figures should be provided at approximately the size that they are to be printed at (single column is 86 mm, double column is 170 mm) and should not exceed an A4 page (8.5 x 11"). Reduction to the scale that will be used on the page is not necessary, but multi-panel figures should be sized so that the whole figure can be reduced by the same amount at the smallest size at which essential details in each panel are visible. In the interest of our colour-blind readers we ask that you avoid using red and green for contrast in figures. Replacing red with magenta and green with turquoise are two possible colour-safe alternatives. Lines with widths of less than 1 point should be avoided. Sans serif typefaces, such as Helvetica (preferred) or Arial should be used. All text that forms part of a figure should be rewritable and removable.

We accept files from the following graphics packages in either PC or Macintosh format:

- For line art, graphs, charts and schematics we prefer Adobe Illustrator (.AI), Encapsulated PostScript (.EPS) or Portable Document Format (.PDF). Files should be saved or exported as such directly from the application in which they were made, to allow us to restyle them according to our journal house style.

- We accept PowerPoint (.PPT) files if they are fully editable. However, please refrain from adding PowerPoint graphical effects to objects, as this results in them outputting poor quality raster art. Text used for PowerPoint figures should be Helvetica (preferred) or Arial.

- We do not recommend using Adobe Photoshop for designing figures, but we can accept Photoshop generated (.PSD or .TIFF) files only if each element included in the figure (text, labels, pictures, graphs, arrows and scale bars) are on separate layers. All text should be

editable in ‘type layers’ and line-art such as graphs and other simple schematics should be preserved and embedded within 'vector smart objects' - not flattened raster/bitmap graphics.

- Some programs can generate Postscript by 'printing to file' (found in the Print dialogue). If using an application not listed above, save the file in PostScript format or email our Art Editor, Allen Beattie for advice (a.beattie@nature.com).

Regardless of format, all figures must be vector graphic compatible files, not supplied in a flattened raster/bitmap graphics format, but should be fully editable, allowing us to highlight/copy/paste all text and move individual parts of the figures (i.e. arrows, lines, x and y axes, graphs, tick marks, scale bars etc.). The only parts of the figure that should be in pixel raster/bitmap format are photographic images or 3D rendered graphics/complex technical illustrations.

All placed images (i.e. a photo incorporated into a figure) should be on a separate layer and independent from any superimposed scale bars or text. Individual photographic images must be a minimum of 300+ DPI (at actual size) or kept constant from the original picture acquisition and not decreased in resolution post image acquisition. All colour artwork should be RGB format.

FIGURE LEGENDS – must not exceed 350 words for each figure to allow fit on a single printed NCB page together with the figure. They must include a brief title for the whole figure, and short descriptions of each panel with definitions of the symbols used, but without detailing methodology.

TABLES – main tables should be provided as individual Word files, together with a brief title and legend. For supplementary tables see below.

SUPPLEMENTARY INFORMATION – Supplementary information is material directly relevant to the conclusion of a paper, but which cannot be included in the printed version in order to keep the manuscript concise and accessible to the general reader. Supplementary information is an integral part of a Nature Cell Biology publication and should be prepared and presented with as much care as the main display item, but it must not include non-essential data or text, which may be removed at the editor's discretion. All supplementary material is fully peer-reviewed and published online as part of the HTML version of the manuscript. Supplementary Figures and Supplementary Notes are appended at the end of the main PDF of the published manuscript.

Supplementary items should relate to a main text figure, wherever possible, and should be mentioned sequentially in the main manuscript, designated as Supplementary Figure, Table, Video, or Note, and numbered continuously (e.g. Supplementary Figure 1, Supplementary Figure 2, Supplementary Table 1, Supplementary Table 2 etc.).

Unprocessed scans of all key data generated through electrophoretic separation techniques need to be presented in a supplementary figure that should be labelled and numbered as the final supplementary figure, and should be mentioned in every relevant figure legend. This figure does not count towards the total number of figures and is the only figure that can be displayed over multiple pages, but should be provided as a single file, in PDF or TIFF format. Data in this figure can be displayed in a relatively informal style, but size markers and the figures panels corresponding to the presented data must be indicated.

The total number of Supplementary Figures (not including the “unprocessed scans” Supplementary Figure) should not exceed the number of main display items (figures and/or tables (see our Guide to Authors and March 2012 editorial http://www.nature.com/ncb/authors/submit/index.html#suppinfo; http://www.nature.com/ncb/journal/v14/n3/index.html#ed). No restrictions apply to Supplementary Tables or Videos, but we advise authors to be selective in including supplemental data.

Each Supplementary Figure should be provided as a single page and as an individual file in one of our accepted figure formats and should be presented according to our figure guidelines (see above). Supplementary Tables should be provided as individual Excel files. Supplementary Videos should be provided as .avi or .mov files up to 50 MB in size. Supplementary Figures, Tables and Videos much be accompanied by a separate Word document including titles and legends.

GUIDELINES FOR EXPERIMENTAL AND STATISTICAL REPORTING

REPORTING REQUIREMENTS – To improve the quality of methods and statistics reporting in our papers we have recently revised the reporting checklist we introduced in 2013. We are now asking all life sciences authors to complete two items: an Editorial Policy Checklist (found here https://www.nature.com/authors/policies/Policy.pdf) that verifies compliance with all required editorial policies and a reporting summary (found here https://www.nature.com/authors/policies/ReportingSummary.pdf) that collects information on experimental design and reagents. These documents are available to referees to aid the evaluation of the manuscript. Please note that these forms are dynamic ‘smart pdfs’ and must therefore be downloaded and completed in Adobe Reader. We will then flatten them for ease of use by the reviewers. If you would like to reference the guidance text as you complete the template, please access these flattened versions at http://www.nature.com/authors/policies/availability.html.

STATISTICS – Wherever statistics have been derived the legend needs to provide the n number (i.e. the sample size used to derive statistics) as a precise value (not a range), and define what this value represents. Error bars need to be defined in the legends (e.g. SD, SEM) together with a measure of centre (e.g. mean, median). Box plots need to be defined in terms of minima, maxima, centre, and percentiles. Ranges are more appropriate than standard errors for small data sets. Wherever statistical significance has been derived, precise p values need to be provided and the statistical test used needs to be stated in the legend. Statistics such as error bars must not be derived from $n<3$. For sample sizes of $n<5$ please plot the individual data points rather than providing bar graphs. Deriving statistics from technical replicate samples, rather than biological replicates is strongly discouraged. Wherever statistical significance has been derived, precise p values need to be provided and the statistical test stated in the legend.

Information on how many times each experiment was repeated independently with similar results needs to be provided in the legends and/or Methods for all experiments, and in particular wherever representative experiments are shown.

We strongly recommend the presentation of source data for graphical and statistical analyses as a separate Supplementary Table, and request that source data for all independent repeats are provided when representative experiments of multiple independent repeats, or averages of two independent experiments are presented. This supplementary table should be in Excel format, with data for different figures provided as different sheets within a single Excel file. It should be labelled and numbered as one of the supplementary tables, titled “Statistics Source Data”, and mentioned in all relevant figure legends.

--------- Please don't hesitate to contact NCB@nature.com should you have queries about any of the above requirements ---------

Version 1:

Decision Letter:

Our ref: NCB-A60432A

9th June 2026

Dear Dr. Robles,

Thank you for submitting your revised manuscript "CLOCK/BMAL1 interactome uncovers homeodomain factors as tissue regulators" (NCB-A60432A). It has now been seen by a subset of the original referees and their comments are below. Please note that as Reviewer 3 was unable to review the revised version, Reviewer 4 with overlapping expertise commented on the responses to Reviewer 3's concerns on the previous version of the manuscript. The reviewers find that the paper has improved in revision, and therefore we'll be happy in principle to publish it in Nature Cell Biology, pending minor revisions to satisfy the referees' final requests and to comply with our editorial and formatting guidelines.

Please ensure to address the remaining points of the reviewers in the revised manuscript.

Please note that our articles must have 5 to 8 main figures and they can have up to 10 ED figures. Please ensure that all figures fit into a single page (not multiple pages) and adhere to a maximum page size of roughly 180mm wide x 200mm high and use a font size of no smaller than 6pt Arial or Helvetica throughout the figures, to ensure legibility of the figures once resized for publication. Also, please use the full page space to fill the figure and remove the figure labels from the Figures.

If the current version of your manuscript is in a PDF format, please email us a copy of the file in an editable format (Microsoft Word or LaTex)-- we can not proceed with PDFs at this stage.

We are now performing detailed checks on your paper and will send you a checklist detailing our editorial and formatting requirements in about 7-10 days. **Please do not upload the final materials and make any revisions until you receive this additional information from us.**

Thank you again for your interest in Nature Cell Biology Please do not hesitate to contact me if you have any questions.

Best wishes,
Sabrya.

Sabrya Carim, PhD
(she/her/hers)
Senior Editor, Nature Cell Biology
Nature Portfolio

Springer Nature
The Campus, 4 Crinan Street, London N1 9XW, UK
sabrya.carim@springernature.com
https://orcid.org/0000-0001-9485-1938

Reviewer #1 (Remarks to the Author):

Authors have adequately and convincingly addressed all reviewers’ queries. Nevertheless, the question regarding the overlap of BMAL1-PROX1 cistromes is of importance and this should be included in the manuscript, at least as a supplementary figure.

Reviewer #1 (Remarks on Protocol(s)):

No additional comment.

Reviewer #2 (Remarks to the Author):

The authors have adequately addressed all points raised by us.

Reviewer #4 (Remarks to the Author):

I'm not a formal reviewer of the manuscript, but read the other reviews and find the revised manuscript is responsive. Specifically,

1. Selection of PROX1, HNF1B, and HOXA5

Addressed. The authors provide a reasonable rationale for focusing on these three tissue-specific homeodomain factors for mechanistic follow-up. I don't think characterization of additional candidates will add to this study.

2. BMAL1/CLOCK occupancy in kidney and lung

Addressed. The authors provide literature support for selecting ZT7 as a period of high BMAL1/CLOCK chromatin occupancy in all three tissues. Could it be done differently? Sure. What would it add though?

3. Tissue versus time

Addressed. The authors revised the statement to better reflect the scope of the data.

4. Comparison with published BMAL1 ChIP-seq datasets

Addressed. The authors clarify that this analysis was already in the original manuscript. This was a reviewer miss.

5. PROX1–BMAL1 versus PROX1–CLOCK interactions

Addressed. The authors provide additional experimental evidence supporting a model in which PROX1 interacts directly with BMAL1 and associates with CLOCK indirectly through the CLOCK/BMAL1 complex.

6. Rhythmicity of PROX1 and phase-dependent repression

Addressed. The authors provide evidence that BMAL1 nuclear abundance is rhythmic whereas PROX1 abundance is relatively stable, supporting a model in which the interaction is regulated in a time-dependent manner rather than simply by protein abundance.

7. BMAL1 TAD mutagenesis

Adequately addressed. While endogenous genome editing would provide additional support, I agree with the authors that such experiments are beyond the scope of the current study. The combination of structural prediction, mutagenesis, interaction, and functional assays supports the conclusions presented.

8. Effects of Prox1 deletion on rhythmic transcription

Addressed. The authors provide additional data showing that BMAL1 protein abundance is unchanged following Prox1 deletion and clarify that the observed phenotype reflects disruption of circadian output rather than the core clock itself. Output is the vast majority of regulation of physiology and what most people care about.

Overall, I believe the authors have addressed the concerns raised by Reviewer #3. The revised manuscript is strengthened by the additional analyses and experiments, and don't see any remaining issues that would preclude publication.'

Reviewer #4 (Remarks on Protocol(s)):

What protocol?

Version 2:

Decision Letter:

Dear Dr Robles,

I am pleased to inform you that your manuscript, "CLOCK/BMAL1 interactome uncovers homeodomain factors as tissue regulators", has now been accepted for publication in Nature Cell Biology. Congratulations!

Thank you for sending us the final manuscript files to be processed for print and online production, and for returning the manuscript checklists and other forms. Your manuscript will now be passed to our production team who will be in contact with you if there are any questions with the production quality of supplied figures and text.

Over the next few weeks, your paper will be copyedited to ensure that it conforms to Nature Cell Biology style. Once your paper is typeset, you will receive an email with a link to choose the appropriate publishing options for your paper and our Author Services team will be in touch regarding any additional information that may be required.

After the grant of rights is completed, you will receive a link to your electronic proof via email with a request to make any corrections within

48 hours. If, when you receive your proof, you cannot meet this deadline, please inform us at rjsproduction@springernature.com immediately.

You will not receive your proofs until the publishing agreement has been received through our system.

Due to the importance of these deadlines, we ask that you please let us know now whether you will be difficult to contact over the next month. If this is the case, we ask you provide us with the contact information (email, phone and fax) of someone who will be able to check the proofs on your behalf, and who will be available to address any last-minute problems.

If you have any questions about our publishing options, costs, Open Access requirements, or our legal forms, please contact ASJournals@springernature.com

Once your paper has been scheduled for online publication, the Nature press office will be in touch to confirm the details. An online order form for reprints of your paper is available at https://www.nature.com/reprints/author-reprints.html. All co-authors, authors' institutions and authors' funding agencies can order reprints using the form appropriate to their geographical region.

Publication is conditional on the manuscript not being published elsewhere and on there being no announcement of this work to any media outlet until the online publication date in Nature Cell Biology.

You may wish to make your media relations office aware of your accepted publication, in case they consider it appropriate to organize some internal or external publicity. Once your paper has been scheduled you will receive an email confirming the publication details. This is normally 3-4 working days in advance of publication. If you need additional notice of the date and time of publication, please let the production team know when you receive the proof of your article to ensure there is sufficient time to coordinate. Further information on our embargo policies can be found here: https://www.nature.com/authors/policies/embargo.html

Please note that *Nature Cell Biology* is a Transformative Journal (TJ). Authors may publish their research with us through the traditional subscription access route or make their paper immediately open access through payment of an article-processing charge (APC). Authors will not be required to make a final decision about access to their article until it has been accepted. Find out more about Transformative Journals

Authors may need to take specific actions to achieve compliance with funder and institutional open access mandates. If your research is supported by a funder that requires immediate open access (e.g. according to Plan S principles or the NIH public access policy) then you should select the gold OA route, and we will direct you to the compliant route where possible. Because authors warrant under our subscription licensing terms that they haven’t committed to licensing any version of their article under a licence inconsistent with the terms of our agreement – including the applicable embargo period – publication under the subscription model isn’t suitable for authors whose funders require no embargo.

To assist our authors in disseminating their research to the broader community, our SharedIt initiative provides you with a unique shareable link that will allow anyone (with or without a subscription) to read the published article. Recipients of the link with a subscription will also be able to download and print the PDF.

If your paper includes color figures, please be aware that in order to help cover some of the additional cost of four-color reproduction, Nature Portfolio charges our authors a fee for the printing of their color figures. Please contact our offices for exact pricing and details.

As soon as your article is published, you will receive an automated email with your shareable link.

If you have not already done so, we strongly recommend that you upload the step-by-step protocols used in this manuscript to protocols.io (https://protocols.io), an open online resource that allows researchers to share their detailed experimental know-how. All uploaded protocols are made freely available and are assigned DOIs for ease of citation. Protocols and Nature Portfolio journal papers in which they are used can be linked to one another, and this link is clearly and prominently visible in the online versions of both. Authors who performed the specific experiments can act as primary authors for the Protocol as they will be best placed to share the methodology details, but the Corresponding Author of the present research paper should be included as one of the authors. By uploading your Protocols onto protocols.io, you are enabling researchers to more readily reproduce or adapt the methodology you use, as well as increasing the visibility of your protocols and papers. You can also establish a dedicated workspace to collect your lab Protocols. Further information can be found at https://www.protocols.io/help/publish-articles.

You can use a single sign-on for all your accounts, view the status of all your manuscript submissions and reviews, access usage statistics for your published articles and download a record of your refereeing activity for the Nature Portfolio.

Nature Cell Biology encourages authors presenting evidence for cell, biological, molecular, and genetic interactions to consider communicating these findings using Biofactoid (https://biofactoid.org/). This tool helps users share a searchable representation of interactions (e.g. binding, gene expression, post-translational modification) between genes, gene products, or chemicals. Information added to Biofactoid, with author attribution, is shared on social media and public databases, such as Pathway Commons, where it can be discovered and analyzed in the context of a large and growing corpus of knowledge.

Please feel free to contact us if you have any questions.

With kind regards,

Sabrya.

Sabrya Carim, PhD

(she/her/hers)
Senior Editor, Nature Cell Biology
Nature Portfolio

Springer Nature
The Campus, 4 Crinan Street, London N1 9XW, UK
sabrya.carim@springernature.com
https://orcid.org/0000-0001-9485-1938

Click here if you would like to recommend Nature Cell Biology to your librarian
http://www.nature.com/subscriptions/recommend.html#forms

** Visit the Springer Nature Editorial and Publishing website at www.springernature.com/editorial-and-publishing-jobs for more information about our career opportunities. If you have any questions please click here.**

Open Access This Peer Review File is licensed under a Creative Commons Attribution 4.0 International License, which permits use, sharing, adaptation, distribution and reproduction in any medium or format, as long as you give appropriate credit to the original author(s) and the source, provide a link to the Creative Commons license, and indicate if changes were made.
In cases where reviewers are anonymous, credit should be given to 'Anonymous Referee' and the source.
The images or other third party material in this Peer Review File are included in the article's Creative Commons license, unless indicated otherwise in a credit line to the material. If material is not included in the article's Creative Commons license and your intended use is not permitted by statutory regulation or exceeds the permitted use, you will need to obtain permission directly from the copyright holder.
To view a copy of this license, visit https://creativecommons.org/licenses/by/4.0/

Point by point response to referees' comments for NCB-A60432-T

Reviewers' Comments:

Reviewer #1 (Remarks to the Author):

This article constitutes an exciting story, adding new insight about the still not well described regulation of the tissue specific rhythmic transcription by the circadian clock. Using ChIP-MS, authors decipher the tissue specific dynamic of the BMAL1-CLOCK complex around the time of its maximal activity (ZT 4-10). Based on this data, they identified Homeodomain factors interacting with BMAL1 and modulating its activity in a tissue specific manner. This is globally a very clear and interesting article based on high standard quality data. Nevertheless, a few questions need to be answered before publication.

- The main issue is the timing of this interaction/transcription repression by the Homeodomain factors. Authors described an interaction at the time of maximal BMAL1 activity while the interaction with the Homeodomain factors has a repressive effect. This sounds somehow puzzling. What is the dynamic of this interaction? Is there a competition with the binding of the CRY/PER complex, supposed to not interact with BMAL1/CLOCK at that time? What is happening at ZT19, when BMAL1 reaches its minimum of activity? Answering these questions is required to understand the mechanism involved.

We agree with the reviewer that the identification of homeodomain factors as repressors of CLOCK/BMAL1 activity at time of maximal transcriptional output may appear counterintuitive. However, when considering CLOCK/BMAL1 as “kamikaze” transcription factors, as previously proposed by the group of Ueli Schibler (PMID: 22981862), this observation becomes more consistent with a model in which these homeodomain factors act as modulators or buffers of transcriptional bursts. We have included this conceptual idea in the Discussion section of the manuscript.

Our data in the original manuscript indicated that the interaction between CLOCK/BMAL1 and these factors is dynamic and restricted to a defined time window during the transcriptional active phase: all three homeodomain factors co-immunoprecipitated with CLOCK/BMAL1 in the respective tissue at ZT4 and ZT7, but not at ZT10 (see Extended Data Fig. 3a).

To validate the absence of interaction at ZT10, we have now performed reciprocal immunoprecipitation experiments for each of the three homeodomain factors at this time point in the respective tissue. In all cases, BMAL1 and CLOCK were not detected as significant interactors, whereas previously reported interaction partners for PROX1 and HNF1B were identified, confirming the technical validity of the experiments (see new data in Extended Data Fig. 3d-f, also included below as Fig. 1). These findings indicate that the lack of CLOCK/BMAL1–homeodomain factor interactions at ZT10 is biologically meaningful and reflects the temporal specificity.

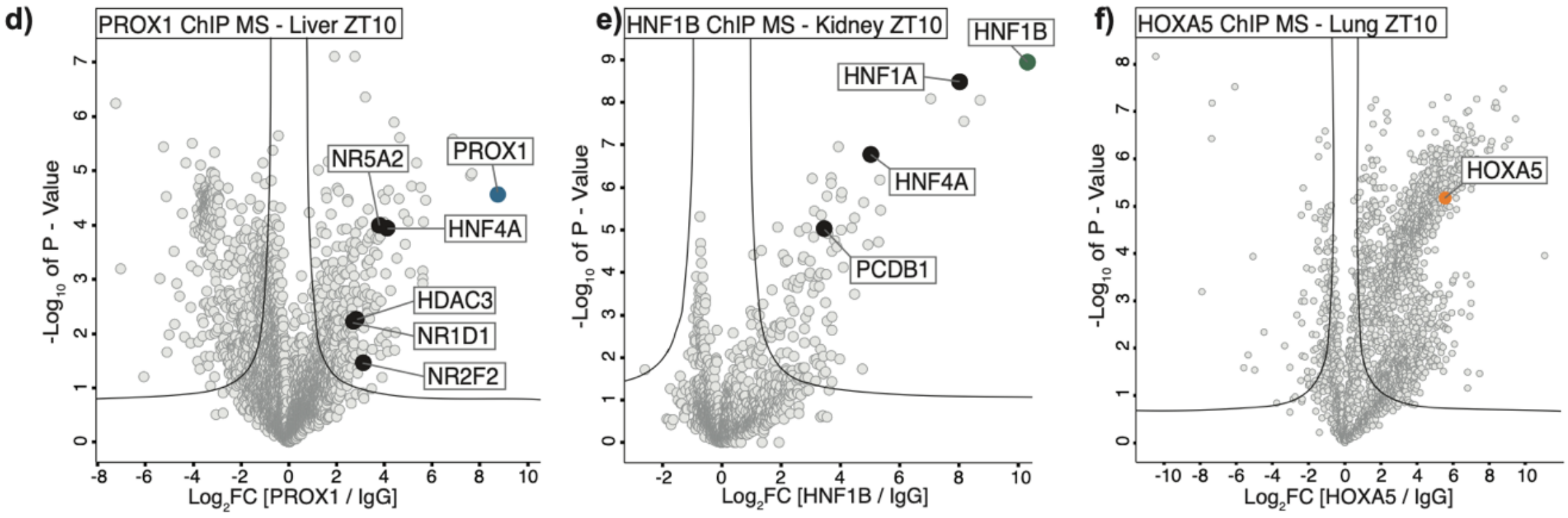

Fig. 1: Volcano plots representing the t-test results comparing ChIP-MS data at ZT10 with PROX1 and IgG in mouse liver (left), HNF1B and IgG in kidney (middle) and HOXA5 and IgG in lung (right). Known previously reported interactors are highlighted in the plots.

To more precisely define the interaction window, we performed new BMAL1 and CLOCK ChIP-MS at ZT1 across all three tissues. As expected for a late repression phase, we detected well-established components of the repression complex (CRY1, CRY2, PER1, PER2, CK1δ, and DBHS family members NONO, PSPC1, and SFPQ), but no homeodomain factors (see new data in Extended Data Fig. 3a, also included here as Fig. 2).

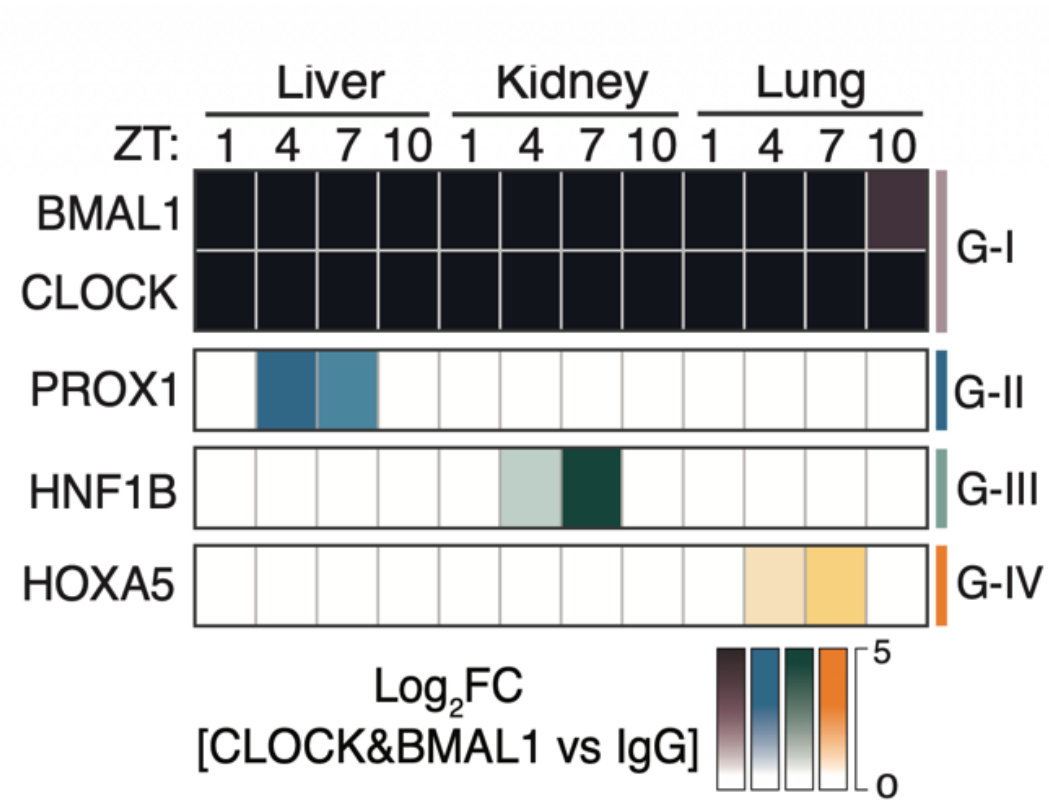

Fig. 2: Heatmap showing the mean abundance differences of BMAL1, CLOCK, PROX1, HNF1B and HOXA5 resulted from the student's t-test comparing BMAL1 and CLOCK vs IgG ChIP-MS in mouse liver, kidney and lung at ZT1, ZT4, ZT7 and ZT10. White indicates no statistical significance or no detection.

Taken together, these results further delineate the temporal dynamics of these interactions and demonstrate that, across all three tissues, PROX1, HNF1B and HOXA5 associate with CLOCK/BMAL1 within a define time window - after ZT1 and before ZT10 - corresponding to the phase of maximal CLOCK/BMAL1 transcription activity. This supports our model in which these factors function as modulators of transcriptional bursts. The new data have been incorporated into the revised manuscript (Fig. 2, Extended Data Fig. 2 and 3 and Supplementary Table 3).

While we cannot formally exclude the possibility that these homeodomain factors compete with the CRY/PER complex for binding to CLOCK/BMAL1, our data do not support this model. First, we detect CRY1 in immunoprecipitates that also contain the homeodomain factors at ZT4 and ZT7, indicating that their association with CLOCK/BMAL1 is not mutually exclusive. Second, at ZT10—when the homeodomain factors no longer interact with CLOCK/BMAL1—we also lack to detect components of the CRY/PER repression complex. Together, these observations argue against a simple competitive binding mechanism.

We did not include ZT19 in our analysis, as our primary objective was to identify co-regulators of CLOCK/BMAL1 at chromatin during the peak phase of transcriptional activity rather than during repression. Moreover, CLOCK/BMAL1 chromatin occupancy is reported to be very low or minimal at this time point (PMID: 22936566). That said, we agree with the reviewer that ZT19 represents an informative time point for studying the CRY/PER-containing repression complexes and for identifying potential novel repression regulators, and this could be an interesting direction for future investigation.

- What were the criteria for choosing the 3 Homeodoamain factors PROX1, HNF1B, and HOXA5? There is many transcription factors in the BMAL1 interactomes. What was the reason for choosing these ones?

We appreciate the reviewer's question regarding the rationale for selecting PROX1, HNF1B, and HOXA5 for further analysis among the many transcription factors identified in the BMAL1 interactomes.

Our choice was guided by two main considerations. First, all three factors have well-established roles in establishing and maintaining cell identity in their respective tissues, making them strong candidates for mediating tissue-specific transcriptional programs. This aligns with our central hypothesis that CLOCK/BMAL1 cooperates with tissue-specific transcription factors to shape context-dependent transcriptional outputs. Second, PROX1, HNF1B, and HOXA5 belong to the same transcription factor superfamily (homeodomain proteins), which provides a conceptual framework to test whether a related class of factors could share a common mode of interaction with CLOCK/BMAL1.

Importantly, these three factors also exhibited a highly similar and reproducible temporal interaction pattern with CLOCK/BMAL1—interacting at ZT4 and ZT7, but not at ZT10, and this pattern is further refined by our new data showing absence of interaction at ZT1. This consistent dynamic behavior across tissues further supports their selection for in-depth investigation.

These criteria are outlined in the revised manuscript (starting in line 155).

- Another issue relies on the statistical analysis. Analyzing independently the 2 datasets (WT and KO) with JTK cycle is not a proper analysis of differential rhythmicity (PMID: 29098954). Authors must use one of the specific methods recently developed for this specific purpose. On the same idea, figures 5C, 5D, and ED7 must be supported by statistical analysis.

We thank the reviewer for this important point and agree that analyzing the WT and KO datasets independently using JTK_CYCLE is not the most optimal method for assessing differential rhythmicity. To address this, we re-analyzed our time series data using DryR, a statistical framework more compatible to identify differential rhythmicity across conditions (PMID: 33452134).

As expected, the exact numbers vary slightly between methods, reflecting differences in underlying statistical approaches. Using DryR, we find that around 15% of detected genes are rhythmic in WT cells (compared to 10% with JTK), of which around 60% lose rhythmicity in the KO (compared to around 90% with JTK). Thus, while JTK_CYCLE appears to overestimate the proportion of arrhythmic genes in the PROX1 KO, the DryR analysis supports the same overall conclusion: a substantial fraction of rhythmic genes is affected by the loss of PROX1 in hepatocyte cells.

Importantly, DryR further strengthen our findings by classifying core clock genes as those maintaining rhythmicity and phase while changing in amplitude in PROX1 deleted cells. This provides a statistical framework supporting our original observations.

In response to the reviewer's comment, we have updated the manuscript accordingly including the new DryR analysis and replacing panels in Fig. 5e-g, Extended Data Fig. 7 and Supplementary Table 7.

- Related to these results, what is the overlap between the BMAL1-PROX1 cistrome and the differentially rhythmic genes? Considering the broad distribution of phases, some target genes appear likely indirect, an observation that appears conflicting with the global increase in amplitude of clock genes. What is the impact of Prox1 deletion on non-rhythmic BMAL1 targets?

This is an important point, and we have now performed additional analyses to clarify the relationship between the BMAL1–PROX1 cistrome and transcriptional outputs.

We find that, among all genes expressed in diHXY cells, 2,925 overlap with BMAL1/PROX1 ChIP-seq peaks identified in mouse liver at ZT7. Of these, DryR analysis classified 2,479 are arrhythmic and 446 genes (~15%) rhythmic in wildtype cells. This proportion is in good agreement with previous reports in mouse liver (PMID: 29300726) which estimate that only ~24% of BMAL1-bound genes are rhythmic, particularly given that our analysis compares liver ChIP-seq data with RNA-seq from a hepatocyte cell line. Importantly, among the rhythmic genes, a substantial fraction (259, ~58%) loses oscillation upon PROX1 deletion—a proportion comparable to that observed when considering all expressed genes. Consistent with this, PROX1 deletion has only a modest effect on non-rhythmic and BMAL1-PROX1 target genes; 2,141 out of the 2,479 arrhythmic genes (~86%) show no change in expression, while only a small fraction are upregulated (~5%) or downregulated (~8%) (see here Fig. 3).

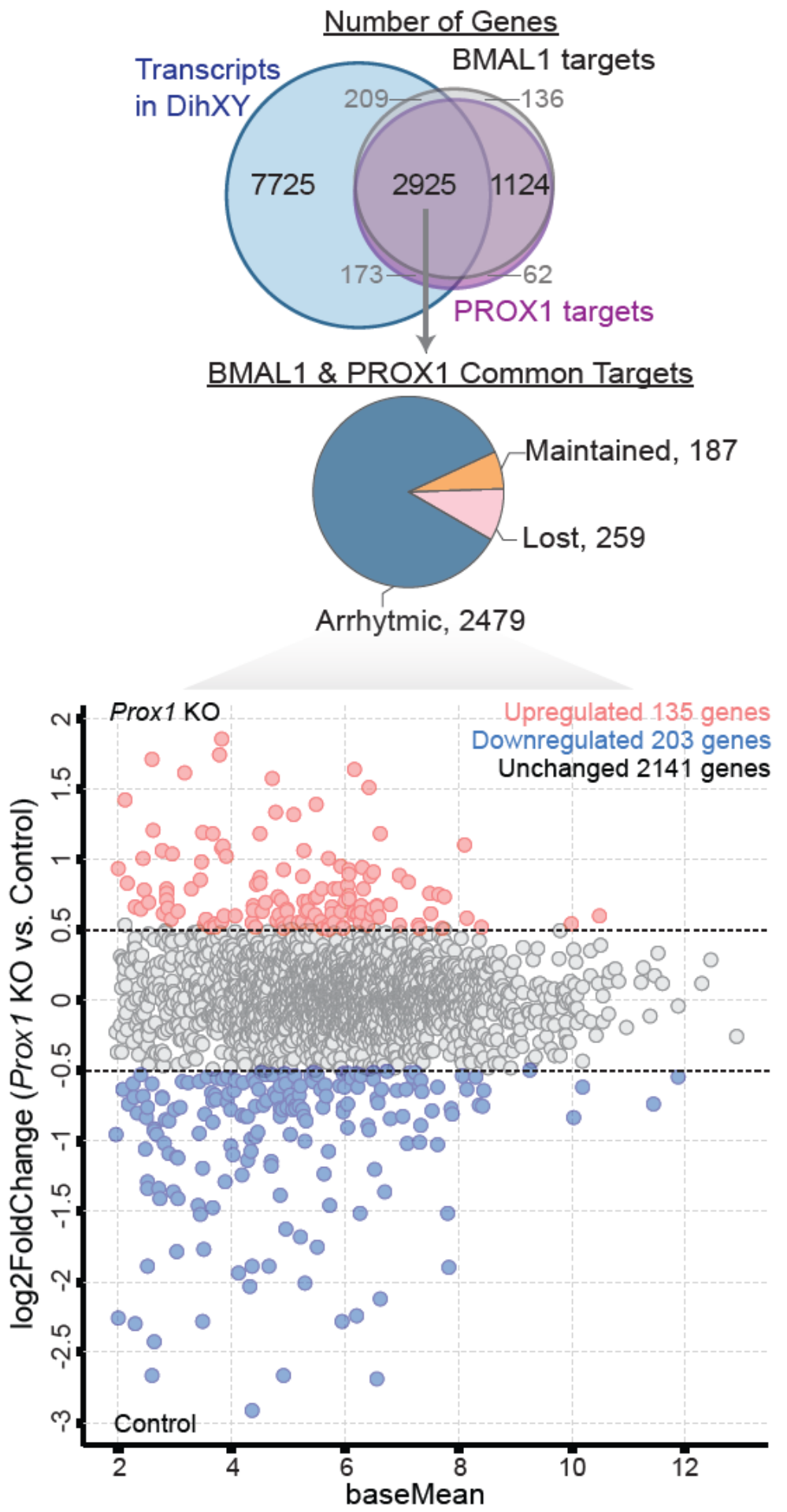

Fig. 3: Venn diagram representing the overlap of BMAL and PROX1 genomic sites in mouse liver at ZT7 with all transcripts from our RNAseq time series in DihXY (upper panel). Pie chart showing the DryR rhythmicity classification of common genes when comparing wildtype and Prox1 KO RNAseq time series (middle panel). MA plot displaying log2 fold changes between wildtype and Prox1 KO DihXY cells as a function of base mean expression for those genes arrhythmic in wildtype DihXY cells (lower panel).

Together, these data suggest that PROX1 primarily contributes to the maintenance of rhythmic transcription at active BMAL1 target genes, rather than broadly regulating transcription at all BMAL1-bound loci.

- Line 217, the sentence "this repressive function does not require DNA-binding" needs to be reformulated. Because there is no activation without DNA binding, there is also likely no repression.

We thank the reviewer for pointing this out and agree that the original wording was confusing. Our intention was not to suggest that repression occurs independently of DNA binding per se, but rather that the interaction between these factors and CLOCK/BMAL1 does not require binding to an additional, distinct DNA motif, nor is it mediated indirectly through DNA. We have revised this statement in the manuscript to clarify this point.

Reviewer #1 (Remarks on Protocol(s)):

No specific comment on the method except for the statistical analysis section.

Reviewer #2 (Remarks to the Author):

Peer Review for CLOCK/BMAL1 interactome uncovers homeodomain factors as tissue regulators
Aygenli et al. use high-throughput mass spectrometry (MS-) and ChIP-based approaches to detect novel protein interactors of the core circadian BMAL1:CLOCK complex. This was extended to various tissues to detect tissue-specific interactions with homeodomain transcription factors namely, PROX1, HNF1B, and HOXA5 in the mouse liver, kidney, and lung, respectively. The high-throughput analysis alone can already serve as a rich resource for further studies on novel BMAL1:CLOCK interactors. Validation of the detected tissue-specific interactors in PROX1, HNF1B, and HOXA5, is comprehensive: protein-protein interaction is confirmed via IP, genome co-occupancy via ChIP, transcriptional repression via transactivation assays. Interestingly, AlphaFold predicts a common interaction interface occurring between the respective homeodomains and the BMAL1 TAD with the key residues validated by IP and transactivation assays. Finally, genetic knockout affecting amplitude and resulting in tissue-specific loss of rhythmicity rounds out the analysis of these novel BMAL1:CLOCK interactors and repressors, confirming their importance in supporting tissue-specific rhythmicity. While homeodomain TFs typically play roles in development, the authors propose that BMAL1:CLOCK repression by these homeodomain TFs may serve as a mechanism to buffer BMAL1:CLOCK activity, and thereby complex abundance during the activation phase. Overall, this work represents a significant conceptual advance and is of interest for a broad readership beyond the circadian and developmetal biology fields.

We do not have any major points requiring revision; the minor points listed below are optional. Additional requests could make the manuscript slightly different, but not necessarily better. We therefore feel that major revision requests would place the editors in a difficult position, as they would either need to overrule the reviewer or convey revision requests to the authors that would unnecessarily consume the time and resources of the laboratories involved.

We are extremely thankful to the reviewers for the positive evaluation of our work and for noting that no major revisions are required. We also appreciate the flexibility offered regarding the minor points. While these points were considered optional, we found them to be constructive and valuable for improving the clarity and robustness of our study. We therefore addressed all of them and incorporated the corresponding revisions into the manuscript.

Minor points

1. The authors may consider performing a CoIP with both BMAL1 and CLOCK and testing for the interaction with PROX1, HNF1B, and HOXA5. Given that the authors also propose that the PAS core domain is important for the interaction with the homeodomain TF, a complete BMAL1:CLOCK complex may result in a more definitive signal showing protein-protein interaction.

We thank the reviewers for this insightful suggestion and agree with the rationale that assessing interactions with the full CLOCK/BMAL1 complex could provide additional clarity. We therefore performed the suggested co-immunoprecipitation experiments. As expected, when co-expressed, PROX1 efficiently co-immunoprecipitates both BMAL1 and CLOCK. However, in the absence of BMAL1, PROX1 fails to co-immunoprecipitate CLOCK (see plot below), indicating that the interaction with CLOCK is indirect and likely mediated through BMAL1. These results further support a model in which BMAL1 serves as the primary interaction platform for homeodomain transcription factors within the CLOCK/BMAL1 complex. The new data have been incorporated into the revised manuscript (Extended Data Fig. 3g).

2. The authors may consider including Extended Data Figs. 6 A and B to Fig. 4 as Fig 4 I and J as this is an interesting point to consider: while mutation of E614 and L619 to alanine does not affect protein-protein interaction, they do affect the repressive capacities of PROX1, HNF1B, and HOXA5 (and CRY1 as well, the authors might have missed adding those asterisks in the figure).

Following the reviewers' recommendation we have incorporated the previous Extended Data Fig. 6a and b into Fig. 4i,j. In addition, we have corrected the figure to include the missing asterisks in the CRY1 condition and added to the figure legend the corresponding statistical significance values.

3. The authors may provide a comment explaining the 10-fold decrease in transactivation between Fig 4F and 4H.

We acknowledge the reviewers' point and provide the following clarification. In both panels, transcriptional activity is reported as relative luciferase units, calculated by normalizing firefly luciferase activity to the Renilla luciferase co-transfection control. This normalization is essential to account for experimental variability, including differences in transfection efficiency, cell lysis, and pipetting. Consequently, absolute normalized values can vary between independent experiments, largely due to fluctuations in Renilla luciferase reads. Thus, direct comparison of absolute values across panels (for example in Fig. 4f and 4h), which derive from separate experiments, can be misleading. Instead, the most relevant parameter is the relative effect across conditions within each experiment, which is highly consistent in all our experiments: co-expression of PROX1, HNF1B, or HOXA5 reproducibly reduces transcriptional activity to approximately 50% of the BMAL1/CLOCK-alone condition.

4. In Figures 5 A and B, the authors may normalize the relative mRNA levels to the control to more clearly show the fold-level increase in expression upon PROX1 or HNF1B KO.

We appreciate this suggestion however we chose not to normalize the data to control conditions for two reasons. First, to avoid double normalization and second because normalization to the control condition results in a substantial expansion of the dynamic range of the y-axis which reduces the clarity of the plotted data. We consider that the current representation provides a more balanced visualization of the expression changes while allowing clear comparison between conditions.

5. In line 356, the formatting for citation 42 seems to be incorrect.

This formatting issue has been corrected in the revised manuscript.

6. In general, the citation formatting throughout the text is slightly incorrect as it includes a space between the text and the citation. As an example, in line 5, "outputs 1-3" should be reformatted to "outputs1-3". All citations should be reformatted appropriately.

We thank the reviewer for noting this issue. Citation formatting has been standardized throughout the manuscript.

Fidel Serrano and Michael Brunner

Reviewer #3 (Remarks to the Author):

The authors map the BMAL1 and CLOCK interactomes across three mouse tissues (liver, kidney, lung) at three time points (ZT4, ZT7, ZT10) and identify 1,463 high-confidence interactors, including tissue-specific interacting ttranscription factors (TFs). Among these were three homeodomain TFs (PROX1, HNF1B, HOXA5), which were shown to directly bind and co-occupy most BMAL1 genomic sites. In addition, AlphaFold-based structural predictions followed by mutational analyses suggested that homeodomain (HD) factors repress BMAL1/CLOCK activity.While the study is conceptually interesting, there are major concerns regarding experimental design, data interpretation, and terminology that are problematic for this study. For example, In terms of the timing, the authors only study three closely spaced time points. Moreover, while BMAL1 DNA-binding activity peaks in liver around ZT7, this is majorly delayed from the protein peak around ZT16-20, and it is not clear whether this unusual delay is also seen in lung and kidney. Moreover, the authors focus on the 3 homeodomain TFs without clear rationale, and suggest that these factors couple the clock to organ identity without experimental evidence.

Major Comments

1, The authors identified 1,436 interactors, and state on line 147 that 5-10% of these are transcriptional regulators. Of these they focused on three homeodomain TFs, which they justify based on the annotation term "developmental processes" in Fig. 2F. However, it is not clear how many other TFs are present in the interactors belonging to this patway, and why these specific TFs were selected.

To address this point, we provide here specific numbers: within the “developmental process” category we identified 40 transcription factors, of which 22 are tissue specific. Notably, 5 of these belong to the homeobox transcription factor family, among which PROX1, HNF1B, and HOXA5 have well-established developmental roles in their respective tissue. This served as a primary selection criterion, consistent with our hypothesis that CLOCK/BMAL1 engages tissue-specific transcription factors in a time-dependent manner to regulate tissue-specific transcriptional programs. As a second criterion, these factors exhibited a highly similar and reproducible temporal interaction pattern with CLOCK/BMAL1—interacting at ZT4 and ZT7, but not at ZT10 (further supported by new reciprocal ChIP-MS data discussed below in point 5 and in point 1 of Reviewer 1) nor at ZT1(an additional time point included in the revision to address point 1 from Reviewer 1). All new data are integrated in the revised Fig. 2, Extended Data Fig. 2 and Extended Data Fig. 3).

2. The authors selected ZT7 as the peak of BMAL1/CLOCK chromatin binding, based on the data in reference 12. Other groups have found similar results, however this peak of chromatin binding occurs many hours after the peak of BMAL mRNA and protein expression. This phenomenon is poorly understood, and to this reviewer’s knowledge has not been shown in kidney or lung. These details are highly relevant to the present study, especially sincd tissue-specific phase differences have been reported for peripheral clocks. The authors should provide supporting data demonstrating that BMAL1/CLOCK DNA binding indeed peaks at ZT7 in kidney and lung.

We appreciate the reviewer’s point regarding the temporal relationship between BMAL1/CLOCK expression and chromatin binding, as well as the potential for tissue-specific phase differences.

In mouse liver, it is well established that BMAL1 and CLOCK mRNA and total protein levels do not strictly coincide with chromatin binding. However, previous studies (PMID: 27818260), together with recent work (PMID: 41519126) as well as unpublished data from our group profiling nuclear protein abundance across the circadian cycle, reveal that nuclear BMAL1 levels are rhythmic and strongly correlate with its chromatin occupancy, peaking around ZT7 (see below our answer to point 6 and associated Fig.2).

While similar comprehensive nuclear proteome studies have not yet been performed in kidney and lung, published reports have assessed BMAL1 and CLOCK occupancy at canonical clock-controlled gene loci across the circadian cycle in these tissues. These data indicate that BMAL1 and CLOCK chromatin binding in both kidney and lung follows a temporal profile highly similar to that observed in the liver, with a peak around ZT7 (see figure below showing the panels and figure legends shown in this reports).

Together, these observations, along with our findings of comparable dynamics in the size of the CLOCK/BMAL1 interaction networks across tissues (Extended Data Fig. 2b), support the idea of a conserved phase of maximal chromatin binding in these peripheral tissues.

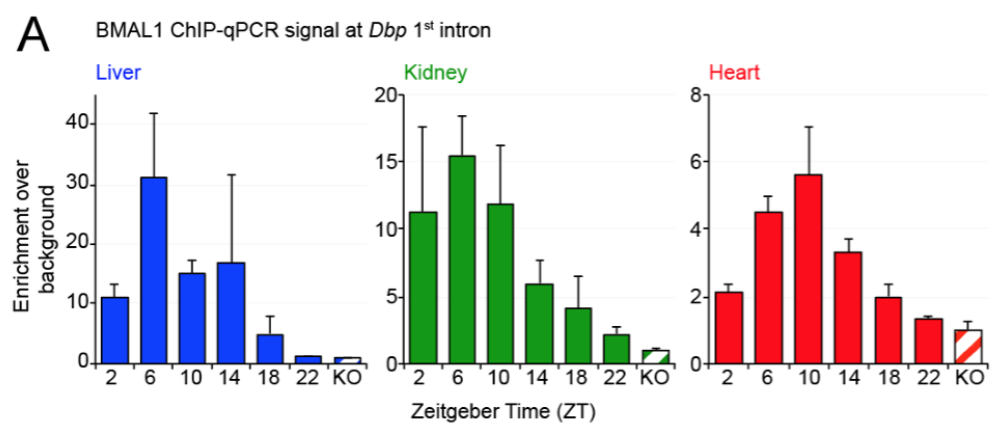

http://www.genesdev.org/cgi/doi/10.1101/gad.322198.118.
Beytebiere, Menet, 2018, GenesDev

Supp. Figure 1A: BMAL1 ChIP-qPCR signal at Dbp 1st intron over the course of the day in the mouse liver (blue), kidney (green), and heart (red). Tissues were collected in wild-type mice at ZT2, ZT6, ZT10, ZT14, ZT18, and ZT22, and in Bmal1 knockout mice at ZT6 for negative control. ChIP-qPCR signal normalized to the input signal corresponds to the average ± s.e.m. of 3 biological replicates, and the ratio ChIP/input was set to 1 for the Bmal1 knockout mice samples.

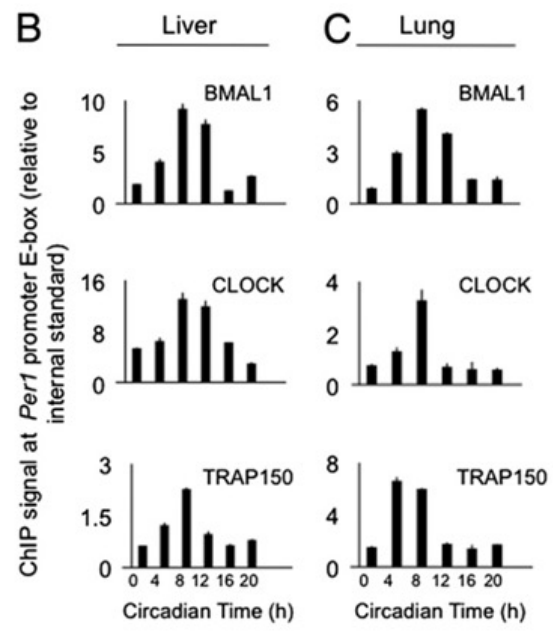

https://doi.org/10.1073/pnas.1305980110
Laura Lande-Diner, Cyril Boyault, Jin Young Kim, and Charles J. Weitz, PNAS, 2013

Figure 2B: Synchronous circadian cycle of BMAL1, CLOCK, and TRAP150 at Per1 E-box site. Shown are ChIP assays from mouse liver (B) and lung (C) obtained across a circadian cycle performed with antibodies indicated at the upper right corner of each panel. Background was determined from an arbitrary internal control gene and was set to a value of 1.

3. Related to this, in Line 149, the statement that “spatiotemporal analysis demonstrates that the chromatin-associated protein interaction landscape of CLOCK/BMAL1 is shaped predominantly by tissue context, rather than circadian time” is an overstatement. With only three time points sampled within a relatively narrow window, it is difficult to conclude that circadian time plays a minor role. Additional time points or a more cautious interpretation would be warranted. We thank the reviewer for the suggestion and agree that the statement was too broad. We have therefore revised the text to more accurately reflect the data obtained in this study, highlighted in blue (line 149) in the revised manuscript.

4. Moreover, multiple BMAL1 mouse liver ChIP-seq datasets across tissues are already available. The authors should compare their own ChIP-seq datasets with these, for example in liver (PMID: 30804225, 22936566), to strengthen confidence in the conclusions.

As noted by the reviewer, this comparison was already included in the original version of the manuscript. Specifically, we compared our BMAL1 ChIP-seq peaks overlapping with previously published dataset (PMID: 30804225) and reported those with high confidence, that also showed a high degree of overlap This analysis is described in the Supplementary Methods section (highlighted in the revised manuscript).

5. Figure 1C and Table S1 indicate that BMAL1 and CLOCK share 379 interactors in liver, including PROX1. However, in Figure 3B, PROX1 ChIP fails to detect CLOCK, leading the authors to propose that PROX1 binds the BMAL1 transactivation domain (TAD) without interacting with CLOCK. However, since CLOCK is bound to BMAL1, and PROX1 is present in CLOCK ChIP-MS (Table S1), it is why CLOCK is not present in the PROX1 ChIP-MS via the presence of BMAL1.

This is an important point, and we have carefully considered this apparent discrepancy. Our data consistently support a model in which PROX1 associates with the CLOCK/BMAL1 complex primarily through direct interaction with BMAL1, rather than CLOCK.

First, our co-immunoprecipitation experiments in cell lines indicate that BMAL1, but not CLOCK alone, can efficiently immunoprecipitate PROX1 (as well as HNF1B and HOXA5) (Fig. 3C), suggesting that BMAL1 is required for this interaction. To further address the reviewer's concern, we performed additional experiments in which BMAL1 and CLOCK were co-expressed. Under these conditions, both BMAL1 and CLOCK are detected in PROX1 immunoprecipitates. However, in the absence of BMAL1, PROX1 fails to immunoprecipitate CLOCK, indicating that the association with CLOCK is indirect and mediated via BMAL1. These new data are now included in the revised manuscript (Extended Data Fig. 3g).

Second, the apparent asymmetry observed in the ChIP-MS datasets (namely, detection of PROX1 in CLOCK ChIP-MS, but not CLOCK in PROX1 ChIP-MS) can be explained by differences in complex composition and stoichiometry in vivo. PROX1, HNF1B, and HOXA5 are transcription factors that also function in multiple regulatory complexes beyond CLOCK/BMAL1, as supported by previous studies (PMID:28916805, PMID:29175243). Consequently, PROX1 immunoprecipitates likely enrich for its direct interaction partners across diverse chromatin contexts including BMAL1 but may not efficiently capture indirect interactors such as CLOCK. Consistent with this, our PROX1 ChIP-MS data reveal a set of known interactors that likely representing additional PROX1-containing complexes at chromatin sites distinct from those bound by CLOCK/BMAL1. Notably, a greater number of these interactors are detected in our new data at ZT10 (see Figure 1 below and revised Extended Data Fig.3d and Supplementary Table 5), a time point at which PROX1 no longer interacts with BMAL1. Together, our findings further support the idea that PROX1 participates in alternative complexes independent of CLOCK/BMAL1, in agreement with previous studies.

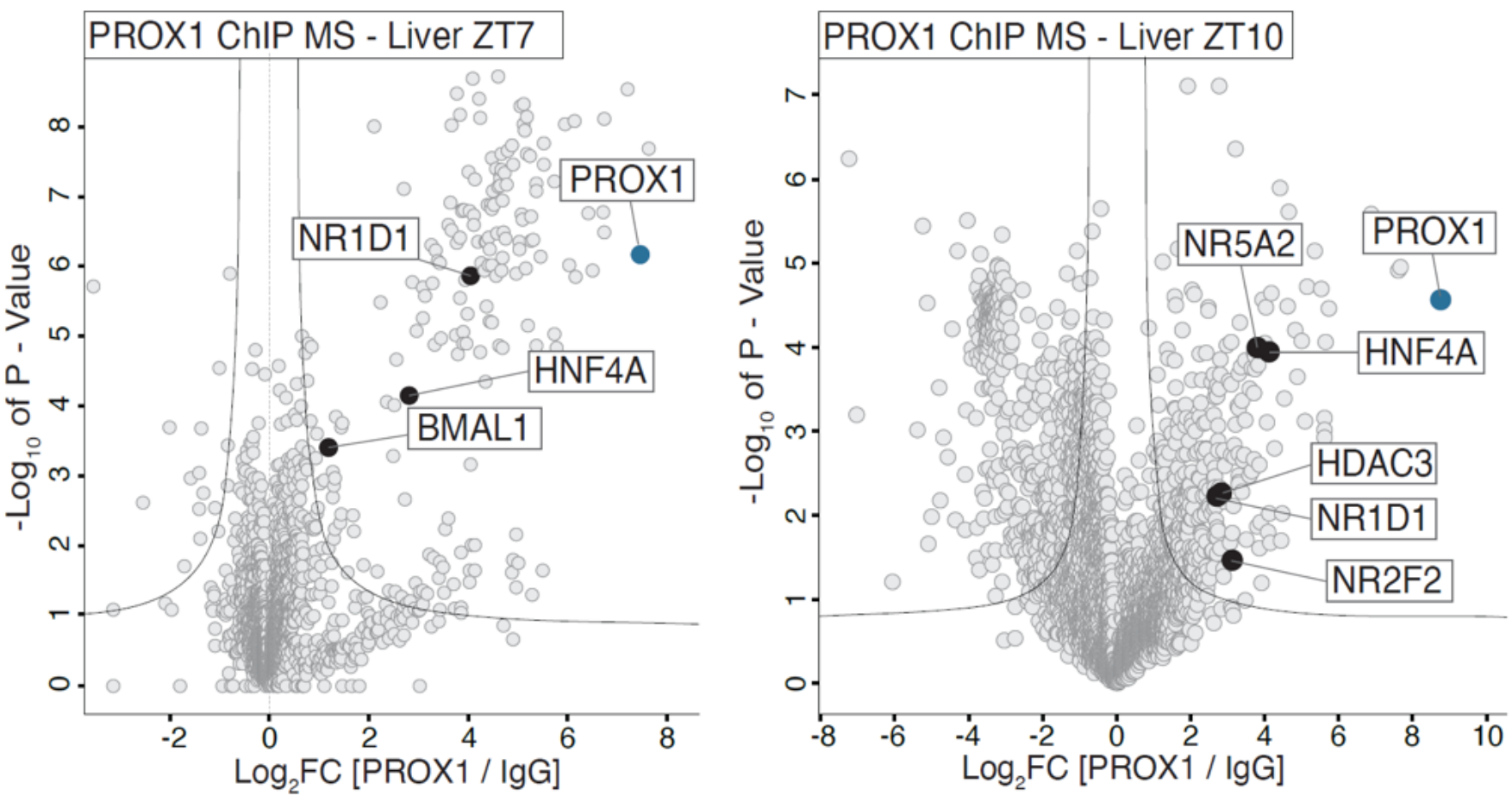

Fig. 1: Volcano plots representing the t-test results comparing PROX1 and IgG ChIP-MS in mouse liver at ZT7 (left) and ZT10 (right). Known previously reported interactors are highlighted in the plots. These data are included in the revised Fig. 3b and Extended Data Fig 3d).

In contrast, a large proportion of BMAL1 chromatin sites (>80%) are co-occupied by PROX1 (or HNF1B), indicating that these factors are frequently present within CLOCK/BMAL1 complexes. Consequently, PROX1 is detected in CLOCK ChIP-MS as part of these complexes, even if the interaction with CLOCK itself is indirect. A similar logic applies to known indirect interactors such as CIPC, which is detected in BMAL1 ChIP-MS despite binding directly to CLOCK (PMID: 17310242).

Together, these data further confirm our overall results and support a model in which PROX1 interacts directly with BMAL1 and is incorporated into CLOCK-containing complexes through this interaction.

6. The authors do not measure circadian oscillation of BMAL1 In mouse liver, but prior studies have shown that despite the large amplitude of Bmal1 mRNA, BMAL1 protein circadian oscillation exhibits a moderate amplitude in liver. However, the temporal expression profile of PROX1 is not addressed in liver (or the other tissues). It would be important to determine whether PROX1 protein levels or its interaction with BMAL1 are also rhythmic. This information is critical for understanding whether PROX1-mediated repression is circadian phase-dependent.

This is an important point. Although BMAL1 protein levels in whole liver show only modest oscillations, previous studies (PMID:27818260), unpublished data from our laboratory and a recent report published after submission of our manuscript (PMID: 41519126)—have demonstrated that BMAL1 levels in the nuclear compartment are strongly rhythmic, peaking around ZT7. Notably, these nuclear oscillations correlate well with its chromatin binding profile. In contrast, PROX1 nuclear protein levels do not show significant oscillation across the time points examined (see Fig. 2 below). Together, this supports the idea that the dynamic interactions of CLOCK/BMAL1 are not simply driven by changes in protein abundance, but reflect regulated, time-dependent interactions.

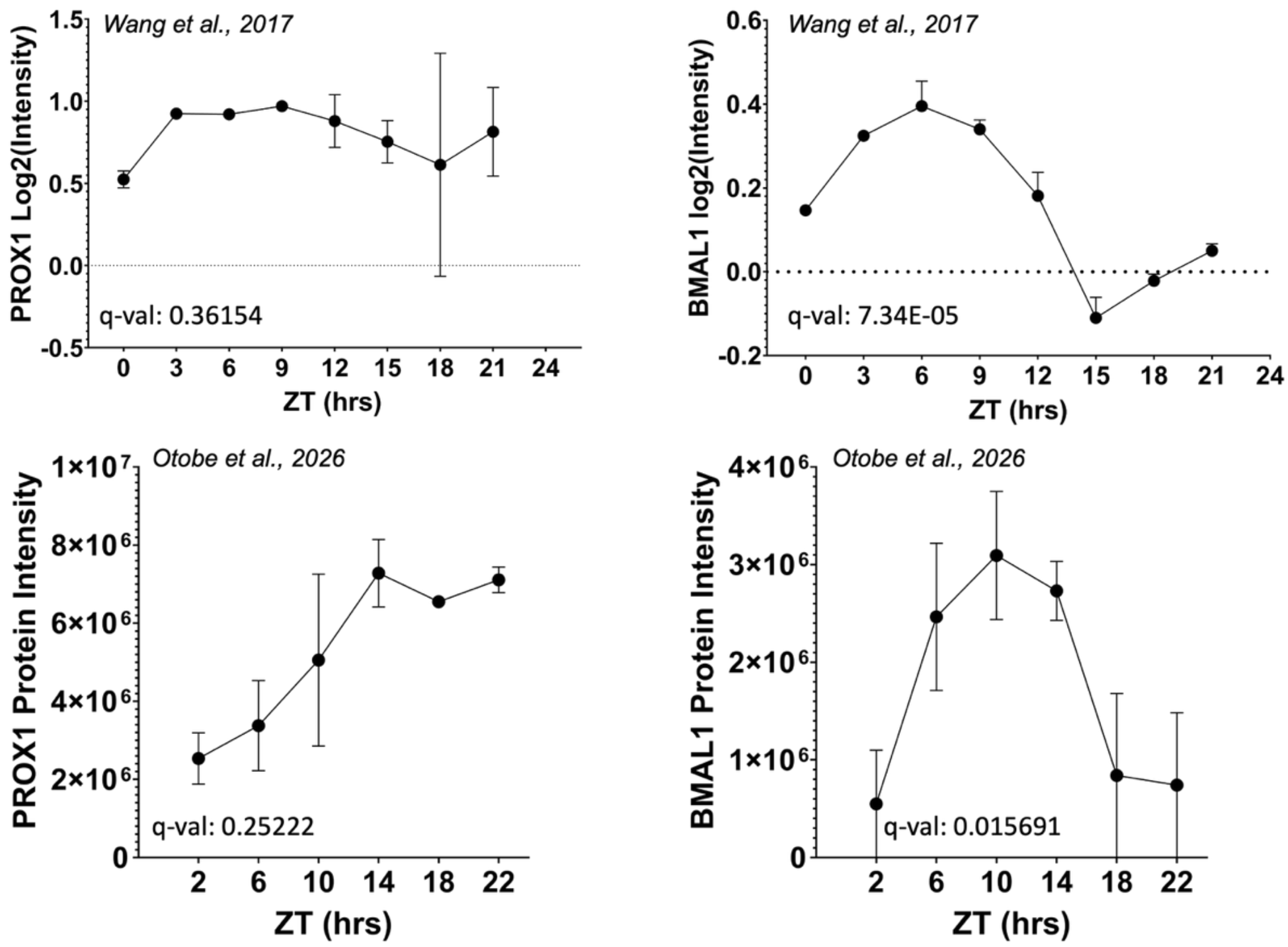

Fig. 2: Temporal profiles of nuclear protein intensity for PROX1(left) and BMAL1 (right) extracted from the data in PMID:27818260 (upper panels) and PMID: 41519126 (lower panels), with the q-value reported for each time-series data.

7. The authors use AlphaFold to predict the interaction interface and identify the BMAL1 TAD as critical for binding homeodomain factors. While informative, the subsequent functional analyses rely on overexpression-based mutagenesis. Given the central role of this interaction in the proposed model, endogenous mutation (e.g., CRISPR-mediated editing) would provide stronger and more physiologically relevant evidence for the role of the BMAL1 TAD in homeodomain factor–mediated repression.

We thank the reviewer for this suggestion. In the present study, we used overexpression-based mutagenesis as a well-established and tractable system to directly test the functional relevance of the predicted interaction interface. These experiments allowed us to specifically interrogate the

contribution of the BMAL1 TAD to both protein–protein interactions and transcriptional repression in a controlled manner. and agree that endogenous perturbation approaches, such as CRISPR-mediated editing. We agree that extending these findings to endogenous settings would be valuable. However, such experiments are technically demanding and were beyond the scope of the current study. We have therefore tempered our conclusions accordingly and now explicitly acknowledge this limitation in the revised manuscript.

8. In Figure 5E, Prox1 knockout in diHXY cells retains rhythmicity in 98 genes, including core clock genes, but disrupts rhythmicity in 1,171 genes. It would be informative to assess how BMAL1 expression and/or activity is affected following Prox1 deletion. Additionally, the authors should clarify how loss of rhythmicity in a large gene set occurs despite preserved core clock gene oscillations, and whether this reflects impaired clock output rather than clock function.

We appreciate this comment and agree that it raises an important distinction between core clock function and circadian output. Our data support a model in which PROX1 primarily modulates clock output rather than the core clock machinery itself. Consistent with this, although a large number of genes lose rhythmicity upon Prox1 deletion, core clock genes remain rhythmic, indicating that the central oscillator is preserved while downstream transcriptional programs are affected.

To further address this point, we quantified BMAL1 protein levels in PROX1- and HNF1B-deficient cell lines (see Fig. 3 below, also revised Extended Data Fig. 7c) and did not observe any significant changes. These results indicate that the loss of rhythmic gene expression is not due to altered BMAL1 protein abundance but rather reflects changes in circadian transcriptional output driven by the modulatory activity of the homeodomain transcription factors.

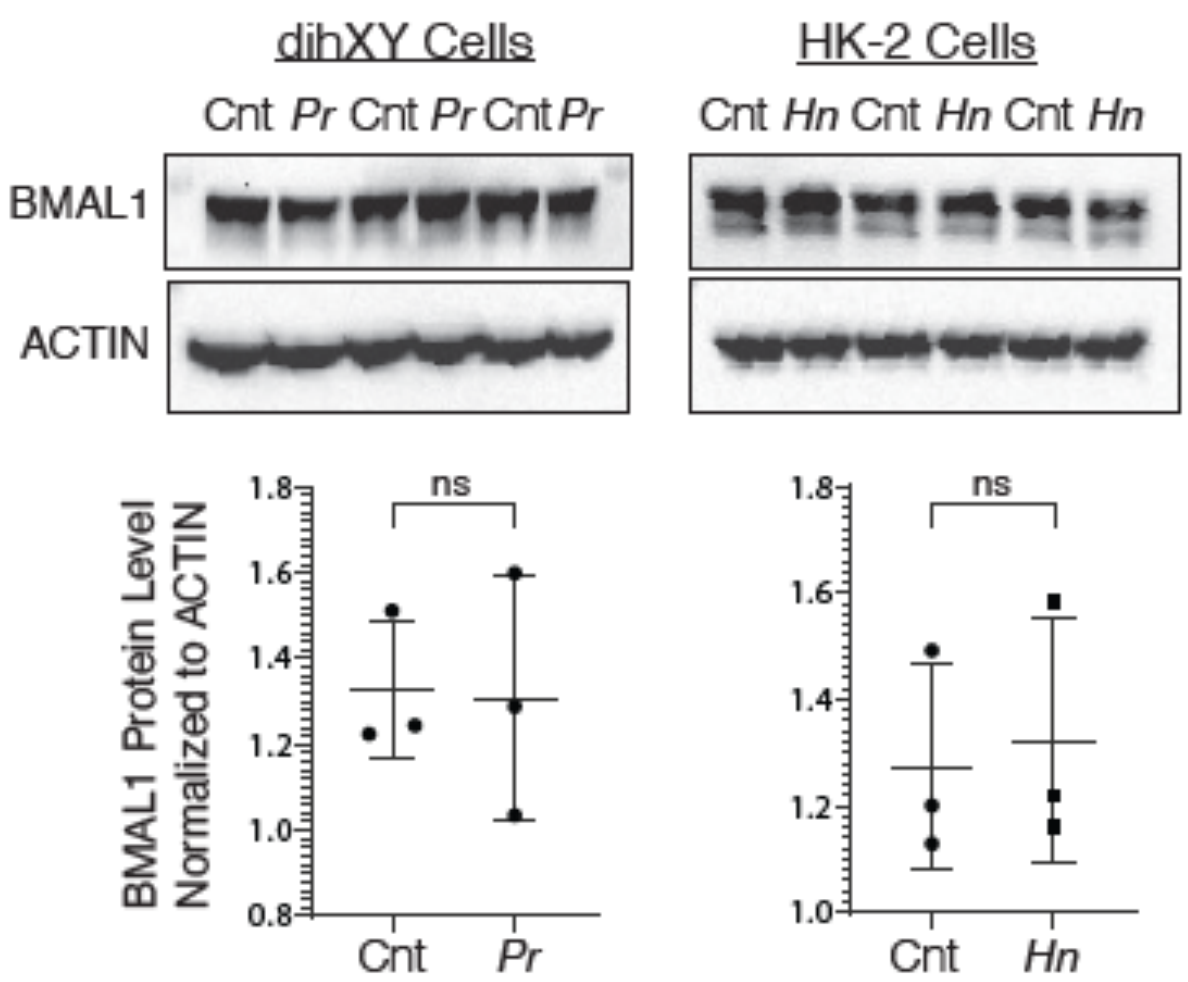

Fig. 3: Immunoblots showing endogenous protein levels of BMAL1 in Control (Cnt) and CRISPR/Cas9 mediated individual PROX1 (Pr) in DihXY mouse hepatocytes (left) and HNF1B (Hn) knockout in HK-2 human kidney cells (right). ACTIN was used as a loading control. Bar plots with the quantification of BMAL1 protein levels normalized to ACTIN, presented as mean ± s.d. (n = 3), with two-tailed Student's t-test, ns: no significant.

Minor Comments

1. In Line 169, the term "reverse ChIP/MS" is misleading, as it could be interpreted as locus-centric chromatin pulldown followed by proteomics. In this context, "reciprocal ChIP-MS" would be a more accurate and standard term.

We appreciate the reviewer for pointing this out. We have therefore replaced it with "reciprocal ChIP-MS" in the manuscript to ensure clarity and consistency with standard terminology.

2. Protein data should be made available to reviewers via a public database such as ProteomeXchange.

All proteomics data from the original manuscript was deposited in ProteomeXchange, where we now included all new proteomics data generated during the revision. Data are available as described in the Data Availability section.

Point-by-point response to any remaining reviewer comments for NCB-A60432A

Reviewer #1 (Remarks to the Author):

Authors have adequately and convincingly addressed all reviewers' queries. Nevertheless, the question regarding the overlap of BMAL1-PROX1 cistromes is of importance and this should be included in the manuscript, at least as a supplementary figure.

We thank the reviewer for the positive assessment of our revised manuscript. We agree that the transcriptional changes observed upon *Prox1* deletion at genes where BMAL1 and PROX1 colocalize are relevant to the study. Following the reviewer's suggestion, we have included these data as a new supplementary figure, Fig. S9. A description of the analysis and the relevance of these data have also been added to the Results section of the revised manuscript.

Reviewer #2

Comment:
The authors have adequately addressed all points raised by us.

We thank Reviewer #2 for their positive evaluation of our revised manuscript.

Reviewer #4

Comment:
The revised manuscript is responsive, and the concerns raised by the reviewers have been addressed.

We are grateful to Reviewer #4 for their thoughtful assessment of Reviewer #3's points and appreciate their involvement in the review process at this late stage.
