# Supplementary material for: CLOCK/BMAL1 interactome uncovers homeodomain factors as tissue regulators
Source: Nat Cell Biol. 2026 Aug 6;28(9):1890–900. doi: 10.1038/s41556-026-02041-4 (PMC13561871; doi:10.1038/s41556-026-02041-4)

Unprocessed Immunoblot images relating to Figure 3c

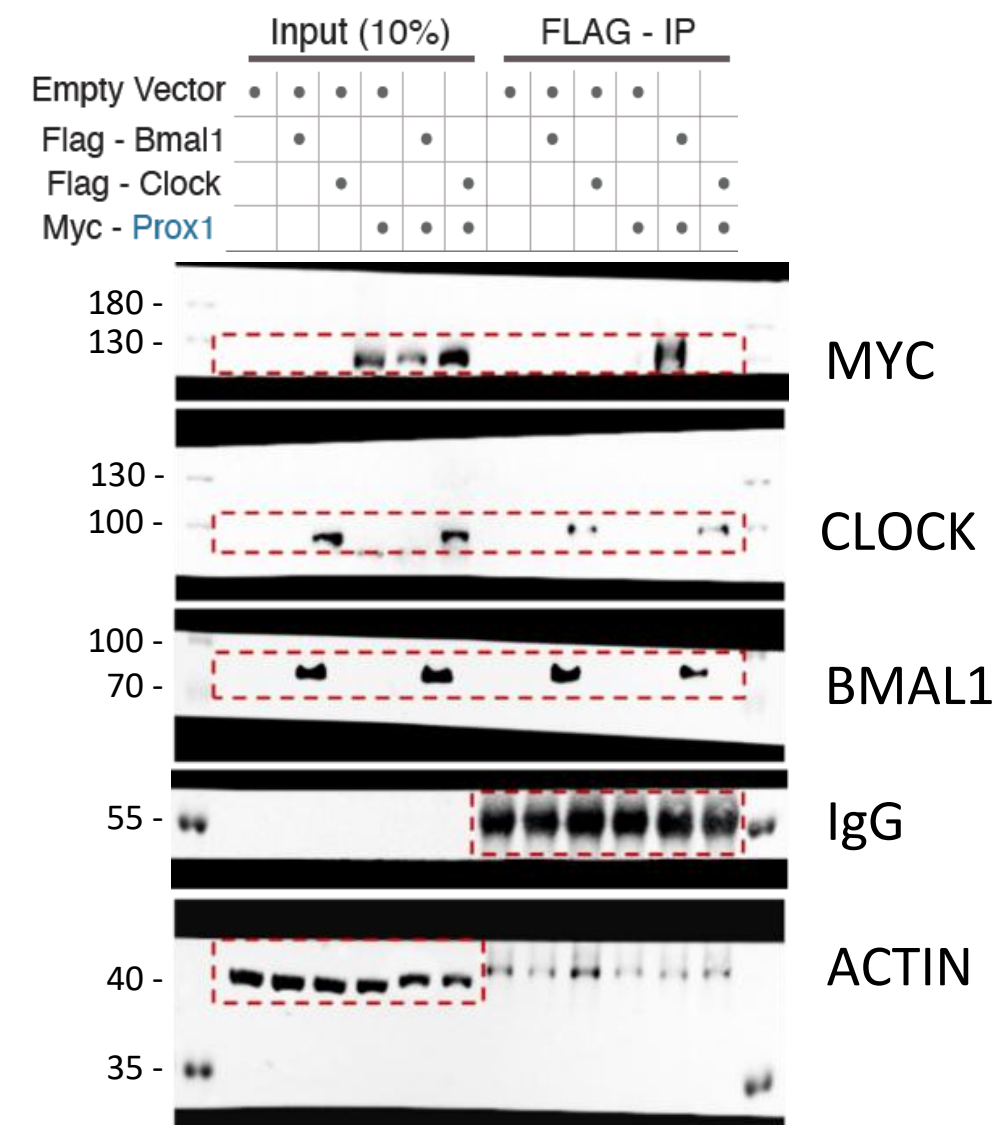

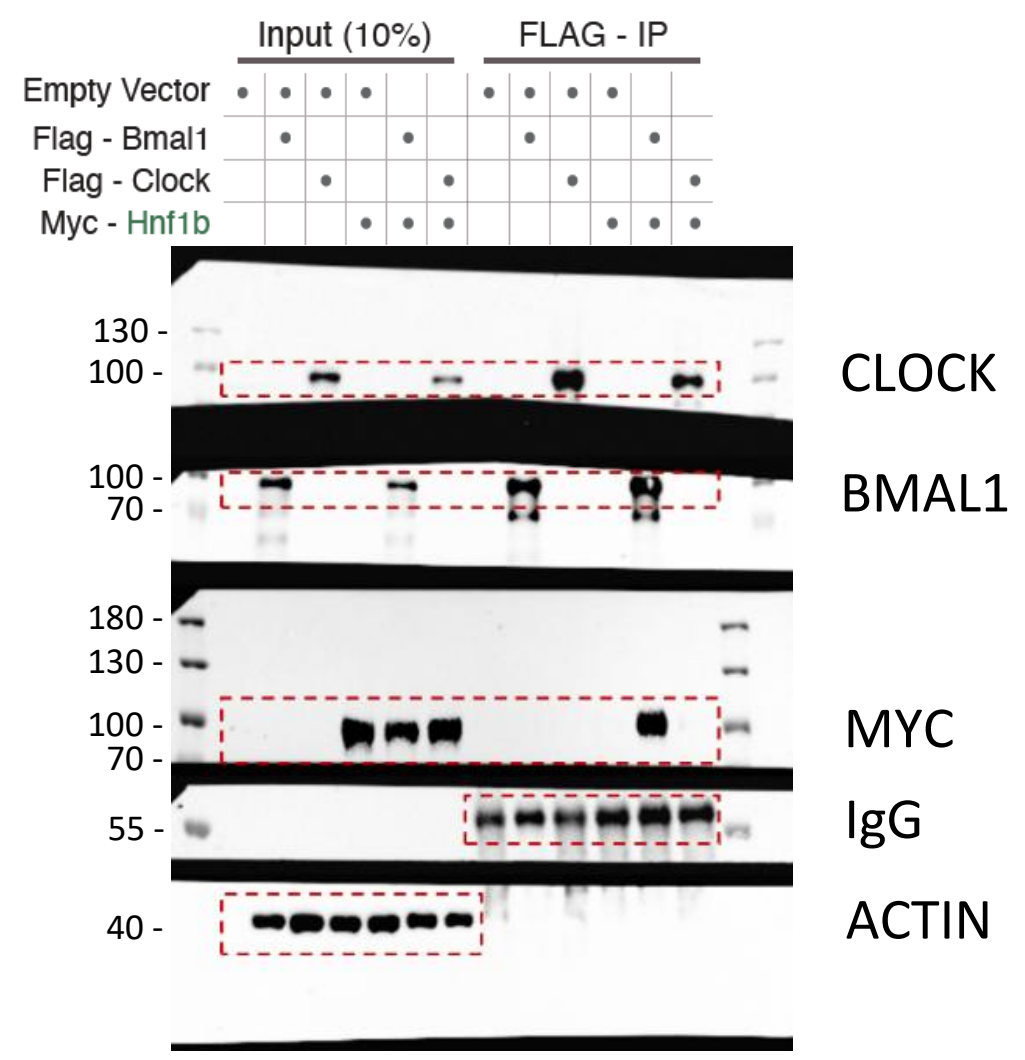

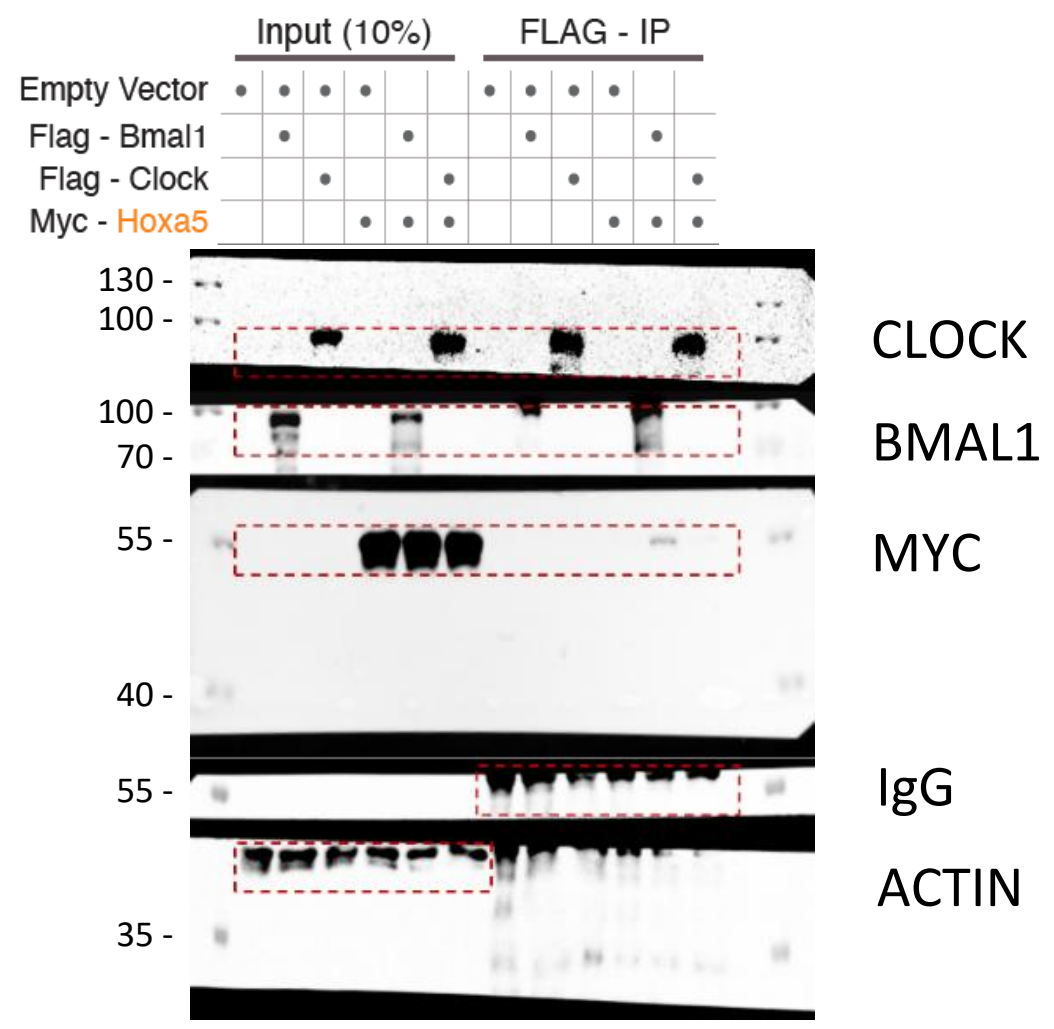

Unprocessed Immunoblot images relating to Figure 4j

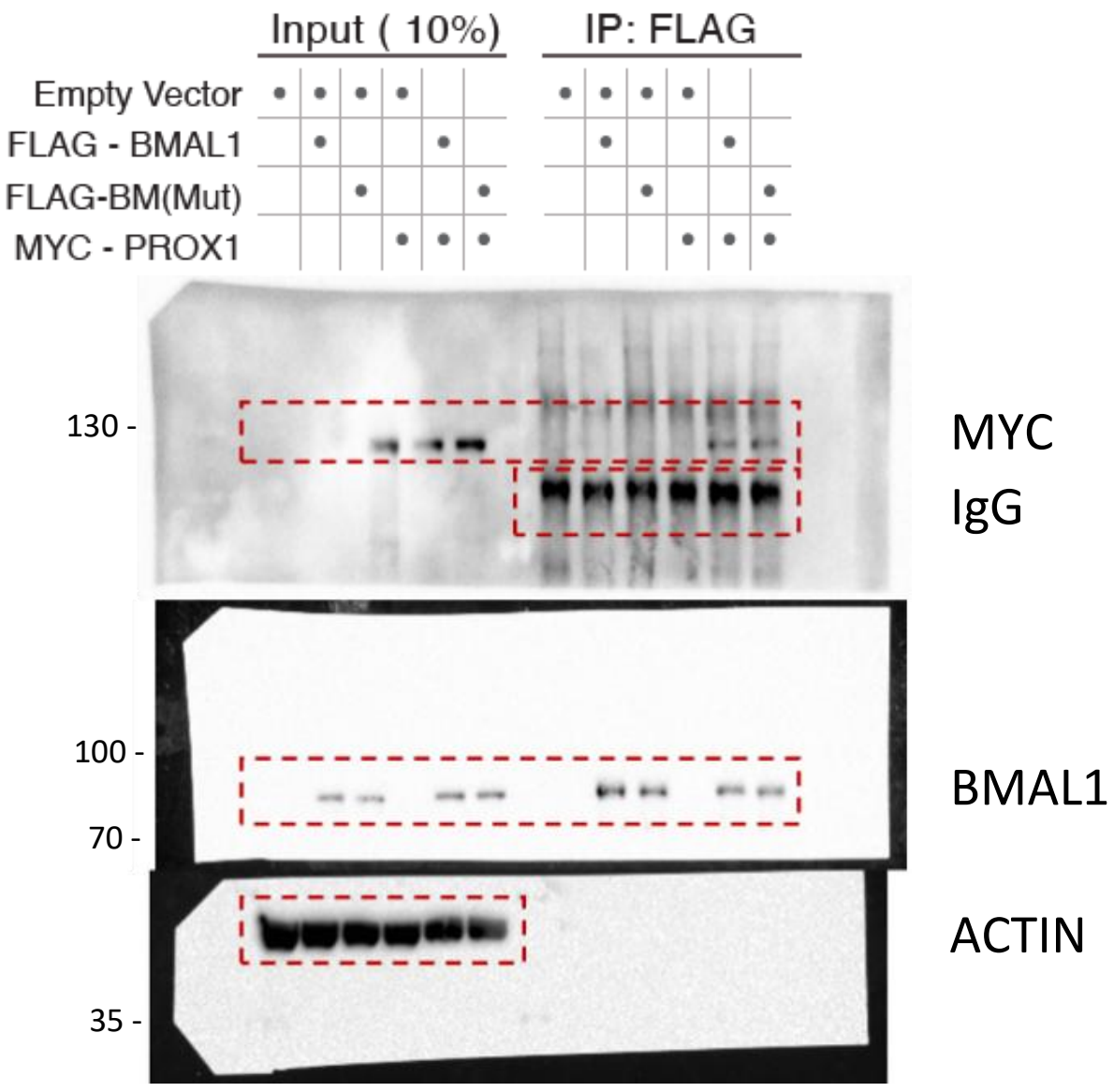

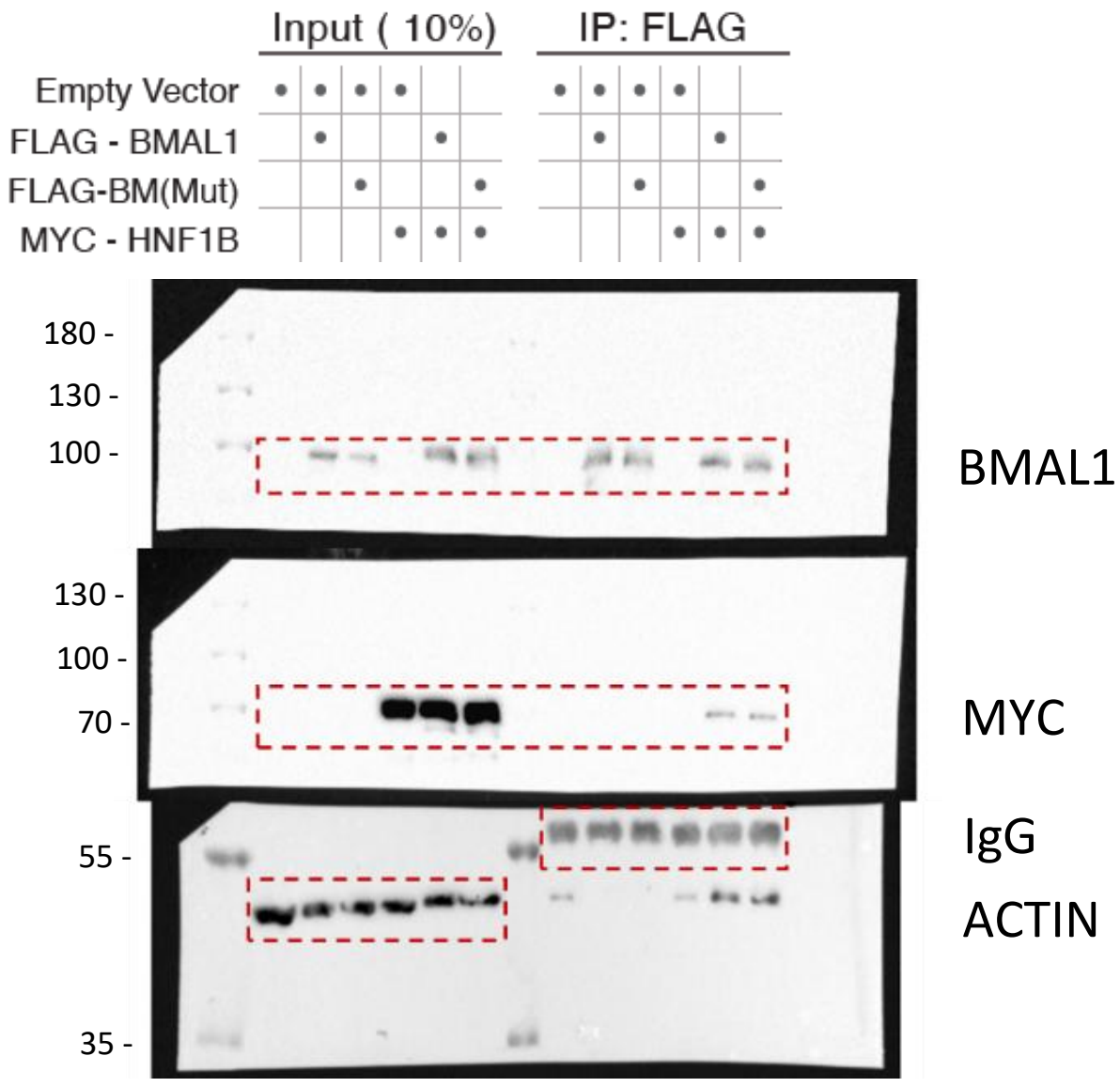

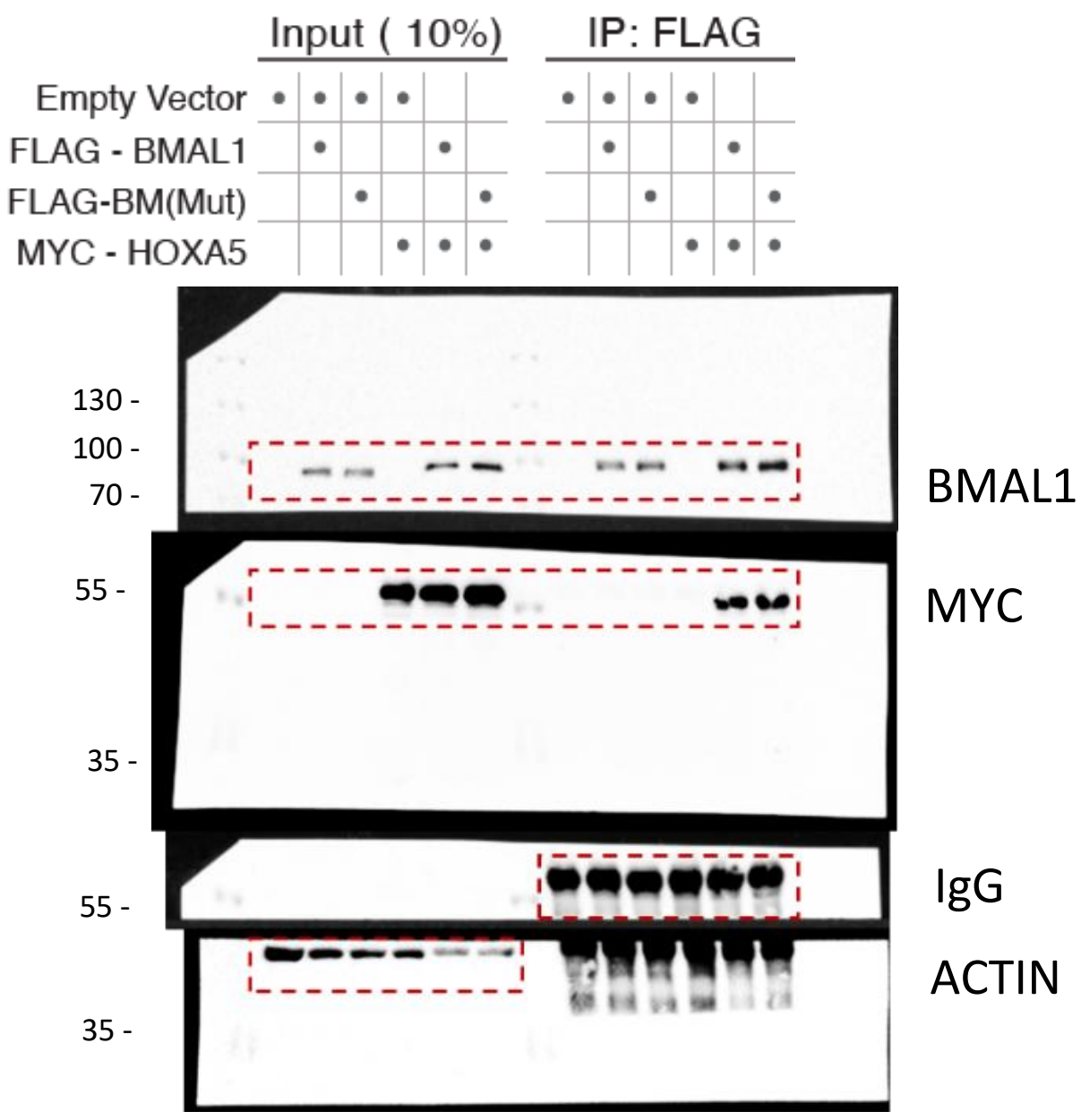

Unprocessed Immunoblot images relating to Extended Data Fig. 3g

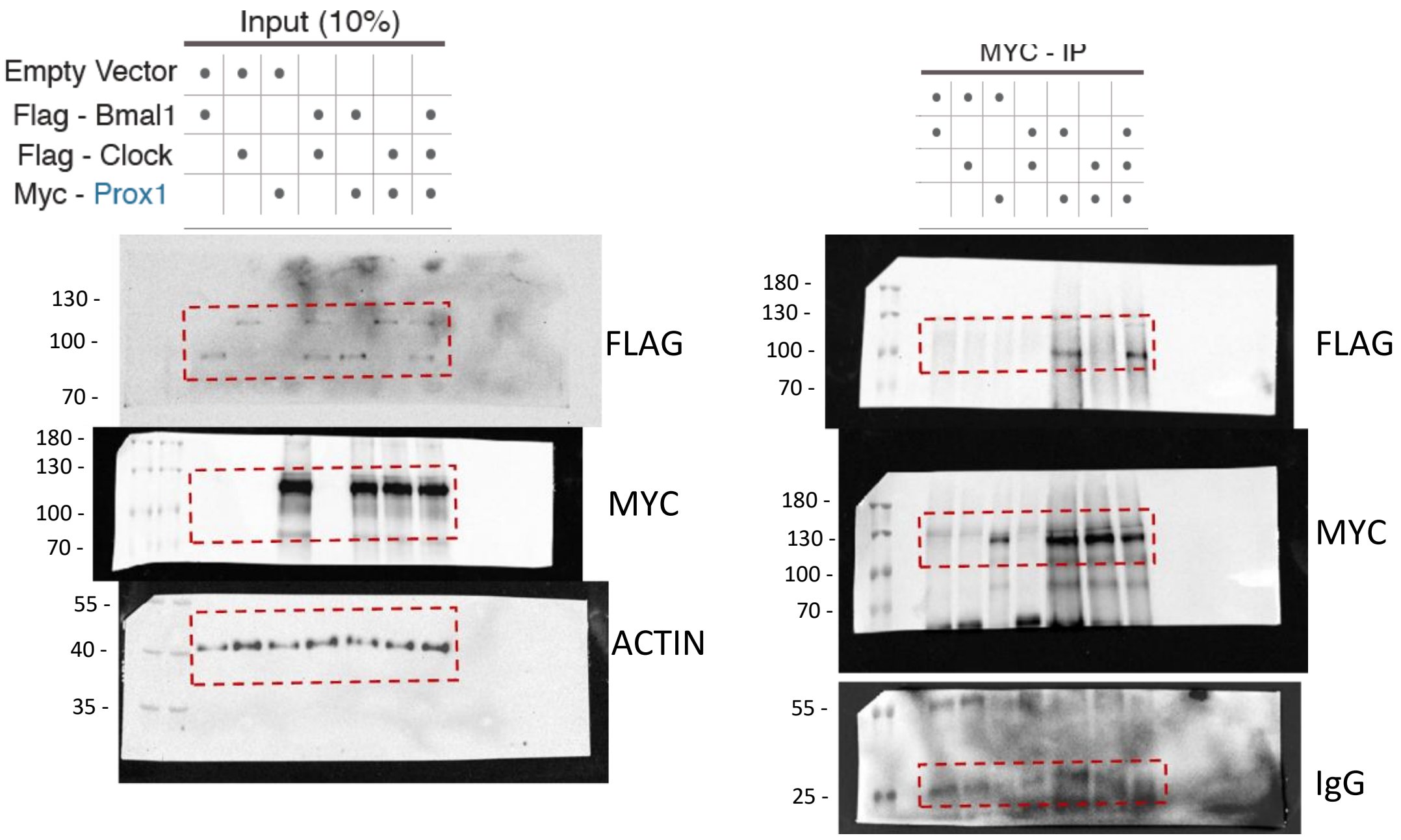

Unprocessed Immunoblot images relating to Extended Data Fig. 6a

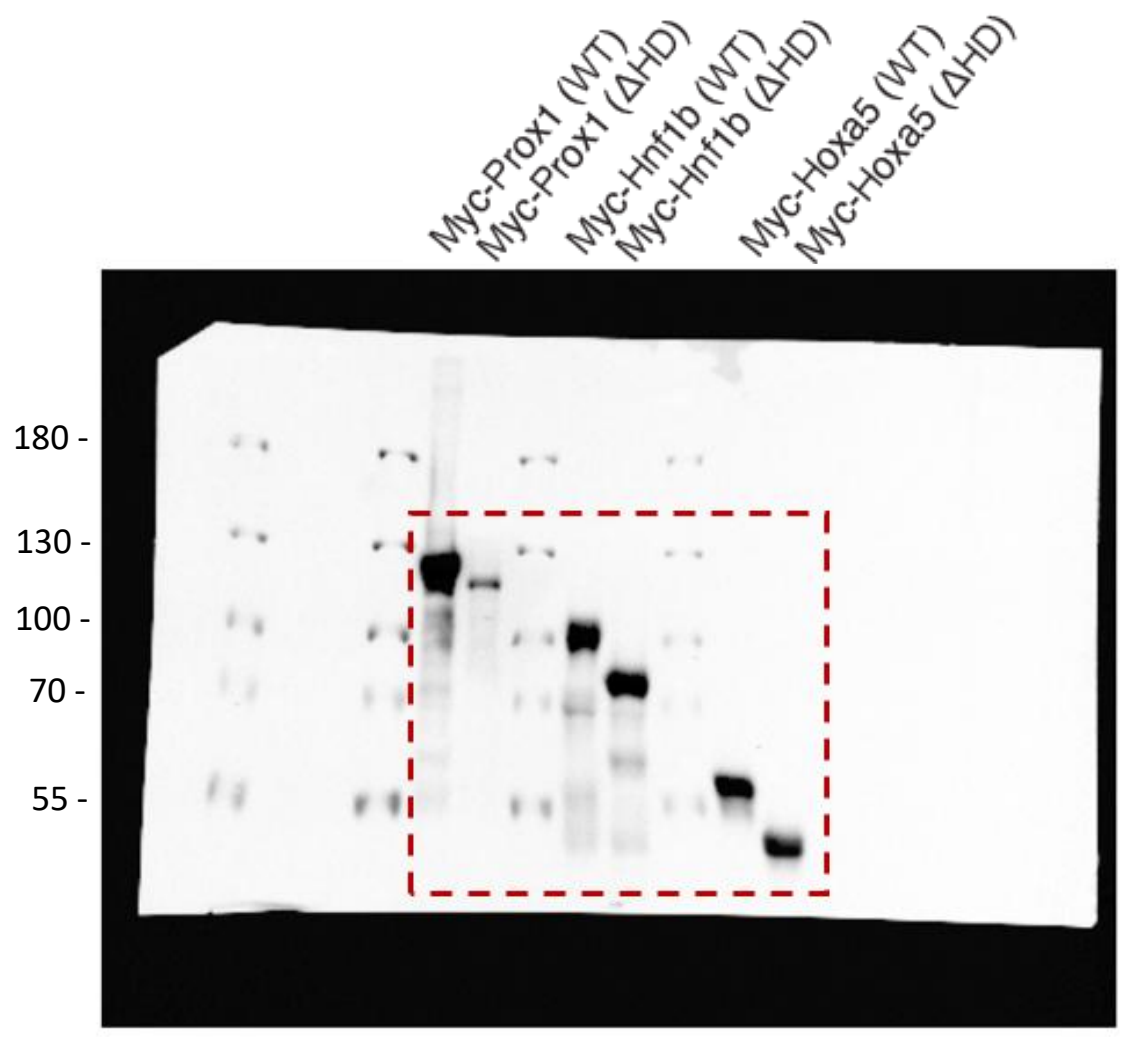

MYC

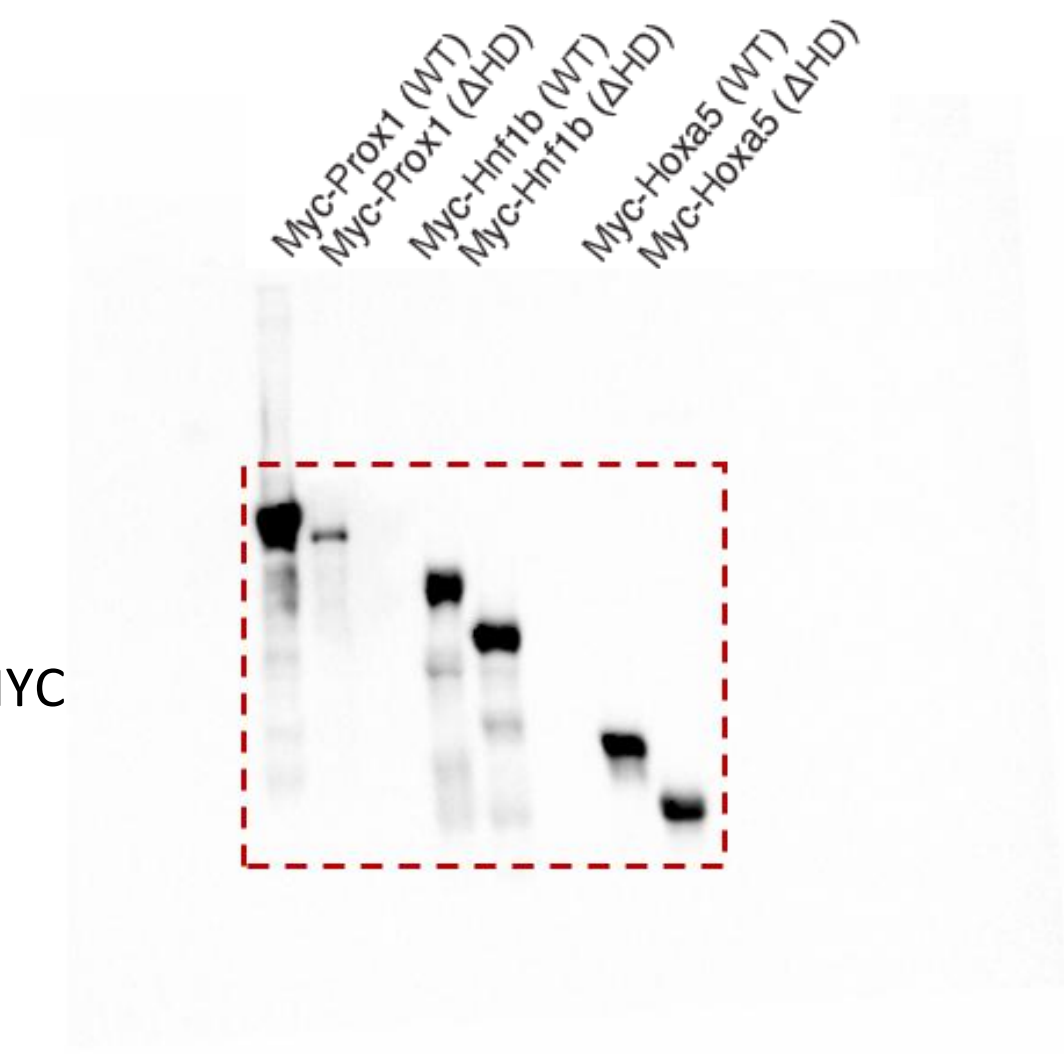

Unprocessed Immunoblot images relating to Extended Data Fig. 6c

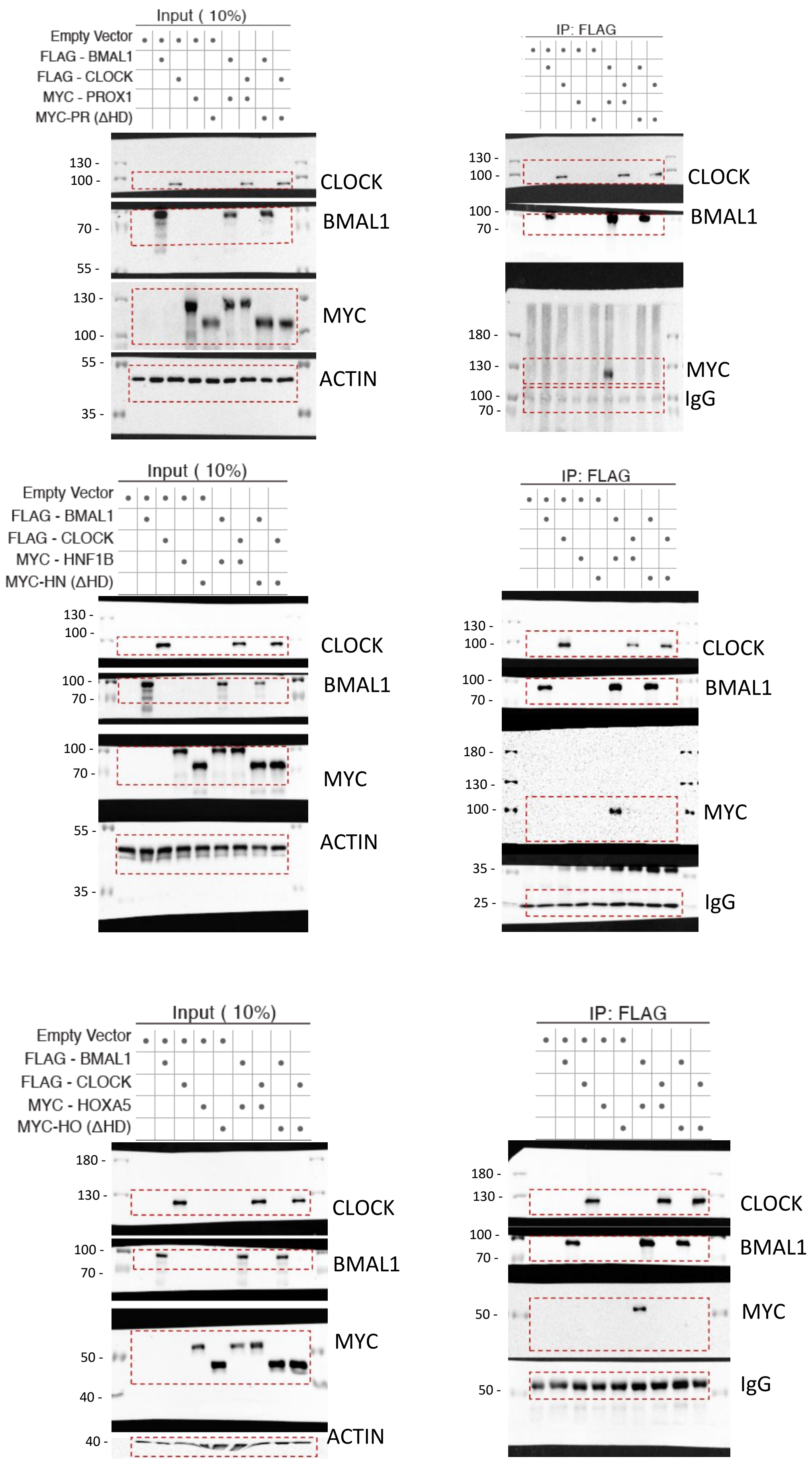

Unprocessed Immunoblot images relating to Extended Data Fig. 7a

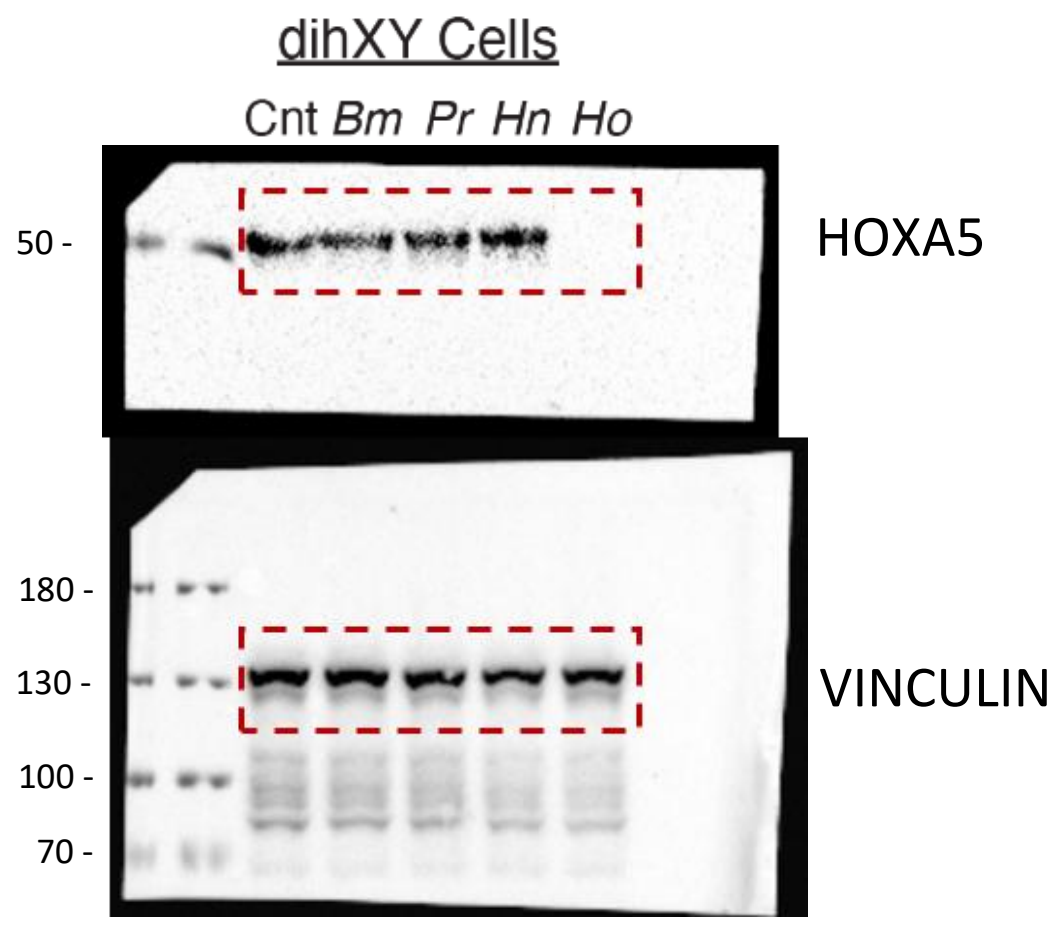

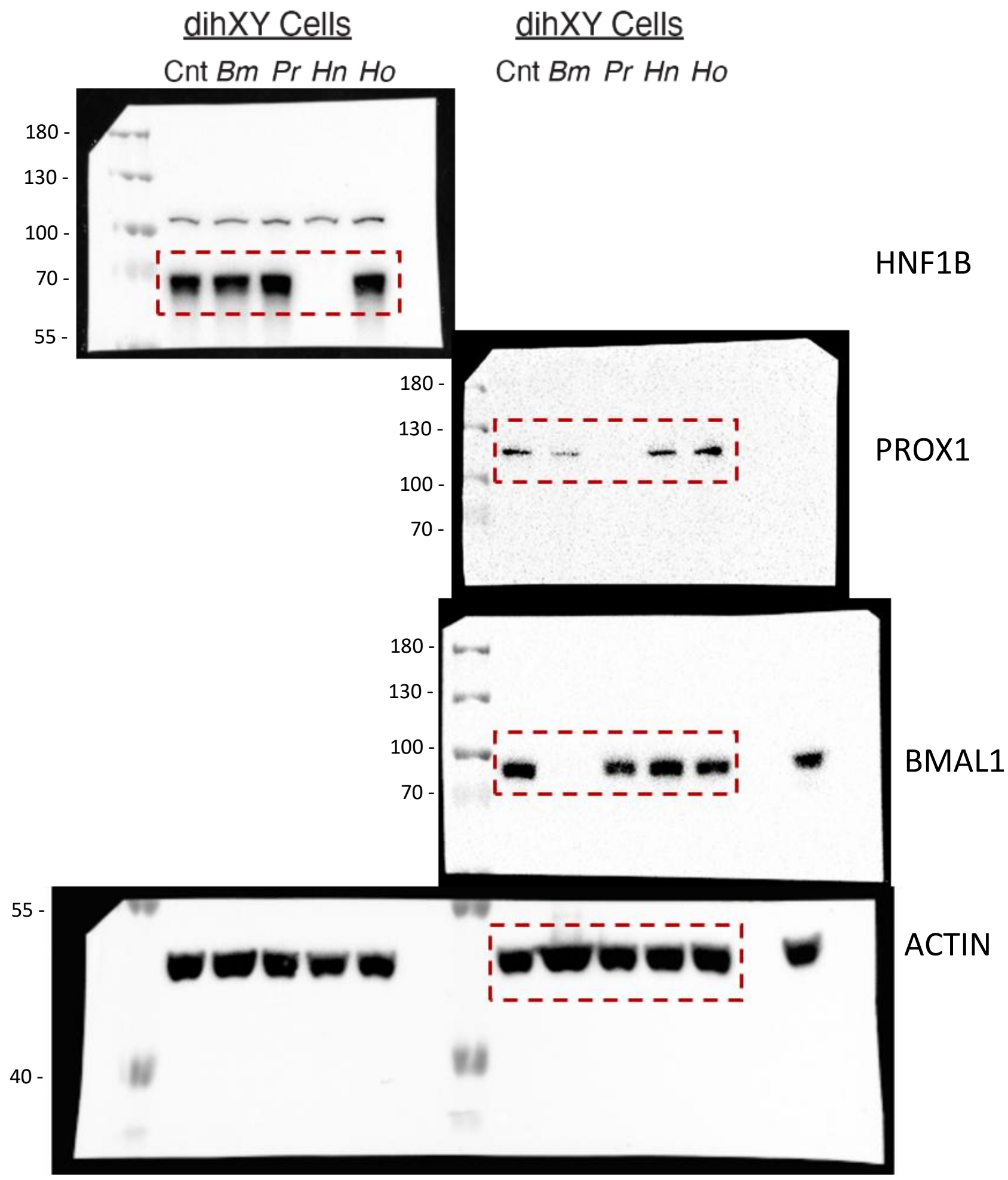

Unprocessed Immunoblot images relating to Extended Data Fig. 7b

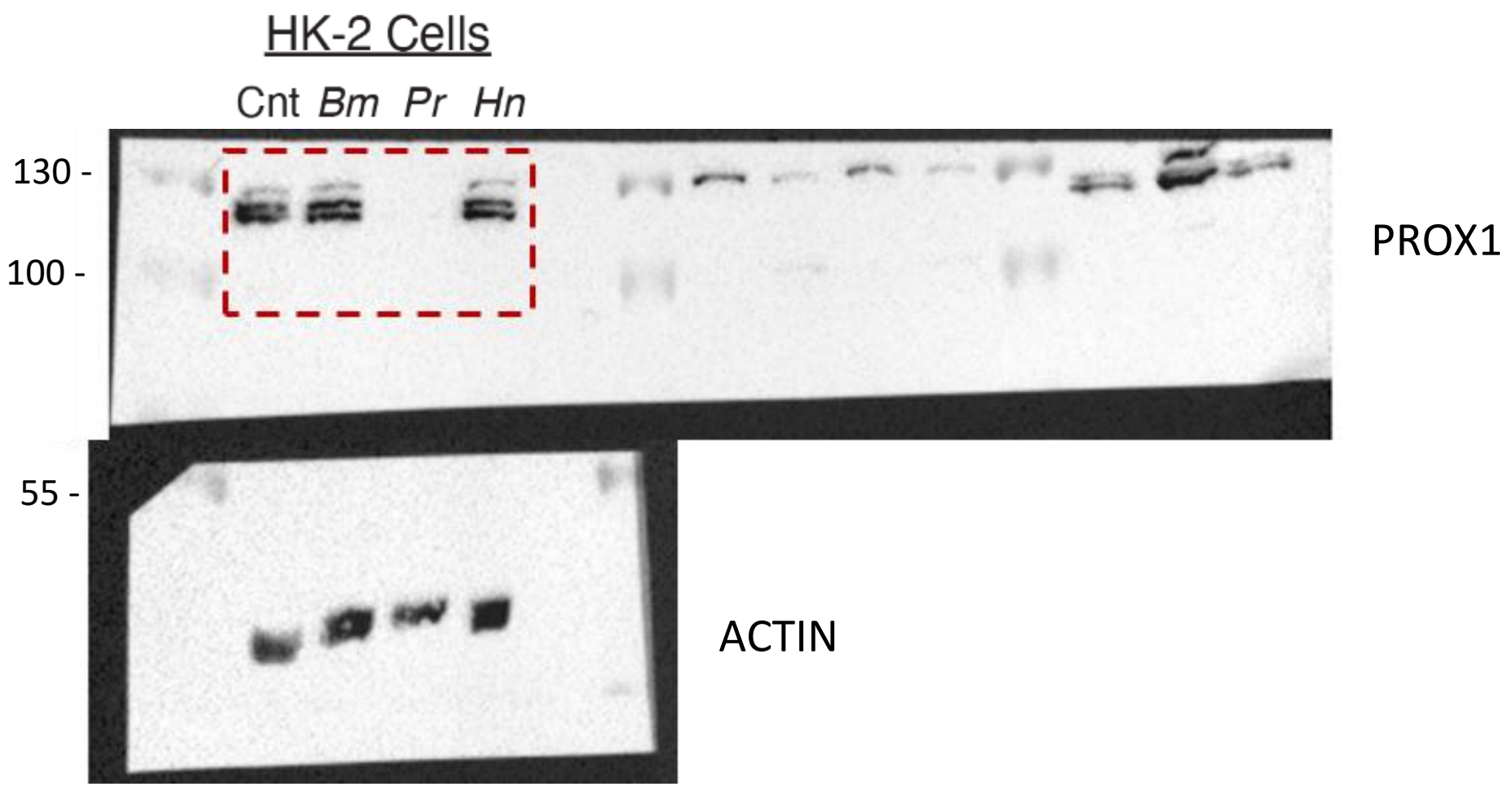

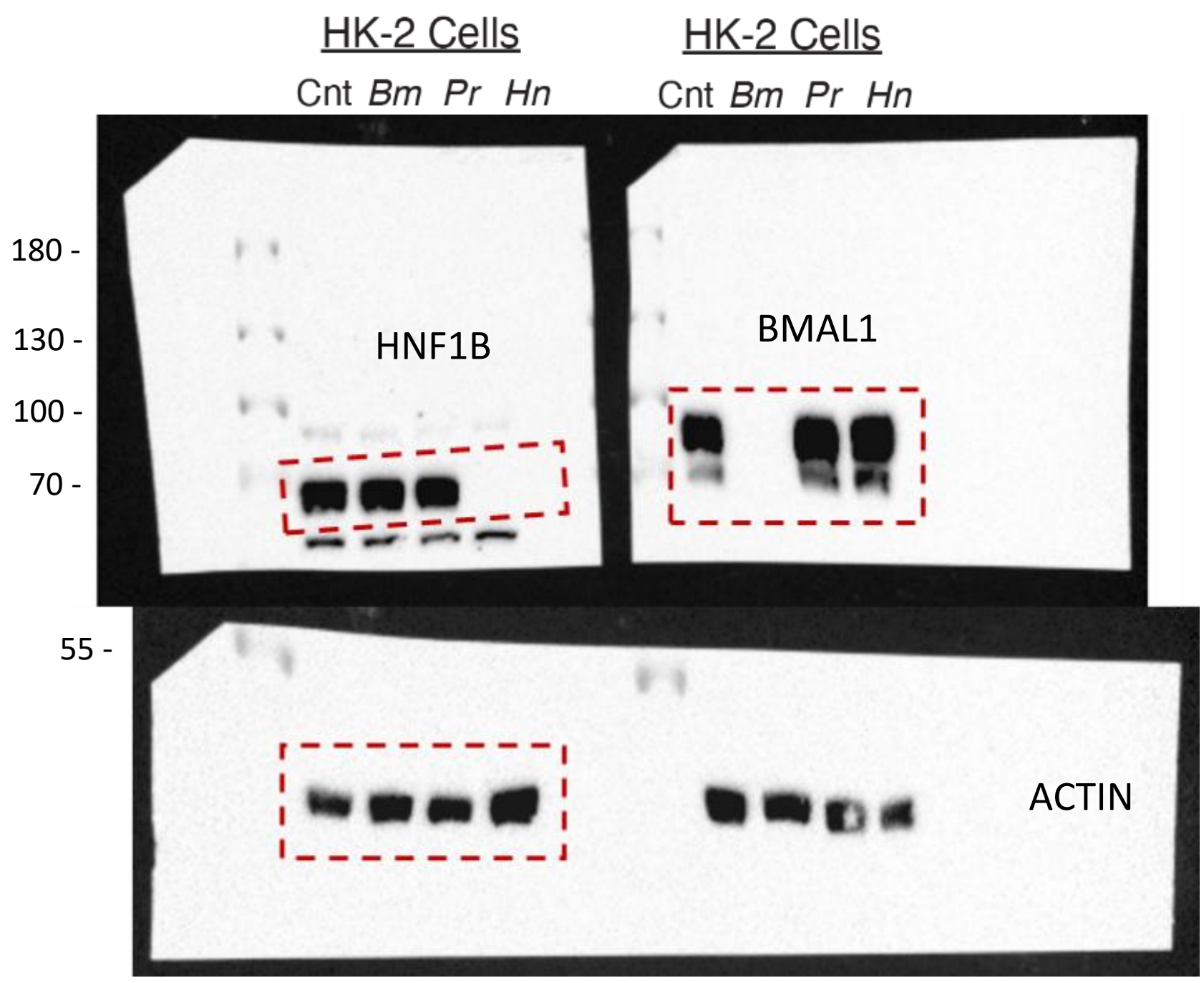

Unprocessed Immunoblot images relating to Extended Data Fig. 7c

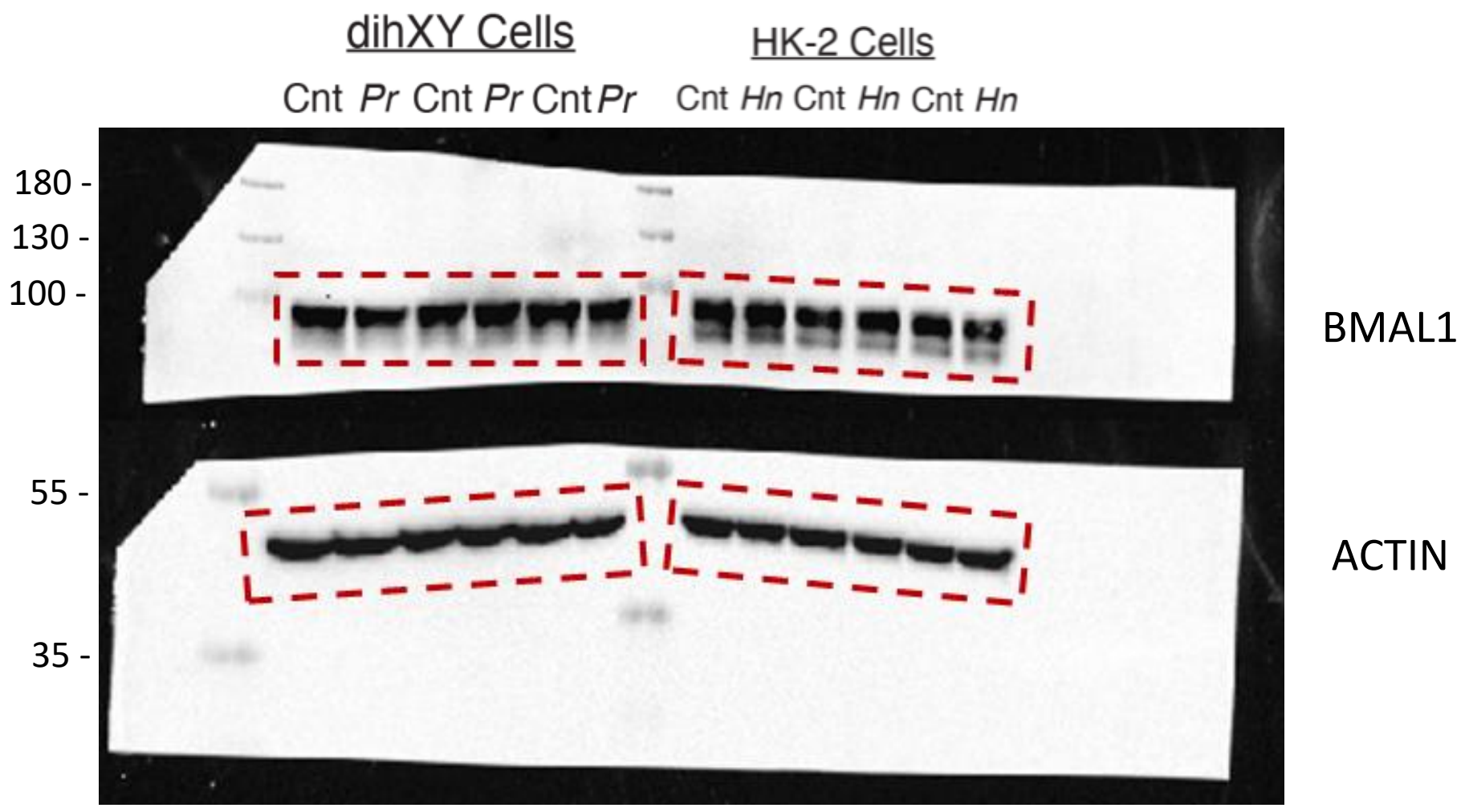

Unprocessed Immunoblot images relating to Extended Data Fig. 8a

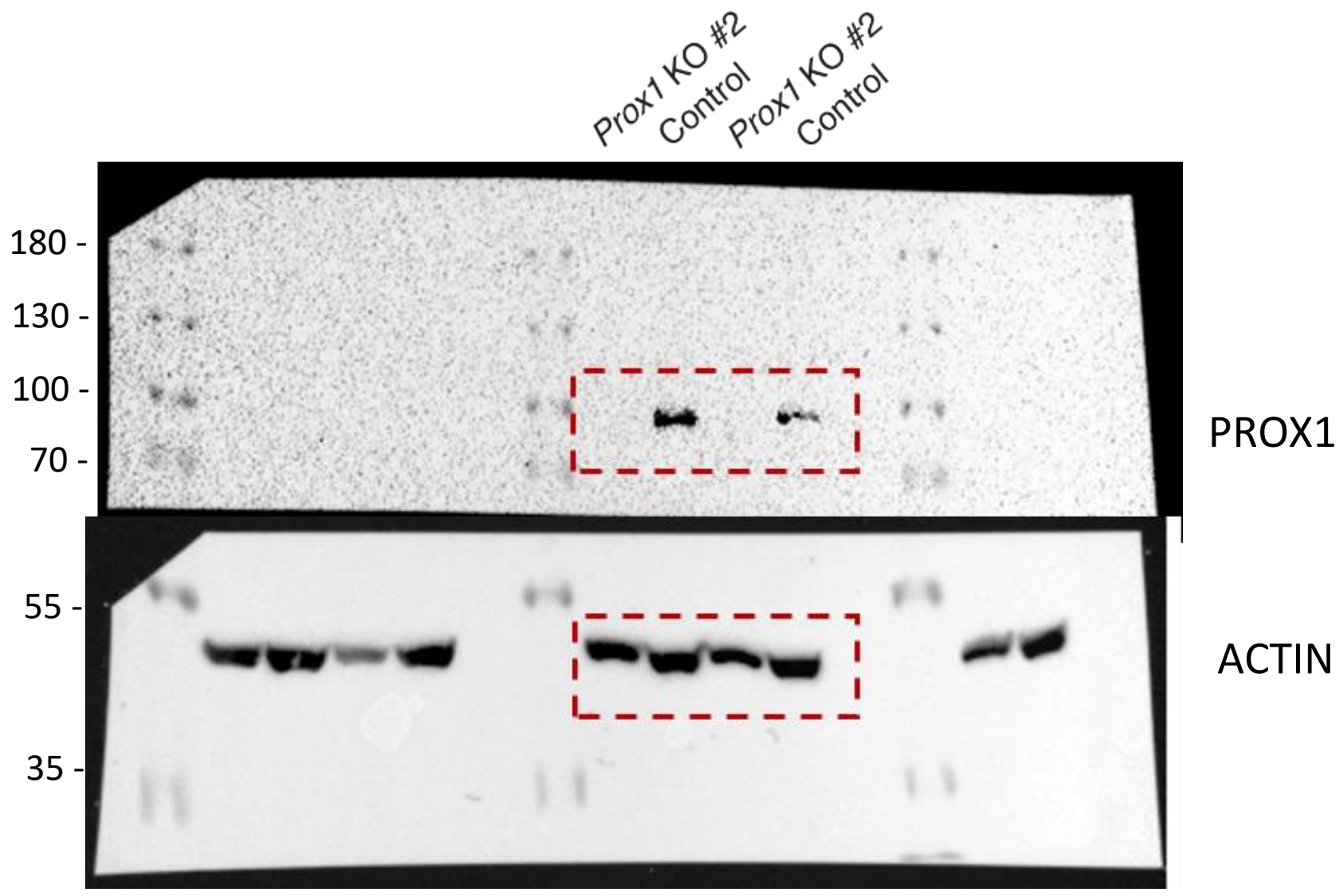

Supplement: Supplementary file 3 [file 41556_2026_2041_MOESM5_ESM.pdf]
